# Supplementary material for: Allosteric control of the bacterial ClpC/ClpP protease and its hijacking by antibacterial peptides
Source: EMBO J. 2025 Sep 29;44(21):6273–96. doi: 10.1038/s44318-025-00575-1 (PMC12583610; doi:10.1038/s44318-025-00575-1)
Supplement: Supplementary file 1 — Appendix [file 44318_2025_575_MOESM1_ESM.pdf]

Appendix for “Allosteric control of the bacterial ClpC/ClpP protease and its hijacking by antibacterial peptides”

Table of contents

**Appendix Figure S1**	**2**
**Appendix Figure S2**	**4**
**Appendix Figure S3**	**6**
**Appendix Figure S4**	**8**
**Appendix Figure S5**	**10**
**Appendix Figure S6**	**12**
**Appendix Figure S7**	**14**
**Appendix Figure S8**	**16**
**Appendix Figure S9**	**18**
**Appendix Figure S10**	**20**
**Appendix Figure S11**	**22**
**Appendix Figure S12**	**24**
**Appendix Table S1**	**26**
**Appendix Table S2**	**28**

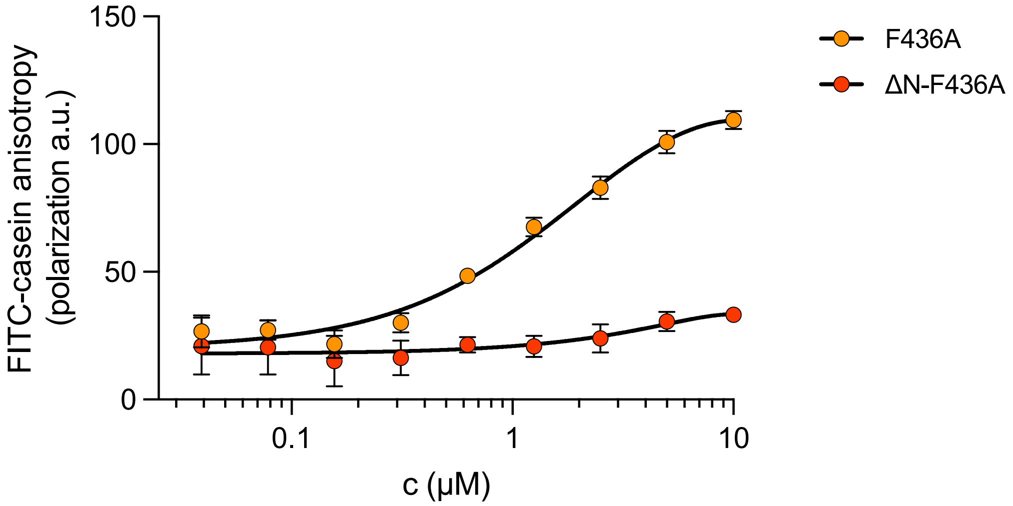
F436A
ΔN-F436A
FITC-casein anisotropy
(polarization a.u.)
150
100
50
0
0.1
1
10
c (μM)

Figure S1

Appendix Figure S1 - FITC-casein binds to the N-terminal domain of ClpC.

Binding of ClpC-F436A and ΔN-ClpC-F436A to FITC-casein was monitored in presence of ATPγS and increasing ClpC concentrations by determining changes in FITC-casein anisotropy (n = 4). Data information: SDs are based on two independent experiments including two technical replicates.

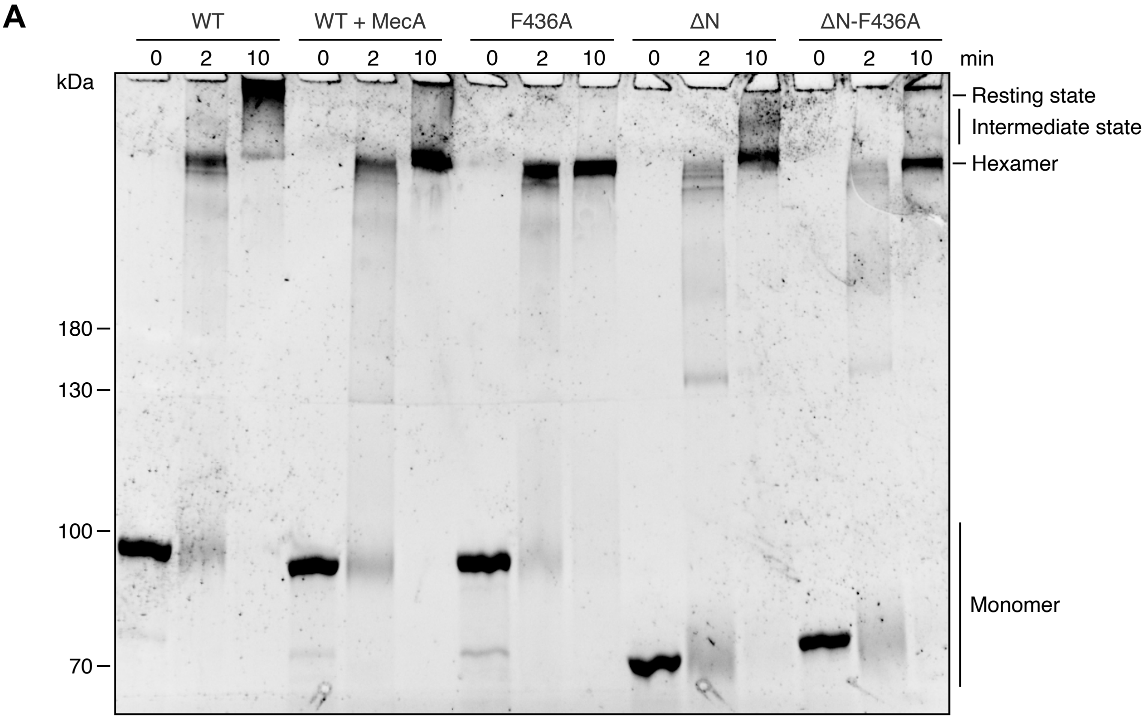
A
WT
WT + MecA
F436A
ΔN
ΔN-F436A
0
2
10
min
kDa
180
130
100
70
Resting state
Intermediate state
Hexamer
Monomer

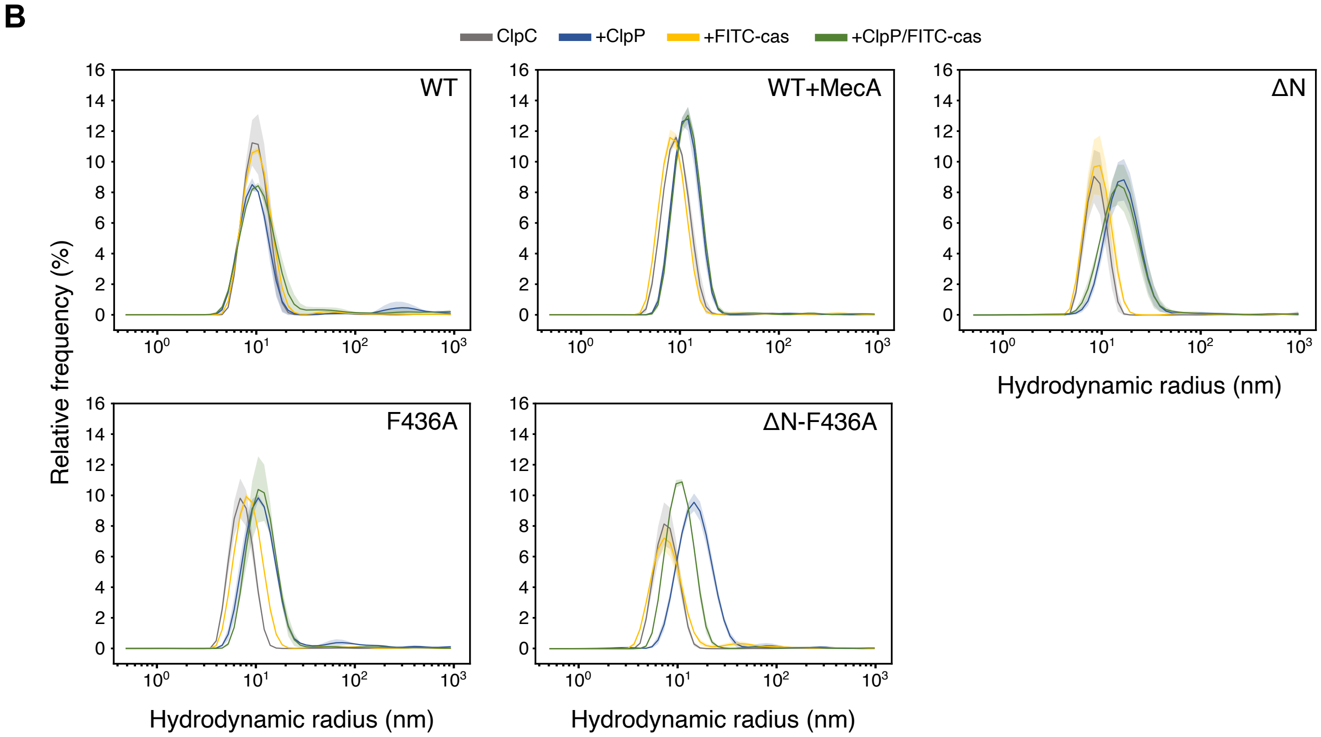
B
ClpC
+ClpP
+FITC-cas
+ClpP/FITC-cas
WT
WT+MecA
ΔN
F436A
ΔN-F436A
Relative frequency (%)
Hydrodynamic radius (nm)

Figure S2

Appendix Figure S2 - Deletion of the N-terminal domain modulates ClpC assembly state and allows for ClpP association.

A Glutaraldehyde (GA) crosslinking of ClpC-WT and indicated mutants was performed in presence of ATPγS. Crosslinking reactions were analyzed before (0 min) and after (2/10 min) GA addition by SDS-PAGE. Crosslink product identities are indicated. A protein standard (kDa) is provided.

B Particle size distributions (% frequency) of DLS data (respective boxplots see Fig. 2A) were determined for ClpC-WT and indicated mutants in presence of 2 mM ATPγS and of ClpP and FITC-casein as indicated.

Data information: In (B), SDs (n≥50) are shown as shaded area.

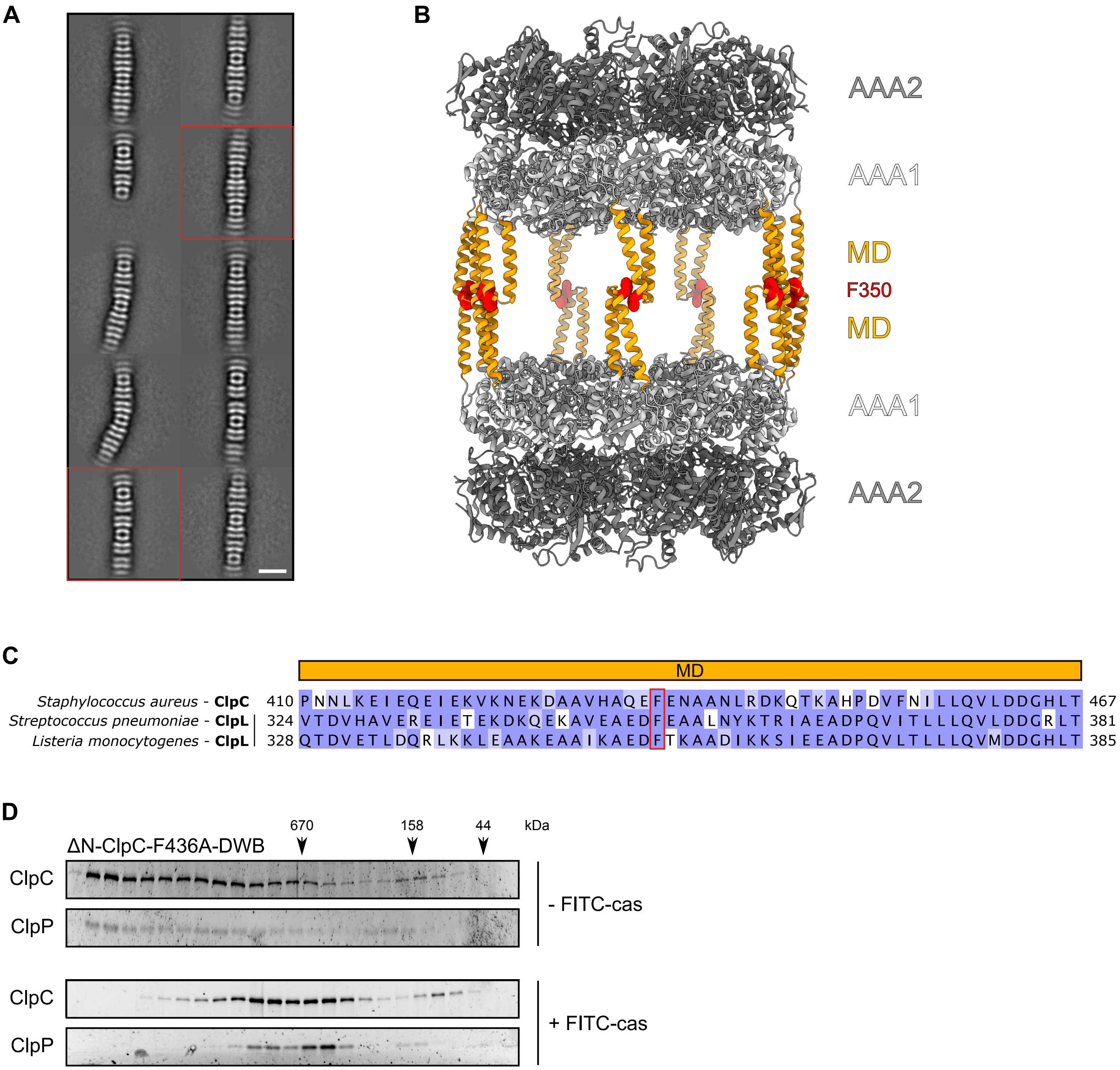
A
B
AAA2
AAA1
MD
F350
MD
AAA1
AAA2
C
MD
Staphylococcus aureus - **ClpC** 410 PNNLKEIEQEIEKVKNEKDAAVHAQEFENAANLRDKQTKAHPDVFNILLQVLDDGHLT 467
Streptococcus pneumoniae - **ClpL** 324 VTDVHAVEREIETEKDKQEKAVEAEDFEAALNYKTRIAEADPQVITLLLQVLDDGRLT 381
Listeria monocytogenes - **ClpL** 328 QTDVETLDQRLKKLEAAKEAAIKAEDFTKAADIKKSIEEADPQVLTLLLQVMDDGHLT 385
D
ΔN-ClpC-F436A-DWB
670
158
44
kDa
ClpC
ClpP
- FITC-cas
ClpC
ClpP
+ FITC-cas

Figure S3

Appendix Figure S3 - ΔN-ClpC forms ring dimers reminiscent of ClpL.

A Gallery of representative 2D class averages of ΔN-ClpC-E280A/E618A (ΔN-ClpC-DWB)/ClpP complexes. Red boxes indicate classes shown in Fig. 2C. Scale bar = 20 nm.

B Cryo-EM structure (pdb:6lt4) of the ClpL ring dimer, highlighting the middle domain (MD) in orange and the conserved crucial residue F350 in red.

C Multiple sequence alignment of the MD of *Staphylococcus aureus* ClpC with ClpL proteins of *Streptococcus pneumoniae* and *Listeria monocytogenes*. Similar and identical residues are highlighted in light and dark blue. The phenylalanine residues mediating MD-MD interactions crucial for resting state formation are framed in red.

D Complex formation of ATPase-deficient ΔN-ClpC-F436A-DWB (E280A/E618A) with ClpP in presence of 2 mM ATP was monitored by Superose 6 size-exclusion chromatography in absence and presence of substrate FITC-casein. Elution fractions were analyzed by SDS-PAGE and Sypro Ruby staining. The elution profile of a protein standard is provided.

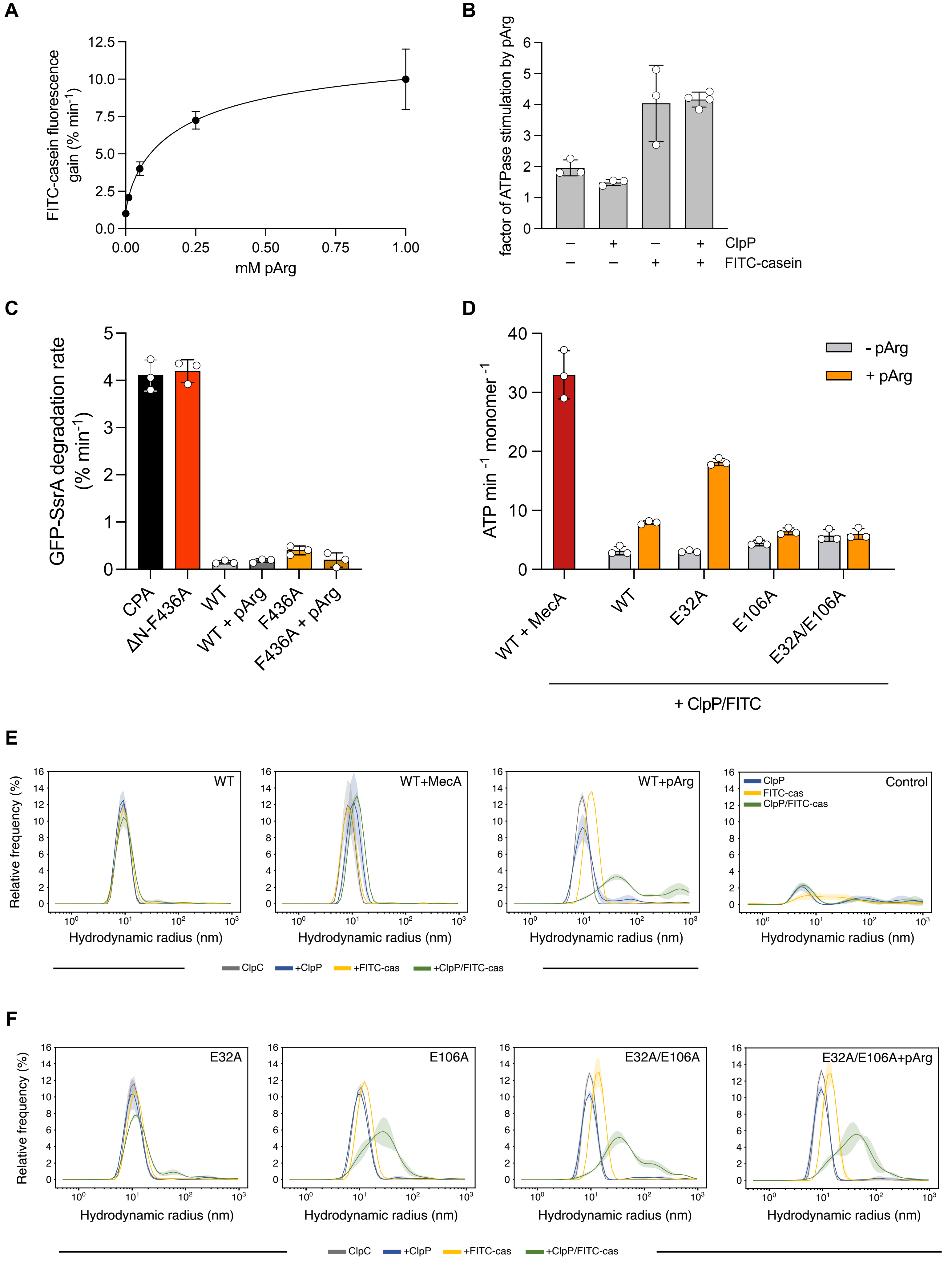
A
FITC-casein fluorescence gain (% min-1)
12.5
10.0
7.5
5.0
2.5
0.0
0.00
0.25
0.50
0.75
1.00
mM pArg
B
factor of ATPase stimulation by pArg
6
5
4
3
2
1
0
– + – + ClpP
– – + + FITC-casein
C
GFP-SsrA degradation rate (% min-1)
5
4
3
2
1
0
CPA
ΔN-F436A
WT
WT + pArg
F436A
F436A + pArg
D
ATP min -1 monomer -1
40
30
20
10
0
- pArg
+ pArg
WT + MecA
WT
E32A
E106A
E32A/E106A
+ ClpP/FITC
E
Relative frequency (%)
Hydrodynamic radius (nm)
WT
WT+MecA
WT+pArg
Control
ClpP
FITC-cas
ClpP/FITC-cas
ClpC
+ClpP
+FITC-cas
+ClpP/FITC-cas
F
Relative frequency (%)
Hydrodynamic radius (nm)
E32A
E106A
E32A/E106A
E32A/E106A+pArg
ClpC
+ClpP
+FITC-cas
+ClpP/FITC-cas

Figure S4

Appendix Figure S4 - pArg binding or mutating pArg1/2-binding sites leads to ClpC activation.

A FITC-casein degradation activities (% fluorescence intensity increase/min) of ClpC-WT were determined in presence of increasing pArg concentrations (n = 3).

B Factors of ClpC ATPase activity increase upon addition of 250 µM pArg were determined in absence and presence of indicated components.

C GFP-SsrA degradation rates were determined in presence of ClpP, ClpC-WT and indicated ClpC mutants and absence or presence of 0.25 mM pArg.

D ATPase activities of ClpC-WT and indicated mutants were determined in presence of ClpP and FITC-casein without and with 250 µM pArg as indicated.

E, F Particle size distributions (% frequency) of DLS data (respective boxplots see Fig. 4A) were determined for ClpC-WT and indicated mutants in presence of ClpP and FITC-casein as indicated. Control reactions showing ClpP, FITC-casein and ClpP + FITC-casein in absence of ClpC are provided.

Data information: In (A-D), SDs are based on at least three independent experiments. In (E-F), SDs ($n \geq 54$) are shown as shaded area.

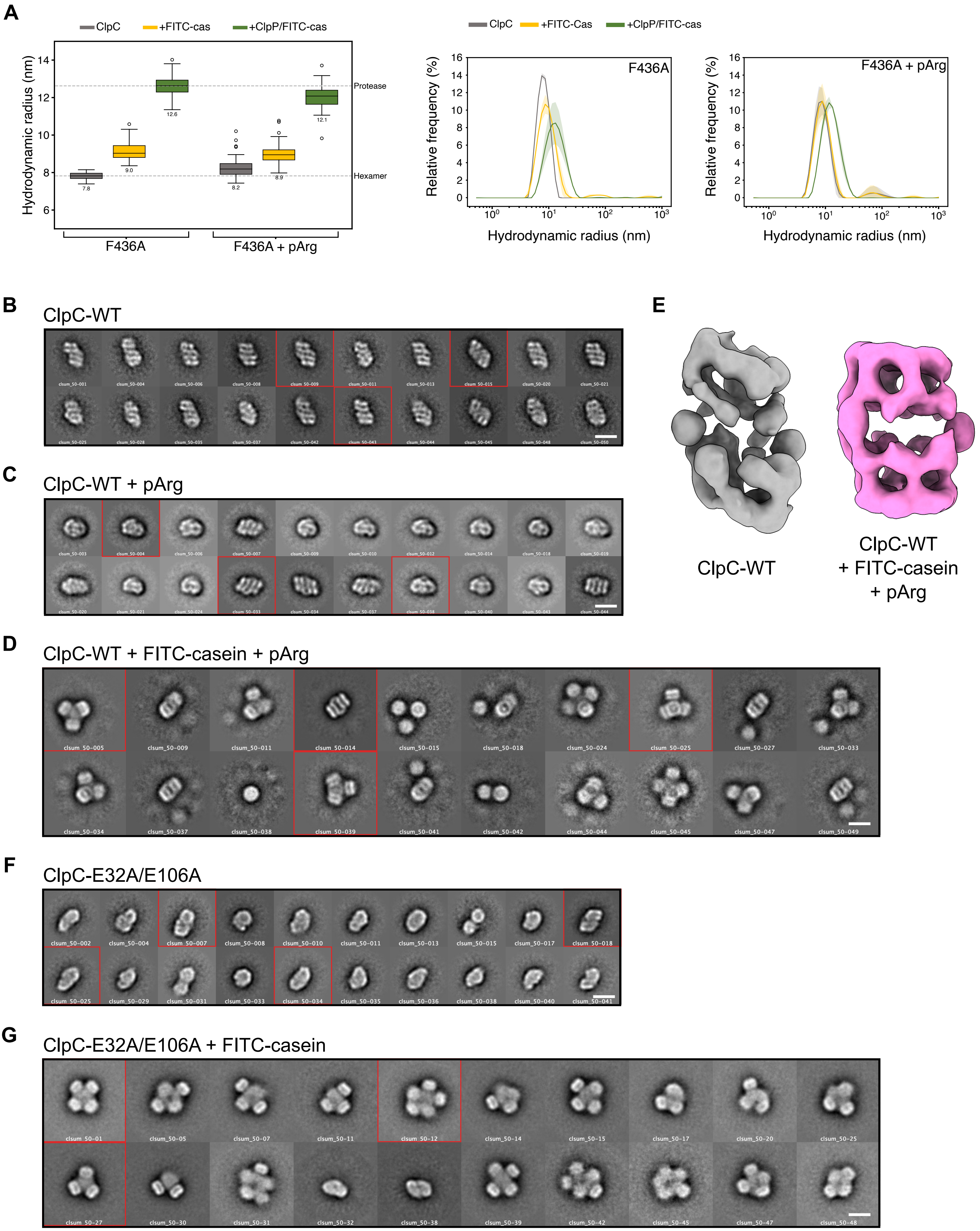
A
ClpC
+FITC-cas
+ClpP/FITC-cas
Hydrodynamic radius (nm)
Protease
Hexamer
7.8
9.0
12.6
8.2
8.9
12.1
F436A
F436A + pArg
ClpC
+FITC-Cas
+ClpP/FITC-cas
Relative frequency (%)
Hydrodynamic radius (nm)
B
ClpC-WT
C
ClpC-WT + pArg
D
ClpC-WT + FITC-casein + pArg
E
ClpC-WT
ClpC-WT
+ FITC-casein
+ pArg
F
ClpC-E32A/E106A
G
ClpC-E32A/E106A + FITC-casein

Figure S5

Appendix Figure S5 - pArg binding or mutating pArg1/2-binding sites changes ClpC assembly state.

A Hydrodynamic radii of ClpC-F436A without and with 250 µM pArg were determined by DLS measurements in absence and presence of ClpP and substrate FITC-casein as indicated. Assembly identities are indicated. The particle size distributions (% frequency) of respective measurements are provided.

B-D Gallery of representative 2D class averages of ClpC-WT (B) bound to pArg (C) or pArg and FITC-casein (D).

E Low-resolution density maps of the ClpC-WT resting state (see Appendix Fig. S5B) and ClpC-WT ring dimers forming upon addition of pArg + FITC-casein (see Appendix Fig. S5D).

F, G Gallery of representative 2D class averages of ClpC E32A/E106A in absence (F) and presence of substrate FITC-casein (G).

Data information: In (A), data are represented as described in Figure 2. Standard deviations ($n \geq 60$) are shown as shaded area. In (B-D, F-G) red boxes indicate classes shown in Fig. 4C/D. Scale bar = 20 nm.

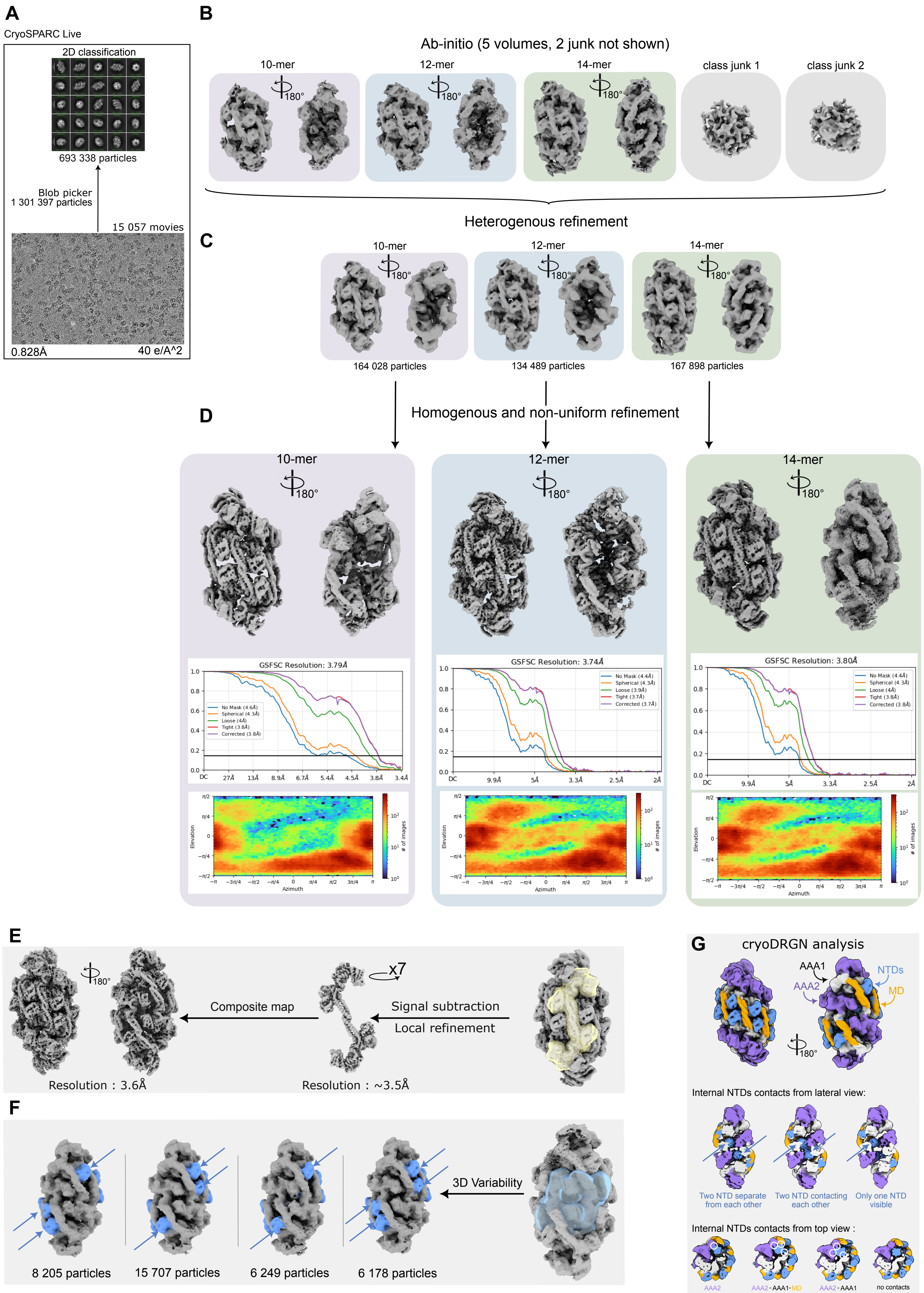
A
CryoSPARC Live
2D classification
693 338 particles
Blob picker
1 301 397 particles
15 057 movies
0.828Å
40 e/A^2
B
Ab-initio (5 volumes, 2 junk not shown)
10-mer
12-mer
14-mer
class junk 1
class junk 2
180°
Heterogenous refinement
C
164 028 particles
134 489 particles
167 898 particles
D
Homogenous and non-uniform refinement
GSFSC Resolution: 3.79Å
GSFSC Resolution: 3.74Å
GSFSC Resolution: 3.80Å
No Mask
Spherical
Loose
Tight
Corrected
Elevation
Azimuth
# of images
E
Composite map
Signal subtraction
Local refinement
x7
Resolution : 3.6Å
Resolution : ~3.5Å
F
3D Variability
8 205 particles
15 707 particles
6 249 particles
6 178 particles
G
cryoDRGN analysis
AAA1
AAA2
NTDs
MD
Internal NTDs contacts from lateral view:
Two NTD separate from each other
Two NTD contacting each other
Only one NTD visible
Internal NTDs contacts from top view :
AAA2
AAA2 - AAA1- MD
AAA2 - AAA1
no contacts

Figure S6

Appendix Figure S6 - Cryo-EM processing workflow of the ClpC WT dataset.

A Movies were processed on-the-fly during data collection using CryoSPARC Live for motion correction (not shown), CTF estimation (not shown), particle picking and 2D classification.
B Several rounds of 2D classification and *ab-initios* were performed to separate particles from noise (junk).

C 3D classification with alignment (heterogeneous refinement) was performed using the clean particles and *ab-initio* volumes of the 10-mer, 12-mer, 14-mer were generated.

D Each of the resulting resting state volumes were further refined by homogeneous (not shown) and non-uniform refinement. The Fourier Shell Correlation (GFSC) curves and viewing distribution plots are shown for each of the oligomeric state volumes.

E The 14-mer volume was further processed with signal subtraction and local refinement of each dimeric unit. The seven different locally refined dimeric units where combined into one map.

F The flexible NTDs of the "back" side of the 14-mer were further analyzed with 3D variability analysis and a mask around that region. Results of the 3D variability analysis demonstrate the different positions of the NTDs (highlighted in blue) in the "back side".

G To better understand the continuous motions of the NTDs, cryoDRGN was used. Various contacts between different domains and the internal NTDs can be observed.

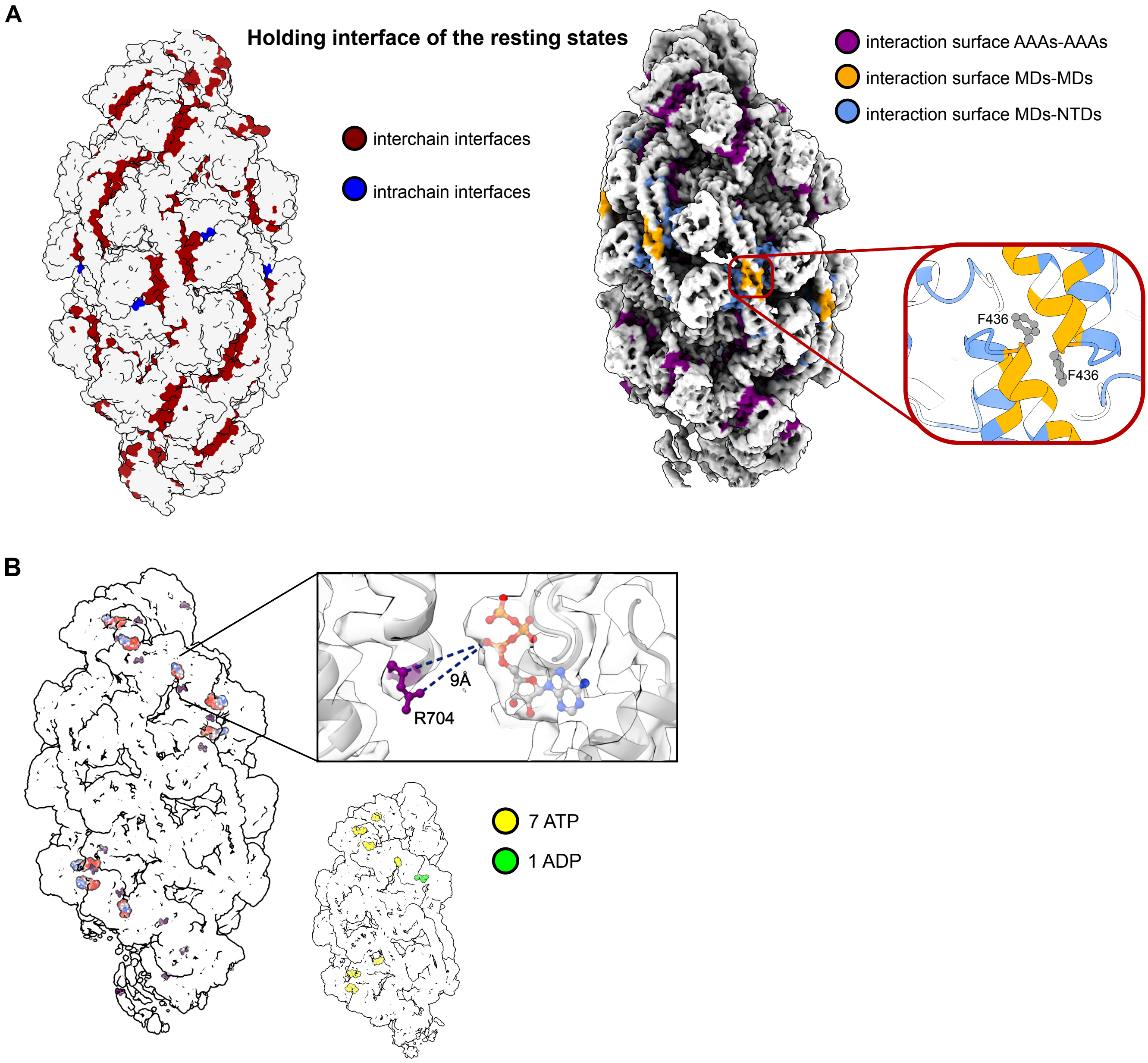
A
Holding interface of the resting states
interchain interfaces
intrachain interfaces
interaction surface AAAs-AAAs
interaction surface MDs-MDs
interaction surface MDs-NTDs
F436
F436
B
9Å
R704
7 ATP
1 ADP

Figure S7

Appendix Figure S7 - Depiction of the different interaction surface and nucleotide state in ClpC 14-mer resting state.

A Interaction surfaces involving inter- or intra-subunit contacts are depicted in brick red and blue, respectively. Interaction surface involving AAA-AAA (purple), MD-MD (orange) and MD-NTD (blue) domains are highlighted in the ClpC 14-mer resting state. The MD-MD region and the well characterized residues F436 located at the tip of the MD are highlighted.

B Position of the identifiable nucleotides in the 14-mer resting states. A total of 8 nucleotides were built, 7 ATPs (yellow) and 1 ADP (green), all located in the AAA2 domains. The average distance between nucleotide and neighboring arginine finger R704 (purple) is 9 Å.

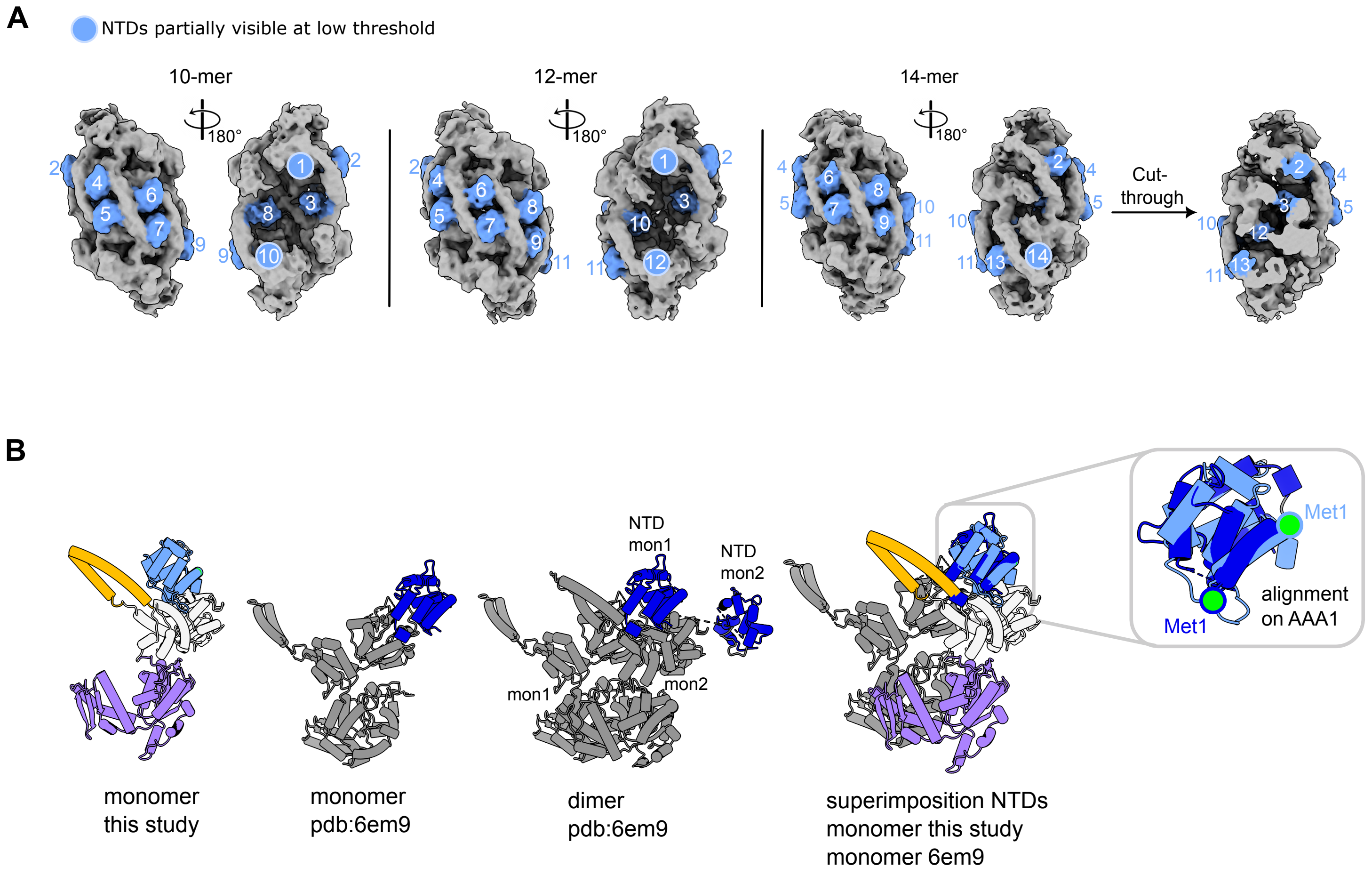
A
NTDs partially visible at low threshold
10-mer
180°
12-mer
180°
14-mer
180°
Cut-through
B
NTD mon1
NTD mon2
mon1
mon2
Met1
Met1
alignment on AAA1
monomer this study
monomer pdb:6em9
dimer pdb:6em9
superimposition NTDs monomer this study monomer 6em9

Figure S8

Appendix Figure S8 - Comparisons between the positioning of the NTDs in the previous (pdb:6em9) and in the current structure.

A Positions of the NTDs in the different oligomeric assemblies (10-/12-/14-mer). For each state, NTDs are counted and displayed in light blue. Circles are shown to represent NTDs that are only visible at low sigma threshold of the map.

B The current positioning of the NTD (light blue) and previous positioning (dark blue) are compared by superimposing on the AAA1 domain one subunit of each structure. The current assignment of the NTD is rotated by 180° relative to the previous assignment. This change is visualized by the different position of the same first methionine residue (Met1; green) between the two structures. The fold of the NTD is also different between the two structures as the previous structure was based on homology modeling available at the time and the current structure is from an AlphaFold2 prediction.

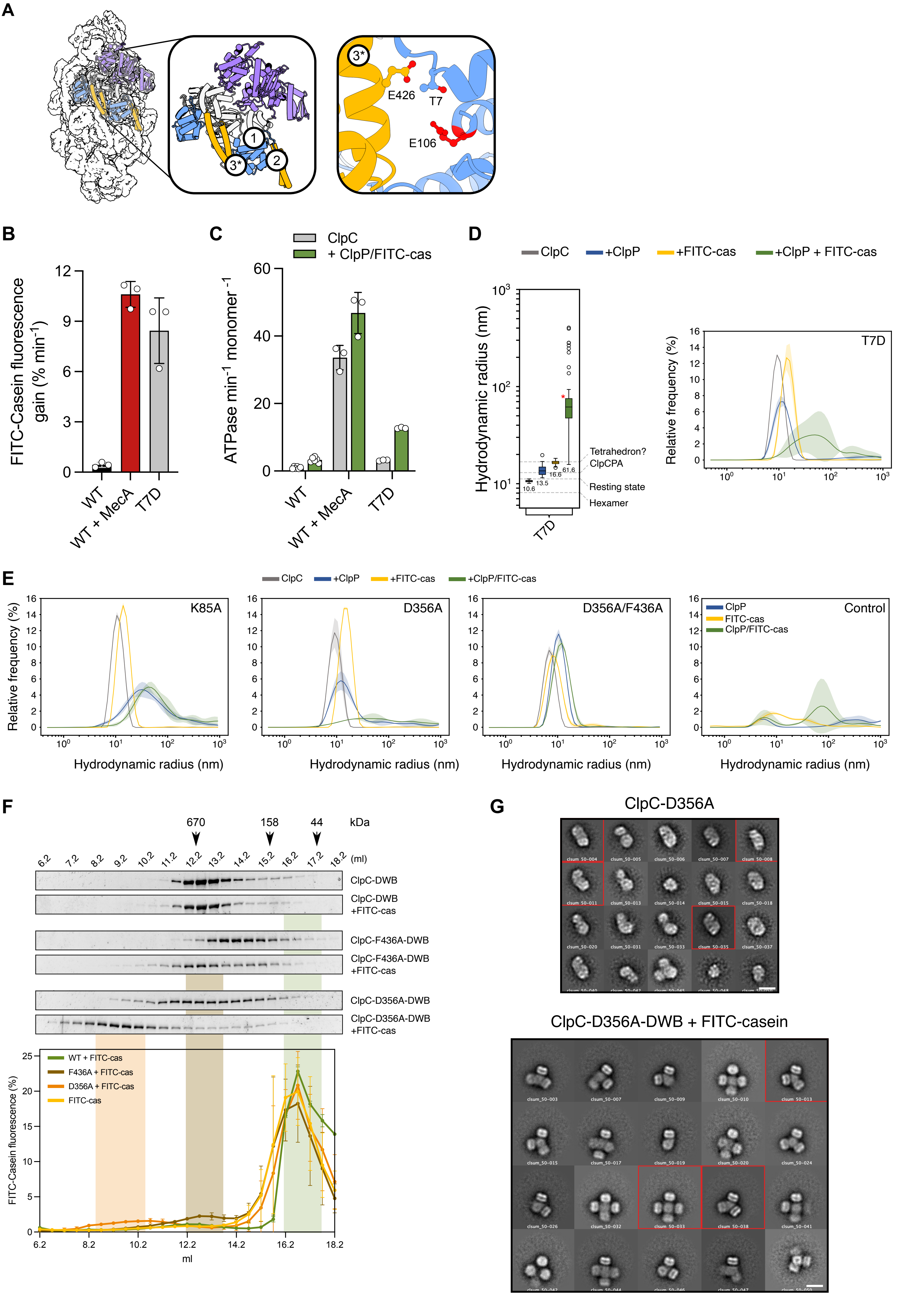

A
E426
T7
E106
B
FITC-Casein fluorescence gain (% min^{-1})
WT
WT + MecA
T7D
C
ClpC
+ ClpP/FITC-cas
ATPase min^{-1} monomer^{-1}
D
ClpC
+ClpP
+FITC-cas
+ClpP + FITC-cas
Hydrodynamic radius (nm)
Tetrahedron?
ClpCPA
Resting state
Hexamer
Relative frequency (%)
E
+ClpP/FITC-cas
K85A
D356A
D356A/F436A
Control
ClpP
FITC-cas
ClpP/FITC-cas
F
670
158
44
kDa
(ml)
ClpC-DWB
ClpC-DWB +FITC-cas
ClpC-F436A-DWB
ClpC-F436A-DWB +FITC-cas
ClpC-D356A-DWB
ClpC-D356A-DWB +FITC-cas
WT + FITC-cas
F436A + FITC-cas
D356A + FITC-cas
FITC-cas
FITC-Casein fluorescence (%)
ml
G
ClpC-D356A
ClpC-D356A-DWB + FITC-casein

Figure S9

Appendix Figure S9 - NTD-AAA1 anchoring points are crucial for resting state formation.

A Two adjacent subunits within the resting state are highlighted. Three NTD anchoring points highlighting contributing interacting residues are displayed. The NTD-MD* (neighboring MD) contact (3*) comprises the pArg1 site involving a interaction of T7-E426.

B FITC-casein degradation activities (% fluorescence intensity increase/min) of ClpC-WT and T7D were determined in presence of ClpP and ClpC-WT + MecA.

C ATPase activities of ClpC-WT and ClpC-T7D were determined in absence and presence of ClpP and FITC-casein.

D Hydrodynamic radii of ClpC-T7D were determined by DLS measurements in absence and presence of ClpP and substrate FITC-casein as indicated. Assembly identities are indicated. Red asterisks indicate heterogenous samples with multiple peaks, for which the value with the smallest hydrodynamic radius was included for data representation. The particle size distributions (% frequency) of respective measurements are provided.

E Particle size distributions (% frequency) of DLS data (respective boxplots see Fig. 6D) were determined for indicated ClpC mutants in presence of ClpP and FITC-casein as indicated. Control reactions showing only ClpP, FITC-casein or ClpP + FITC-casein are provided.

F ATPase deficient DWB variants (E280A/E618A) of ClpC-WT, F436A and D356A were incubated with 2 mM ATP in absence and presence of FITC-casein. Complexes were separated by Superose 6 SEC runs. Elution profiles of ClpC proteins were analyzed by SDS-PAGE and FITC-casein binding was determined by measuring fluorescence of elution fractions. The co-elution of ClpC-F436A or ClpC-D356A with FITC-casein is highlighted.

G Gallery of representative 2D class averages of ClpC D356A in absence and presence of substrate FITC-casein. ClpC-D356A-DWB + FITC-casein complexes were first isolated by Superose 6 SEC runs. Red boxes indicate classes shown in Fig. 6E. Scale bar = 20 nm.

Data information: In (A), SDs (n≥55) are shown as shaded area. In (B-C), SDs are based on at least three independent experiments. In (D), data are represented as described in Figure 2. Standard deviations (n≥52) are shown as shaded area. In (F), SDs are based on at least two independent experiments.

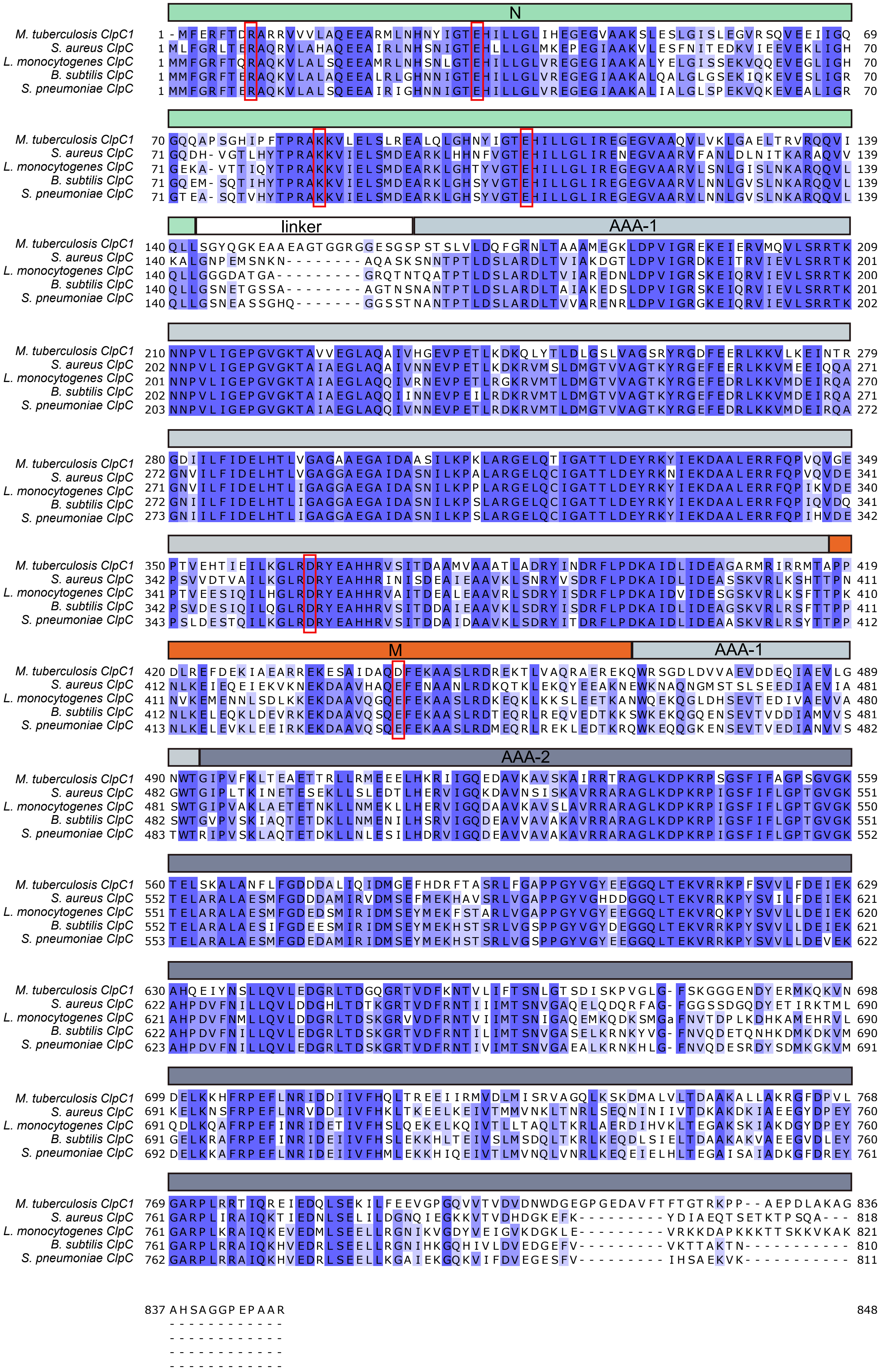
N
linker
AAA-1
M
AAA-2
M. tuberculosis ClpC1
S. aureus ClpC
L. monocytogenes ClpC
B. subtilis ClpC
S. pneumoniae ClpC

Figure S10

Appendix Figure S10 - Multiple sequence alignment of bacterial ClpC proteins.

Sequence alignment of *Mycobacterium tuberculosis* ClpC1, *Staphylococcus aureus* ClpC, *Listeria monocytogenes* ClpC, *Bacillus subtilis* ClpC and *Streptococcus pneumoniae* ClpC. The domain organization is indicated. Similar and identical residues are highlighted in light and dark blue. Residues involved in NTD anchoring within the resting state are framed in red.

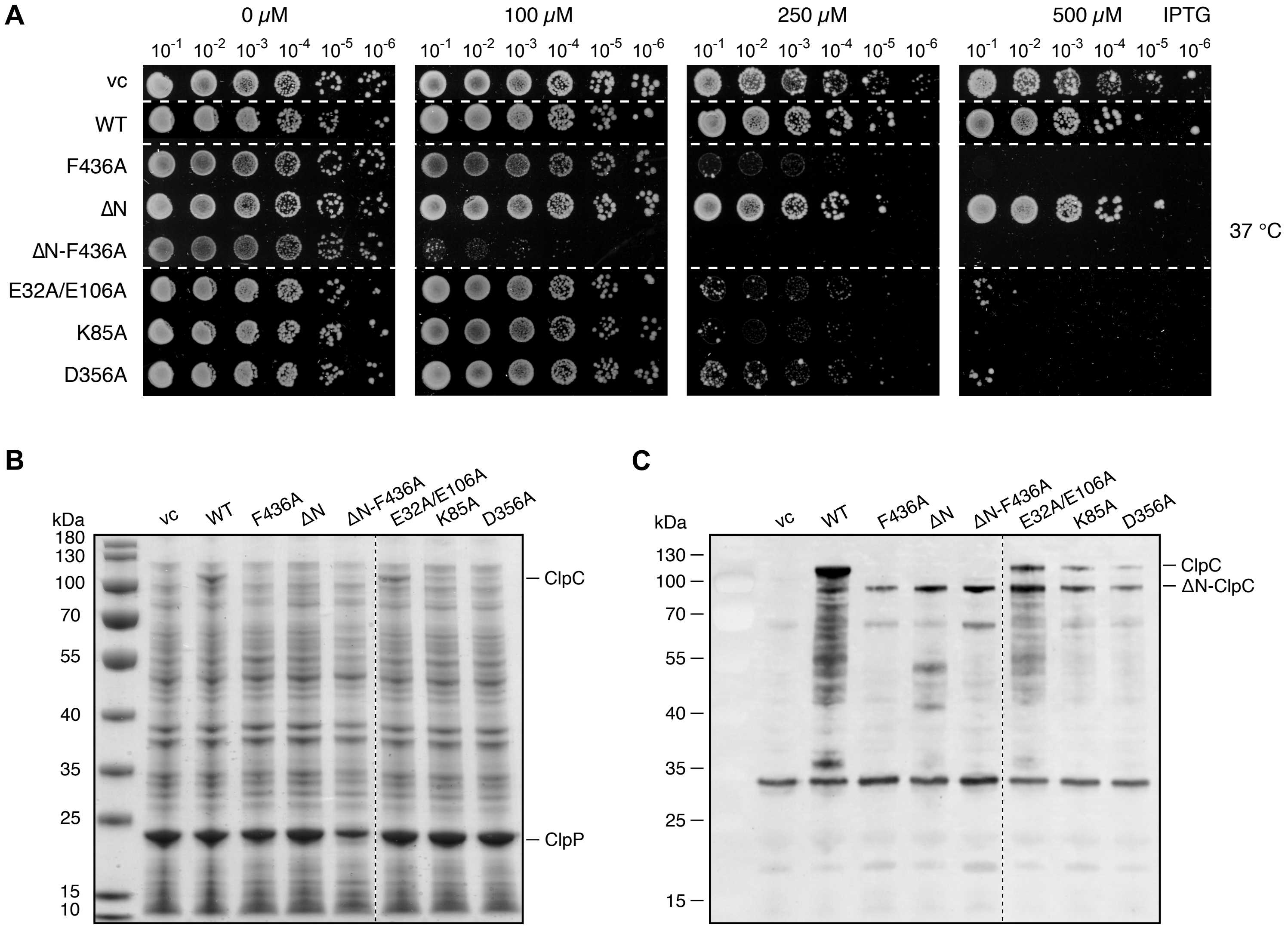
A
0 μM
100 μM
250 μM
500 μM
IPTG
10^-1 10^-2 10^-3 10^-4 10^-5 10^-6
vc
WT
F436A
ΔN
ΔN-F436A
E32A/E106A
K85A
D356A
37 °C
B
kDa
180
130
100
70
55
40
35
25
15
10
vc
WT
F436A
ΔN
ΔN-F436A
E32A/E106A
K85A
D356A
ClpC
ClpP
C
kDa
130
100
70
55
40
35
25
15
vc
WT
F436A
ΔN
ΔN-F436A
E32A/E106A
K85A
D356A
ClpC
ΔN-ClpC

Figure S11

Appendix Figure S11 - Deregulated ClpC mutants create toxicity *in vivo*.

A *E. coli* cells expressing *S. aureus clpP* and harboring indicated plasmid-encoded *clpC* alleles under control of an IPTG-regulatable promoter were grown overnight at 30°C and adjusted to an OD_{600} of 1. Serial dilutions (10^{-1} – 10^{-6}) were spotted on LB plates containing the indicated IPTG concentrations and incubated at 37°C for 24 h.

B, C Production levels of ClpC (WT and mutants) in *E. coli* cells (see Fig. 8). Total cell extracts were prepared and levels of ClpC were determined via SDS-PAGE followed by Coomassie-staining (B). Additionally, ClpC levels were determined by western blot analysis using ClpC-specific antibodies (C).

Data information: In (B-C), a molecular weight marker is provided and bands representing ClpC, ΔN-ClpC and ClpP are indicated. vc = vector control.

A

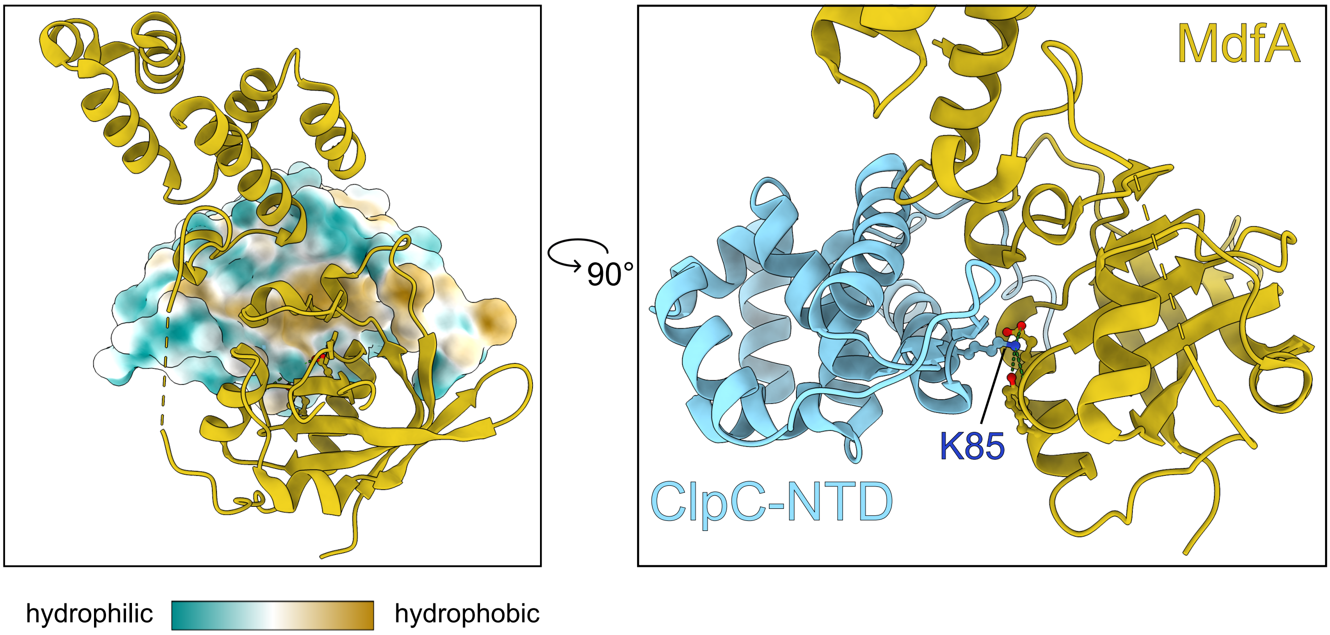

B

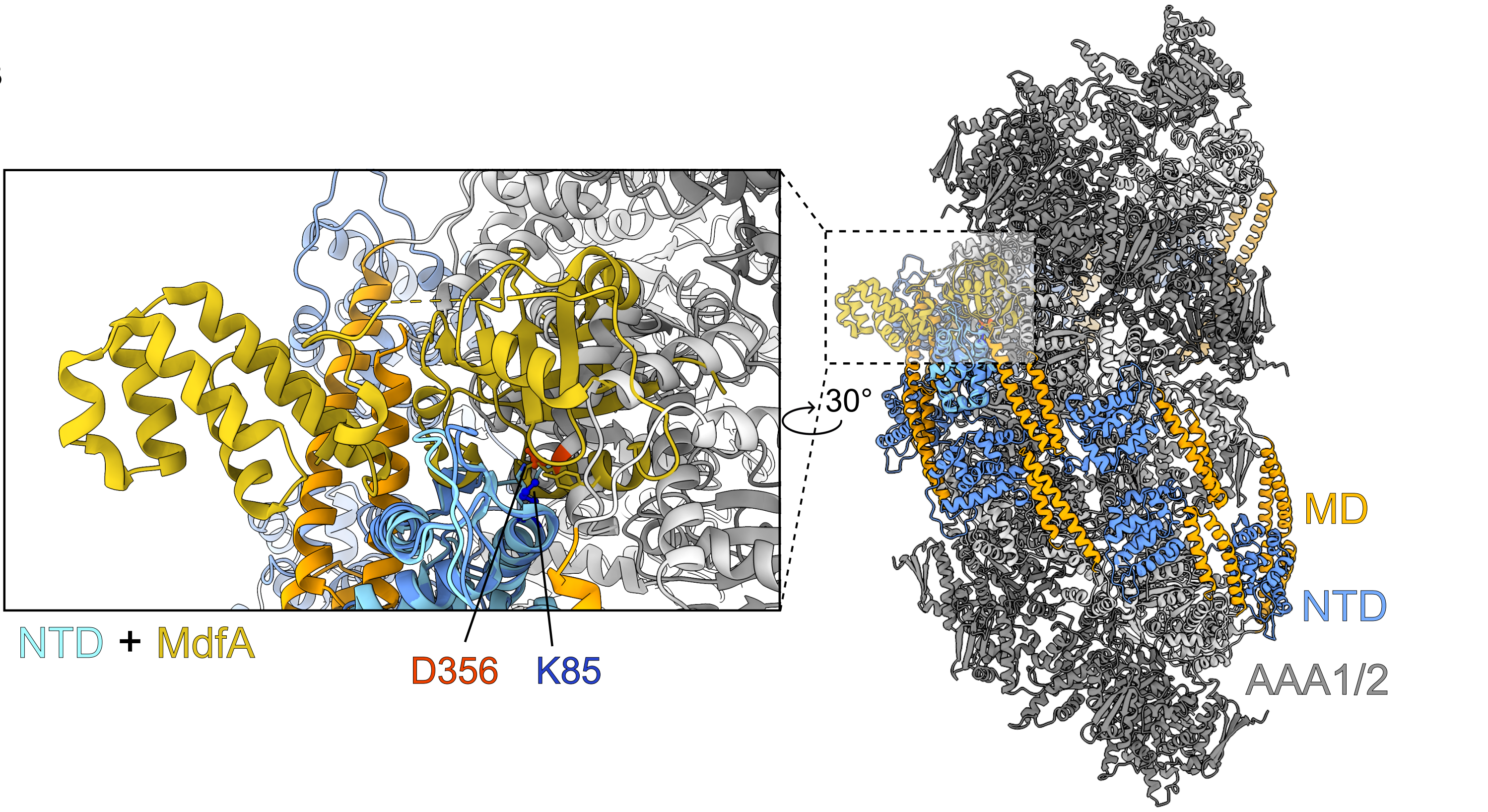

Figure S12

Appendix Figure S12 - Activation of *B. subtilis* ClpC by the sporulation-specific adaptor protein MdfA.

A Co-crystal structure of MdfA bound to the *B. subtilis* ClpC NTD (pdb: 8b3s). MdfA binds to the hydrophobic groove of the NTD and contacts NTD residue K85.

B Superimposition of the MdfA-NTD co-crystal structure onto the *S. aureus* ClpC resting state. MdfA binding is incompatible with resting state formation, triggering ClpC activation.

Appendix Table S1: Cryo-EM data collection, refinement, and validation statistics

**Parameter**	**WT ClpC 14mer ; PDB : 9QCL ; EMDB : 53014**	**WT ClpC 12mer ; PDB : 9QRW ; EMDB : 53324**	**WT ClpC 10mer ; PDB : 9QQR ; EMDB : 53312**
Microscope	Titan Krios G2 microscope		
Detector and energy filter	Gatan K3 + Bioquantum(Ametek)		
Nominal magnification (nominal/calibrated at detector)	105k		
Voltage (kV)	300		
Defocus range (um)	-2.2 to -1.8		
Total electron exposure (or fluence, e-/Å2	40		
Exposure rate (or flux, e-/pixel/s)	15		
Number of frames collected	40		
Pixel size (Å)	0.828		
Energy filter slit width (eV)	20		
Automation software	EPU 3.5		
# Micrographs used	15 057		
Total # of extracted particles	1 301 397		
Total # of refined particles (particles after removing junk)	1 023 583		
# of particles in final map	167 898	179 568	181 306
Resolution of unmasked reconstructions at 0.5 and 0.143 FSC	7.7 Å at 0.4 FSC and 4.4Å at 0.143 FSC	7.6 Å at 0.5 FSC and 4.4Å at 0.143 FSC	4.7 Å at 0.5 FSC and 2.7Å at 0.143 FSC
Local resolution range (Å)	1.53Å to 8.19Å	1.48Å to 7.73Å	1.94Å to 8.86Å
3DFSC Sphericity value	3072 cFSCs / cFAR 0.72	3072 cFSCs / cFAR 0.73	3072 cFSCs / cFAR 0.47
Map sharpening B factor (Å2) / (B factor Range)	88	83.3	83.3
Map sharpening EMReady (any)	yes	yes	no
Map sharpening DeepEMhancer (any)	no	no	no
Atomic modeling refinement package(s)	ChimeraX<br>Coot<br>Phenix<br>Isolde<br>ServalCat_Refmac	ChimeraX<br>Coot<br>Phenix<br>Isolde	ChimeraX<br>Coot<br>Phenix<br>Isolde
CCvolume/CCmask	0.73/0.71	0.75/0.74	0.69/0.67
B factors of protein residues (min/max/mean)	10.87/280.00/115.70	10.87/280.00/116.24	10.87/280.00/115.94
B factors of ligands(min/max/mean)	20.00/20.00/20.00	20.00/20.00/20.00	20.00/20.00/20.00

Bad bond lengths & bad bond angles	0	0	0
Molprobity score	1.26	1.00	1.07
Clashscore	4.9	2.22	2.77
Poor rotamers (%)	0.84	0.11	0.07
Ramachandrans (Favored, Outliers (%))	98.44, 0.00, (allowed is 1,56)	99.03, 0.00	98.98,0.00
CaBLAM outliers (%)	1.62	1.73	1.36

Appendix Table S2: Strains and plasmids used in this study

Strain	Description	Source or reference
E. coli XL1 blue	*recA1 endA1 gyrA96 thi-1 hsdR1 supE44 relA1 lac* [F' *proAB lacI^q ΔM15* Tn10 (Tcr)]	Stratagene
E. coli BL21	*F- ompT lon hsdSB gal dcm λ* (DE3)	Novagen
E. coli ΔclpB::kan	MC4100 Δ*clpB*::Km (Kanamycin resistant)	(Katikaridis *et al*, 2021)
E.coli ΔclpB	MC4100 Δ*clpB* (Kanamycin sensitive)	This study
**Plasmid**	**Description**	**Source or reference**
pDS56	Vector control for IPTG-inducible expression in *E. coli ΔclpB* cells; provides C-terminal His_6-tag	(Carroni *et al*, 2017)
pDS56-*clpC*	Vector for IPTG-inducible expression of ClpC-WT in *E. coli ΔclpB* cells	(Carroni *et al.*, 2017)
pDS56-*clpP*	Vector for IPTG-inducible expression of ClpP in *E. coli ΔclpB* cells	(Carroni *et al.*, 2017)
pDS56-*mecA*	Vector for IPTG-inducible expression of MecA in *E. coli ΔclpB* cells	(Carroni *et al.*, 2017)
pDS56-*clpC*-F436A	Vector for IPTG-inducible expression of ClpC-F436A in *E. coli ΔclpB* cells	(Carroni *et al.*, 2017)
pDS56-*clpC*-ΔN	Vector for IPTG-inducible expression of ClpC-ΔN (2-146) in *E. coli ΔclpB* cells	(Carroni *et al.*, 2017)
pDS56-*clpC*-ΔN-F436A	Vector for IPTG-inducible expression of ClpC-ΔN-F436A in *E. coli ΔclpB* cells	(Carroni *et al.*, 2017)
pDS56-*clpC*-E280A/E618A (DWB)	Vector for IPTG-inducible expression of ClpC-DWB (E280A/E618A) in *E. coli ΔclpB* cells	(Carroni *et al.*, 2017)
pDS56-*clpC*-F436A-DWB	Vector for IPTG-inducible expression of ClpC-F436A-DWB in *E. coli ΔclpB* cells	(Carroni *et al.*, 2017)
pDS56-*clpC*-ΔN-F436A-DWB	Vector for IPTG-inducible expression of ClpC-ΔN-F436A-DWB in *E. coli ΔclpB* cells	(Carroni *et al.*, 2017)
pDS56-*clpC*-E32A	Vector for IPTG-inducible expression of ClpC-E32A in *E. coli ΔclpB* cells	This study
pDS56-*clpC*-E106A	Vector for IPTG-inducible expression of ClpC-E106A in *E. coli ΔclpB* cells	This study
pDS56-*clpC*-E32A-E106A	Vector for IPTG-inducible expression of ClpC-E32A-E106A in *E. coli ΔclpB* cells	This study
pDS56-*clpC*-K85A	Vector for IPTG-inducible expression of ClpC-K85A in *E. coli ΔclpB* cells	This study
pDS56-*clpC*-D356A	Vector for IPTG-inducible expression of ClpC-D356A in *E. coli ΔclpB* cells	This study
pDS56-*clpC*-D356A-F436A	Vector for IPTG-inducible expression of ClpC-D356A-F436A in *E. coli ΔclpB* cells	This study
pDS56-*clpC*-R9A	Vector for IPTG-inducible expression of ClpC-R9A in *E. coli ΔclpB* cells	This study
pDS56-*clpC*-E435A	Vector for IPTG-inducible expression of ClpC-E435A in *E. coli ΔclpB* cells	(Carroni *et al.*, 2017)
pDS56-*clpC*-R122A	Vector for IPTG-inducible expression of ClpC-R122A in *E. coli ΔclpB* cells	This study
pDS56-*clpC*-N462A	Vector for IPTG-inducible expression of ClpC-N462A in *E. coli ΔclpB* cells	This study
pDS56-*clpC*-D356A-DWB	Vector for IPTG-inducible expression of ClpC-D356A-DWB in *E. coli ΔclpB* cells	This study

pET24a-*clpC1*	Vector for IPTG-inducible expression of Mtb ClpC1-WT in *E. coli* BL21 cells	(Taylor *et al*, 2022)
pET24a-*clpC1*-F444S	Vector for IPTG-inducible expression of Mtb ClpC1-F444S in *E. coli* BL21 cells	(Taylor *et al.*, 2022)
pET24a-*clpC1*-K85A	Vector for IPTG-inducible expression of Mtb ClpC1-K85A in *E. coli* BL21 cells	This study
pET24a-*clpC1*-D364A	Vector for IPTG-inducible expression of Mtb ClpC1-D364A in *E. coli* BL21 cells	This study
pDD173-*gfp-SsrA*	Vector for IPTG-inducible expression of GFP-SsrA in *E. coli ∆clpXP* cells	(Carroni *et al.*, 2017)
pDMI.1-*clpP*	Vector for IPTG-inducible co-expression of Sa *clpP* in *E. coli ΔclpB* cells	(Carroni *et al.*, 2017)
placI^q	Vector harboring *lacI* for tighter repression of IPTG-inducible gene expression in *E. coli*	(Katikaridis *et al.*, 2021)

References

Carroni M, Franke KB, Maurer M, Jager J, Hantke I, Gloge F, Linder D, Gremer S, Turgay K, Bukau B *et al* (2017) Regulatory coiled-coil domains promote head-to-head assemblies of AAA+ chaperones essential for tunable activity control. *eLife* 6

Katikaridis P, Romling U, Mogk A (2021) Basic mechanism of the autonomous ClpG disaggregase. *J Biol Chem*: 100460

Taylor G, Frommherz Y, Katikaridis P, Layer D, Sinning I, Carroni M, Weber-Ban E, Mogk A (2022) Antibacterial peptide CyclomarinA creates toxicity by deregulating the Mycobacterium tuberculosis ClpC1-ClpP1P2 protease. *J Biol Chem* 298: 102202
