# Supplementary material for: Allosteric control of the bacterial ClpC/ClpP protease and its hijacking by antibacterial peptides
Source: EMBO J. 2025 Sep 29;44(21):6273–96. doi: 10.1038/s44318-025-00575-1 (PMC12583610; doi:10.1038/s44318-025-00575-1)
Supplement: Supplementary file 3 — Movie EV1 [file 44318_2025_575_MOESM3_ESM.zip › EMBOJ-2025-120881_MovieEV1/Movie EV1_legend.docx]

**Movie EV1**

Building blocks of the ClpC resting states. The overlay of the resting states maps (10-mer in pink, 12-mer in blue and 14-mer in green) highlights the additional dimeric building blocks.
