# Supplementary material for: Allosteric control of the bacterial ClpC/ClpP protease and its hijacking by antibacterial peptides
Source: EMBO J. 2025 Sep 29;44(21):6273–96. doi: 10.1038/s44318-025-00575-1 (PMC12583610; doi:10.1038/s44318-025-00575-1)
Supplement: Supplementary file 4 — Movie EV2 [file 44318_2025_575_MOESM4_ESM.zip › EMBOJ-2025-120881_MovieEV2/Movie EV2_legend.docx]

**Movie EV2**

Motion of the internal NTDs in the ClpC resting state. The cryoDRGN analysis of the 14-mer map shows motion of the internal NTDs (at the back side), contacting different domains.
