# Supplementary material for: Allosteric control of the bacterial ClpC/ClpP protease and its hijacking by antibacterial peptides
Source: EMBO J. 2025 Sep 29;44(21):6273–96. doi: 10.1038/s44318-025-00575-1 (PMC12583610; doi:10.1038/s44318-025-00575-1)
Supplement: Supplementary file 5 — Movie EV3 [file 44318_2025_575_MOESM5_ESM.zip › EMBOJ-2025-120881_MovieEV3/Movie EV3_legend.docx]

**Movie EV3**

ClpC resting state in-and-out movement. Results from the 3D Variability analysis show the different positions (internal or external) of the back side NTDs.
