# Supplementary figures and images for: Allosteric control of the bacterial ClpC/ClpP protease and its hijacking by antibacterial peptides (part 2 of 5)
Source: EMBO J. 2025 Sep 29;44(21):6273–96. doi: 10.1038/s44318-025-00575-1 (PMC12583610; doi:10.1038/s44318-025-00575-1)

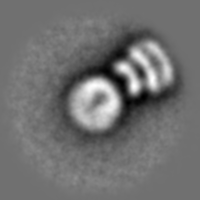

Supplement: Supplementary file 7 — Source data Fig. 2 [file 44318_2025_575_MOESM7_ESM.zip › Figure 2/2C/2d-classes_dN-ClpC-DWB-F436A+ClpP/clsum_10051.tif]

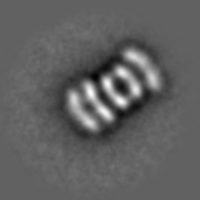

Supplement: Supplementary file 7 — Source data Fig. 2 [file 44318_2025_575_MOESM7_ESM.zip › Figure 2/2C/2d-classes_dN-ClpC-DWB-F436A+ClpP/clsum_10045.tif]

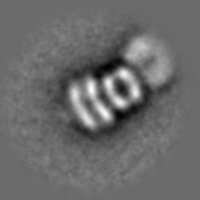

Supplement: Supplementary file 7 — Source data Fig. 2 [file 44318_2025_575_MOESM7_ESM.zip › Figure 2/2C/2d-classes_dN-ClpC-DWB-F436A+ClpP/clsum_10079.tif]

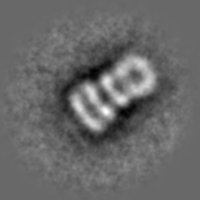

Supplement: Supplementary file 7 — Source data Fig. 2 [file 44318_2025_575_MOESM7_ESM.zip › Figure 2/2C/2d-classes_dN-ClpC-DWB-F436A+ClpP/clsum_10092.tif]

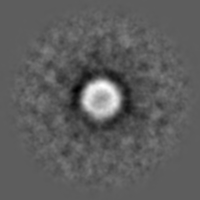

Supplement: Supplementary file 7 — Source data Fig. 2 [file 44318_2025_575_MOESM7_ESM.zip › Figure 2/2C/2d-classes_dN-ClpC-DWB-F436A+ClpP/clsum_10086.tif]

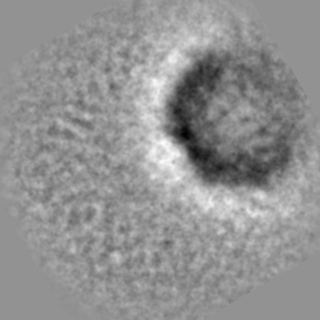

Supplement: Supplementary file 7 — Source data Fig. 2 [file 44318_2025_575_MOESM7_ESM.zip › Figure 2/2C/2d-classes_dN-ClpC-DWB+ClpP/clsum_25-rot30_25.tif]

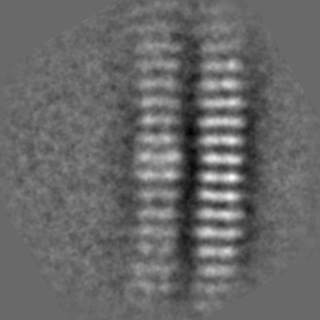

Supplement: Supplementary file 7 — Source data Fig. 2 [file 44318_2025_575_MOESM7_ESM.zip › Figure 2/2C/2d-classes_dN-ClpC-DWB+ClpP/clsum_25-rot30_19.tif]

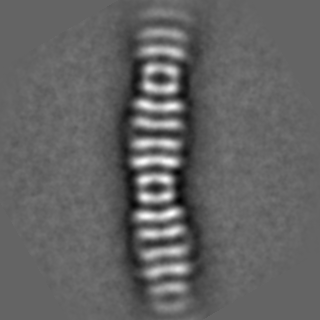

Supplement: Supplementary file 7 — Source data Fig. 2 [file 44318_2025_575_MOESM7_ESM.zip › Figure 2/2C/2d-classes_dN-ClpC-DWB+ClpP/clsum_25-rot30_18.tif]

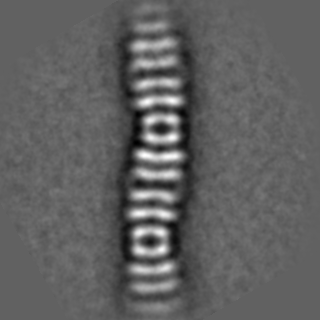

Supplement: Supplementary file 7 — Source data Fig. 2 [file 44318_2025_575_MOESM7_ESM.zip › Figure 2/2C/2d-classes_dN-ClpC-DWB+ClpP/clsum_25-rot30_24.tif]

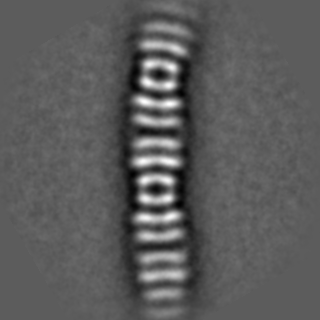

Supplement: Supplementary file 7 — Source data Fig. 2 [file 44318_2025_575_MOESM7_ESM.zip › Figure 2/2C/2d-classes_dN-ClpC-DWB+ClpP/clsum_25-rot30_23.tif]

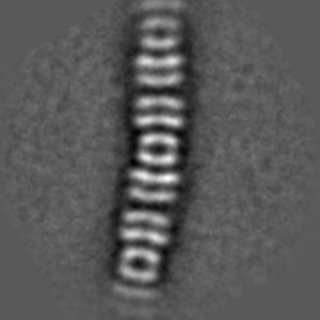

Supplement: Supplementary file 7 — Source data Fig. 2 [file 44318_2025_575_MOESM7_ESM.zip › Figure 2/2C/2d-classes_dN-ClpC-DWB+ClpP/clsum_25-rot30_22.tif]

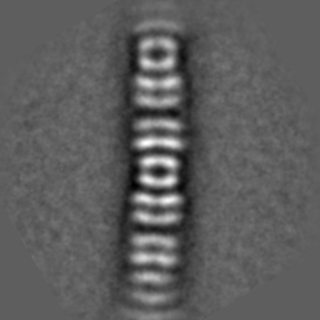

Supplement: Supplementary file 7 — Source data Fig. 2 [file 44318_2025_575_MOESM7_ESM.zip › Figure 2/2C/2d-classes_dN-ClpC-DWB+ClpP/clsum_25-rot30_08.tif]

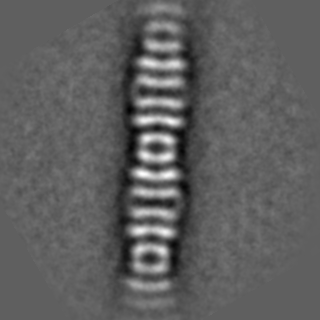

Supplement: Supplementary file 7 — Source data Fig. 2 [file 44318_2025_575_MOESM7_ESM.zip › Figure 2/2C/2d-classes_dN-ClpC-DWB+ClpP/clsum_25-rot30_20.tif]

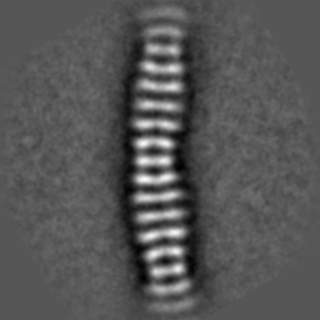

Supplement: Supplementary file 7 — Source data Fig. 2 [file 44318_2025_575_MOESM7_ESM.zip › Figure 2/2C/2d-classes_dN-ClpC-DWB+ClpP/clsum_25-rot30_21.tif]

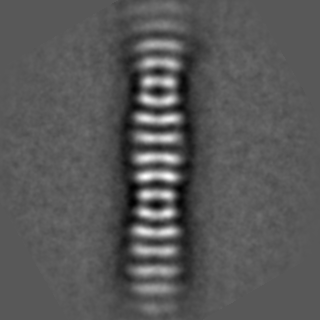

Supplement: Supplementary file 7 — Source data Fig. 2 [file 44318_2025_575_MOESM7_ESM.zip › Figure 2/2C/2d-classes_dN-ClpC-DWB+ClpP/clsum_25-rot30_09.tif]

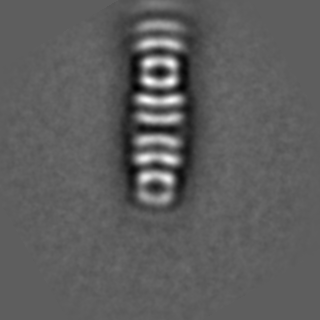

Supplement: Supplementary file 7 — Source data Fig. 2 [file 44318_2025_575_MOESM7_ESM.zip › Figure 2/2C/2d-classes_dN-ClpC-DWB+ClpP/clsum_25-rot30_04.tif]

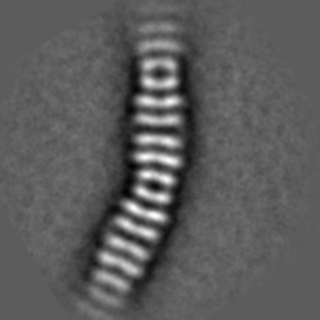

Supplement: Supplementary file 7 — Source data Fig. 2 [file 44318_2025_575_MOESM7_ESM.zip › Figure 2/2C/2d-classes_dN-ClpC-DWB+ClpP/clsum_25-rot30_10.tif]

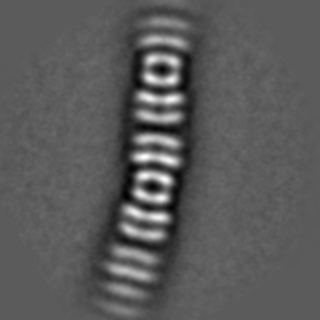

Supplement: Supplementary file 7 — Source data Fig. 2 [file 44318_2025_575_MOESM7_ESM.zip › Figure 2/2C/2d-classes_dN-ClpC-DWB+ClpP/clsum_25-rot30_11.tif]

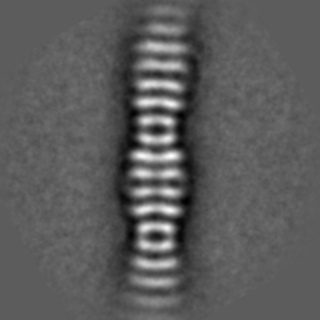

Supplement: Supplementary file 7 — Source data Fig. 2 [file 44318_2025_575_MOESM7_ESM.zip › Figure 2/2C/2d-classes_dN-ClpC-DWB+ClpP/clsum_25-rot30_05.tif]

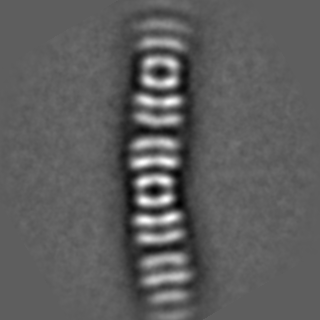

Supplement: Supplementary file 7 — Source data Fig. 2 [file 44318_2025_575_MOESM7_ESM.zip › Figure 2/2C/2d-classes_dN-ClpC-DWB+ClpP/clsum_25-rot30_13.tif]

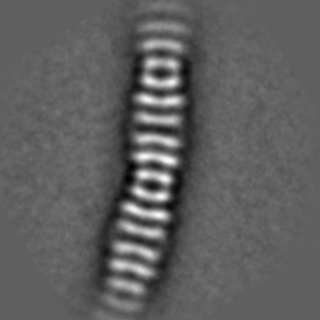

Supplement: Supplementary file 7 — Source data Fig. 2 [file 44318_2025_575_MOESM7_ESM.zip › Figure 2/2C/2d-classes_dN-ClpC-DWB+ClpP/clsum_25-rot30_07.tif]

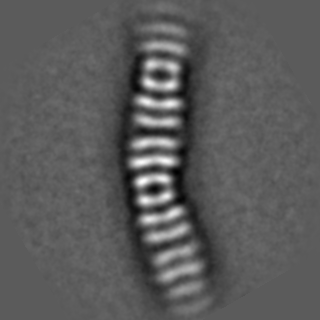

Supplement: Supplementary file 7 — Source data Fig. 2 [file 44318_2025_575_MOESM7_ESM.zip › Figure 2/2C/2d-classes_dN-ClpC-DWB+ClpP/clsum_25-rot30_06.tif]

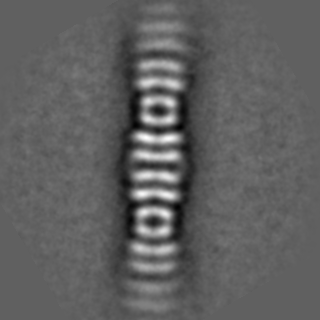

Supplement: Supplementary file 7 — Source data Fig. 2 [file 44318_2025_575_MOESM7_ESM.zip › Figure 2/2C/2d-classes_dN-ClpC-DWB+ClpP/clsum_25-rot30_12.tif]

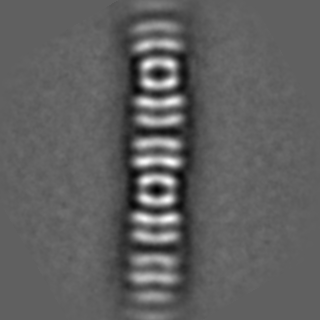

Supplement: Supplementary file 7 — Source data Fig. 2 [file 44318_2025_575_MOESM7_ESM.zip › Figure 2/2C/2d-classes_dN-ClpC-DWB+ClpP/clsum_25-rot30_16.tif]

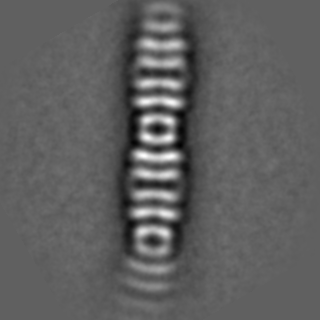

Supplement: Supplementary file 7 — Source data Fig. 2 [file 44318_2025_575_MOESM7_ESM.zip › Figure 2/2C/2d-classes_dN-ClpC-DWB+ClpP/clsum_25-rot30_02.tif]

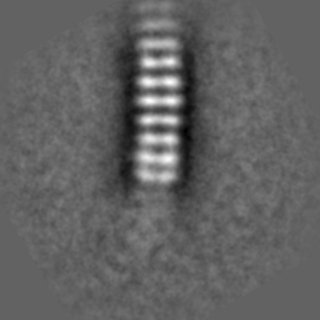

Supplement: Supplementary file 7 — Source data Fig. 2 [file 44318_2025_575_MOESM7_ESM.zip › Figure 2/2C/2d-classes_dN-ClpC-DWB+ClpP/clsum_25-rot30_03.tif]

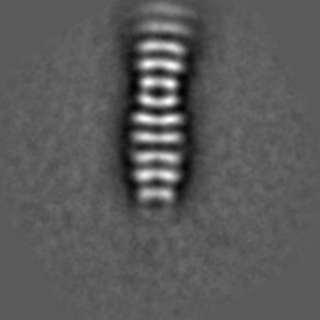

Supplement: Supplementary file 7 — Source data Fig. 2 [file 44318_2025_575_MOESM7_ESM.zip › Figure 2/2C/2d-classes_dN-ClpC-DWB+ClpP/clsum_25-rot30_17.tif]

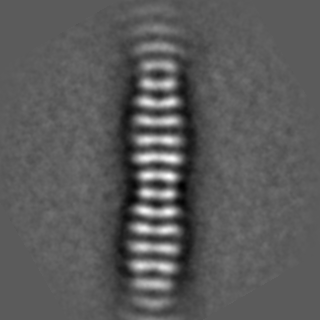

Supplement: Supplementary file 7 — Source data Fig. 2 [file 44318_2025_575_MOESM7_ESM.zip › Figure 2/2C/2d-classes_dN-ClpC-DWB+ClpP/clsum_25-rot30_01.tif]

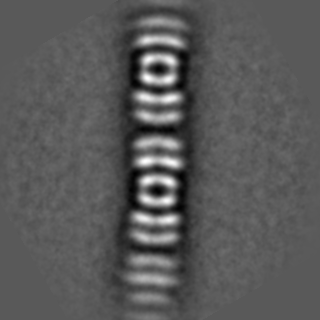

Supplement: Supplementary file 7 — Source data Fig. 2 [file 44318_2025_575_MOESM7_ESM.zip › Figure 2/2C/2d-classes_dN-ClpC-DWB+ClpP/clsum_25-rot30_15.tif]

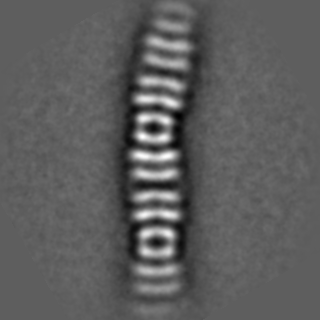

Supplement: Supplementary file 7 — Source data Fig. 2 [file 44318_2025_575_MOESM7_ESM.zip › Figure 2/2C/2d-classes_dN-ClpC-DWB+ClpP/clsum_25-rot30_14.tif]

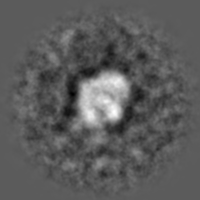

Supplement: Supplementary file 7 — Source data Fig. 2 [file 44318_2025_575_MOESM7_ESM.zip › Figure 2/2C/2d-classes_ClpC-DWB/clsum_100-091.tif]

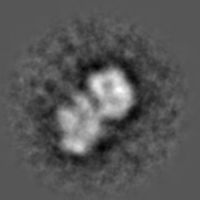

Supplement: Supplementary file 7 — Source data Fig. 2 [file 44318_2025_575_MOESM7_ESM.zip › Figure 2/2C/2d-classes_ClpC-DWB/clsum_100-085.tif]

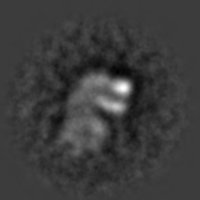

Supplement: Supplementary file 7 — Source data Fig. 2 [file 44318_2025_575_MOESM7_ESM.zip › Figure 2/2C/2d-classes_ClpC-DWB/clsum_100-052.tif]

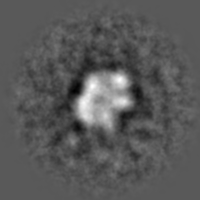

Supplement: Supplementary file 7 — Source data Fig. 2 [file 44318_2025_575_MOESM7_ESM.zip › Figure 2/2C/2d-classes_ClpC-DWB/clsum_100-046.tif]

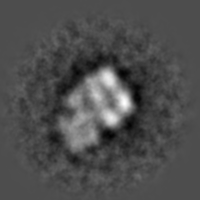

Supplement: Supplementary file 7 — Source data Fig. 2 [file 44318_2025_575_MOESM7_ESM.zip › Figure 2/2C/2d-classes_ClpC-DWB/clsum_100-047.tif]

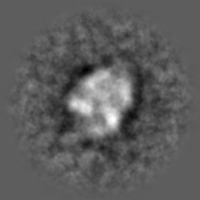

Supplement: Supplementary file 7 — Source data Fig. 2 [file 44318_2025_575_MOESM7_ESM.zip › Figure 2/2C/2d-classes_ClpC-DWB/clsum_100-053.tif]

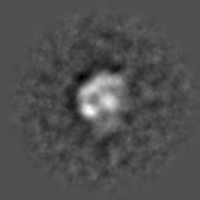

Supplement: Supplementary file 7 — Source data Fig. 2 [file 44318_2025_575_MOESM7_ESM.zip › Figure 2/2C/2d-classes_ClpC-DWB/clsum_100-084.tif]

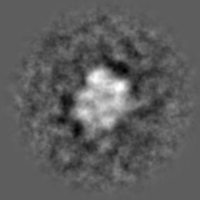

Supplement: Supplementary file 7 — Source data Fig. 2 [file 44318_2025_575_MOESM7_ESM.zip › Figure 2/2C/2d-classes_ClpC-DWB/clsum_100-090.tif]

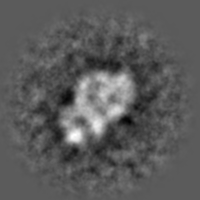

Supplement: Supplementary file 7 — Source data Fig. 2 [file 44318_2025_575_MOESM7_ESM.zip › Figure 2/2C/2d-classes_ClpC-DWB/clsum_100-086.tif]

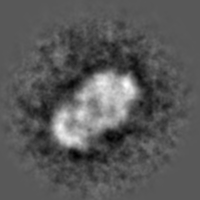

Supplement: Supplementary file 7 — Source data Fig. 2 [file 44318_2025_575_MOESM7_ESM.zip › Figure 2/2C/2d-classes_ClpC-DWB/clsum_100-092.tif]

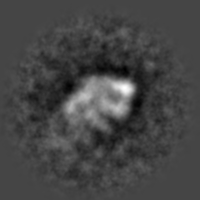

Supplement: Supplementary file 7 — Source data Fig. 2 [file 44318_2025_575_MOESM7_ESM.zip › Figure 2/2C/2d-classes_ClpC-DWB/clsum_100-045.tif]

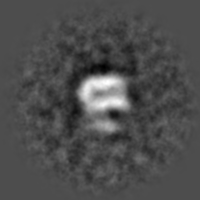

Supplement: Supplementary file 7 — Source data Fig. 2 [file 44318_2025_575_MOESM7_ESM.zip › Figure 2/2C/2d-classes_ClpC-DWB/clsum_100-051.tif]

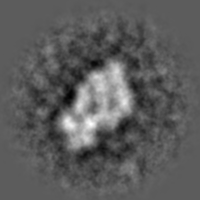

Supplement: Supplementary file 7 — Source data Fig. 2 [file 44318_2025_575_MOESM7_ESM.zip › Figure 2/2C/2d-classes_ClpC-DWB/clsum_100-079.tif]

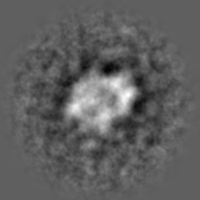

Supplement: Supplementary file 7 — Source data Fig. 2 [file 44318_2025_575_MOESM7_ESM.zip › Figure 2/2C/2d-classes_ClpC-DWB/clsum_100-078.tif]

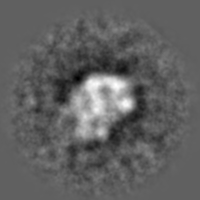

Supplement: Supplementary file 7 — Source data Fig. 2 [file 44318_2025_575_MOESM7_ESM.zip › Figure 2/2C/2d-classes_ClpC-DWB/clsum_100-050.tif]

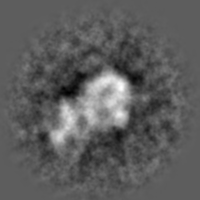

Supplement: Supplementary file 7 — Source data Fig. 2 [file 44318_2025_575_MOESM7_ESM.zip › Figure 2/2C/2d-classes_ClpC-DWB/clsum_100-044.tif]

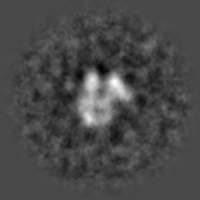

Supplement: Supplementary file 7 — Source data Fig. 2 [file 44318_2025_575_MOESM7_ESM.zip › Figure 2/2C/2d-classes_ClpC-DWB/clsum_100-093.tif]

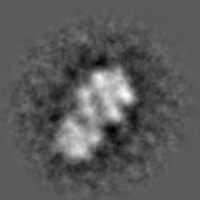

Supplement: Supplementary file 7 — Source data Fig. 2 [file 44318_2025_575_MOESM7_ESM.zip › Figure 2/2C/2d-classes_ClpC-DWB/clsum_100-087.tif]

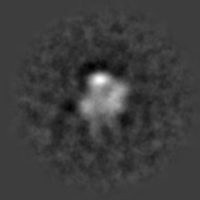

Supplement: Supplementary file 7 — Source data Fig. 2 [file 44318_2025_575_MOESM7_ESM.zip › Figure 2/2C/2d-classes_ClpC-DWB/clsum_100-083.tif]

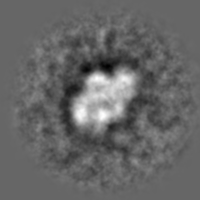

Supplement: Supplementary file 7 — Source data Fig. 2 [file 44318_2025_575_MOESM7_ESM.zip › Figure 2/2C/2d-classes_ClpC-DWB/clsum_100-097.tif]

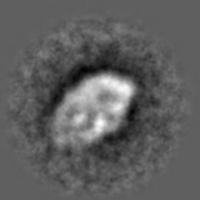

Supplement: Supplementary file 7 — Source data Fig. 2 [file 44318_2025_575_MOESM7_ESM.zip › Figure 2/2C/2d-classes_ClpC-DWB/clsum_100-068.tif]

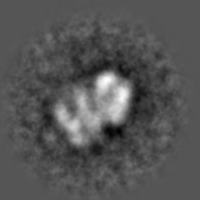

Supplement: Supplementary file 7 — Source data Fig. 2 [file 44318_2025_575_MOESM7_ESM.zip › Figure 2/2C/2d-classes_ClpC-DWB/clsum_100-040.tif]

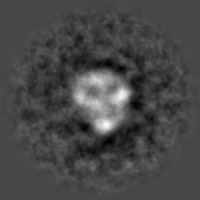

Supplement: Supplementary file 7 — Source data Fig. 2 [file 44318_2025_575_MOESM7_ESM.zip › Figure 2/2C/2d-classes_ClpC-DWB/clsum_100-054.tif]

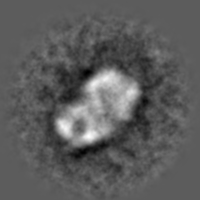

Supplement: Supplementary file 7 — Source data Fig. 2 [file 44318_2025_575_MOESM7_ESM.zip › Figure 2/2C/2d-classes_ClpC-DWB/clsum_100-055.tif]

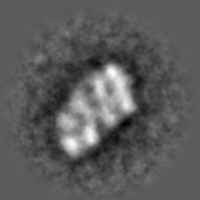

Supplement: Supplementary file 7 — Source data Fig. 2 [file 44318_2025_575_MOESM7_ESM.zip › Figure 2/2C/2d-classes_ClpC-DWB/clsum_100-041.tif]

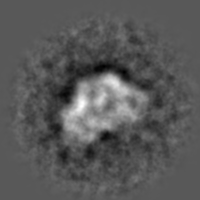

Supplement: Supplementary file 7 — Source data Fig. 2 [file 44318_2025_575_MOESM7_ESM.zip › Figure 2/2C/2d-classes_ClpC-DWB/clsum_100-069.tif]

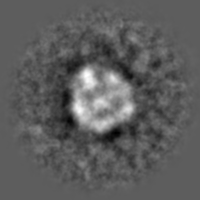

Supplement: Supplementary file 7 — Source data Fig. 2 [file 44318_2025_575_MOESM7_ESM.zip › Figure 2/2C/2d-classes_ClpC-DWB/clsum_100-096.tif]

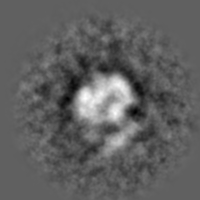

Supplement: Supplementary file 7 — Source data Fig. 2 [file 44318_2025_575_MOESM7_ESM.zip › Figure 2/2C/2d-classes_ClpC-DWB/clsum_100-082.tif]

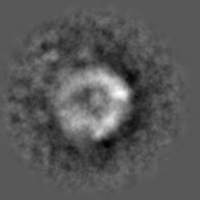

Supplement: Supplementary file 7 — Source data Fig. 2 [file 44318_2025_575_MOESM7_ESM.zip › Figure 2/2C/2d-classes_ClpC-DWB/clsum_100-094.tif]

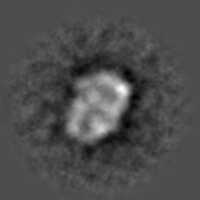

Supplement: Supplementary file 7 — Source data Fig. 2 [file 44318_2025_575_MOESM7_ESM.zip › Figure 2/2C/2d-classes_ClpC-DWB/clsum_100-080.tif]

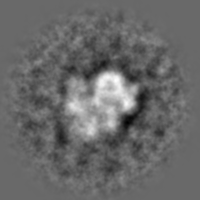

Supplement: Supplementary file 7 — Source data Fig. 2 [file 44318_2025_575_MOESM7_ESM.zip › Figure 2/2C/2d-classes_ClpC-DWB/clsum_100-057.tif]

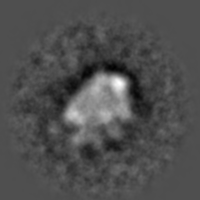

Supplement: Supplementary file 7 — Source data Fig. 2 [file 44318_2025_575_MOESM7_ESM.zip › Figure 2/2C/2d-classes_ClpC-DWB/clsum_100-043.tif]

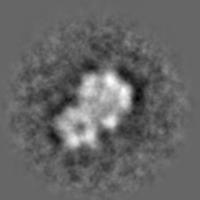

Supplement: Supplementary file 7 — Source data Fig. 2 [file 44318_2025_575_MOESM7_ESM.zip › Figure 2/2C/2d-classes_ClpC-DWB/clsum_100-042.tif]

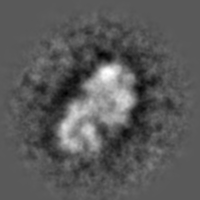

Supplement: Supplementary file 7 — Source data Fig. 2 [file 44318_2025_575_MOESM7_ESM.zip › Figure 2/2C/2d-classes_ClpC-DWB/clsum_100-056.tif]

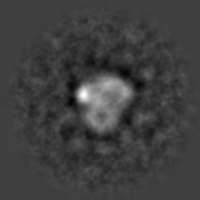

Supplement: Supplementary file 7 — Source data Fig. 2 [file 44318_2025_575_MOESM7_ESM.zip › Figure 2/2C/2d-classes_ClpC-DWB/clsum_100-081.tif]

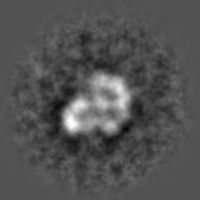

Supplement: Supplementary file 7 — Source data Fig. 2 [file 44318_2025_575_MOESM7_ESM.zip › Figure 2/2C/2d-classes_ClpC-DWB/clsum_100-095.tif]

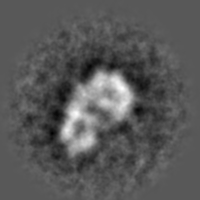

Supplement: Supplementary file 7 — Source data Fig. 2 [file 44318_2025_575_MOESM7_ESM.zip › Figure 2/2C/2d-classes_ClpC-DWB/clsum_100-031.tif]

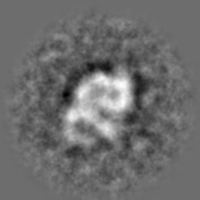

Supplement: Supplementary file 7 — Source data Fig. 2 [file 44318_2025_575_MOESM7_ESM.zip › Figure 2/2C/2d-classes_ClpC-DWB/clsum_100-025.tif]

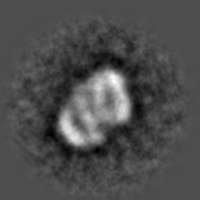

Supplement: Supplementary file 7 — Source data Fig. 2 [file 44318_2025_575_MOESM7_ESM.zip › Figure 2/2C/2d-classes_ClpC-DWB/clsum_100-019.tif]

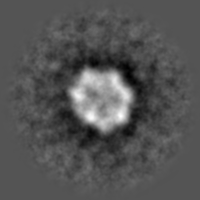

Supplement: Supplementary file 7 — Source data Fig. 2 [file 44318_2025_575_MOESM7_ESM.zip › Figure 2/2C/2d-classes_ClpC-DWB/clsum_100-018.tif]

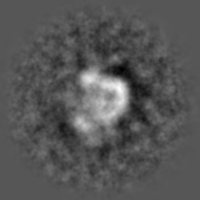

Supplement: Supplementary file 7 — Source data Fig. 2 [file 44318_2025_575_MOESM7_ESM.zip › Figure 2/2C/2d-classes_ClpC-DWB/clsum_100-024.tif]

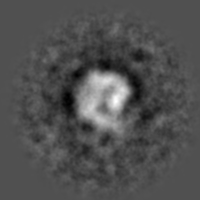

Supplement: Supplementary file 7 — Source data Fig. 2 [file 44318_2025_575_MOESM7_ESM.zip › Figure 2/2C/2d-classes_ClpC-DWB/clsum_100-030.tif]

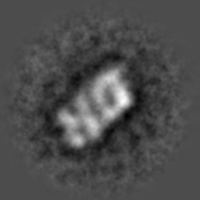

Supplement: Supplementary file 7 — Source data Fig. 2 [file 44318_2025_575_MOESM7_ESM.zip › Figure 2/2C/2d-classes_ClpC-DWB/clsum_100-026.tif]

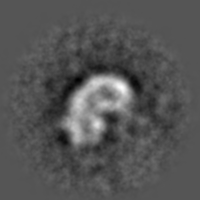

Supplement: Supplementary file 7 — Source data Fig. 2 [file 44318_2025_575_MOESM7_ESM.zip › Figure 2/2C/2d-classes_ClpC-DWB/clsum_100-032.tif]

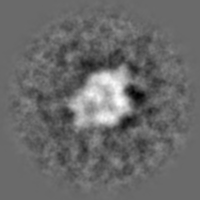

Supplement: Supplementary file 7 — Source data Fig. 2 [file 44318_2025_575_MOESM7_ESM.zip › Figure 2/2C/2d-classes_ClpC-DWB/clsum_100-033.tif]

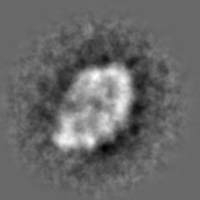

Supplement: Supplementary file 7 — Source data Fig. 2 [file 44318_2025_575_MOESM7_ESM.zip › Figure 2/2C/2d-classes_ClpC-DWB/clsum_100-027.tif]

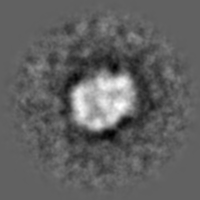

Supplement: Supplementary file 7 — Source data Fig. 2 [file 44318_2025_575_MOESM7_ESM.zip › Figure 2/2C/2d-classes_ClpC-DWB/clsum_100-023.tif]

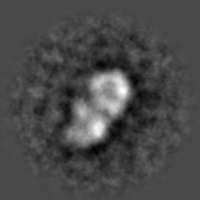

Supplement: Supplementary file 7 — Source data Fig. 2 [file 44318_2025_575_MOESM7_ESM.zip › Figure 2/2C/2d-classes_ClpC-DWB/clsum_100-037.tif]

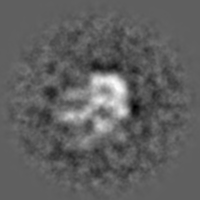

Supplement: Supplementary file 7 — Source data Fig. 2 [file 44318_2025_575_MOESM7_ESM.zip › Figure 2/2C/2d-classes_ClpC-DWB/clsum_100-036.tif]

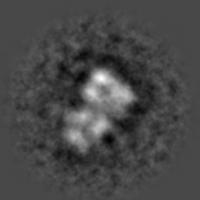

Supplement: Supplementary file 7 — Source data Fig. 2 [file 44318_2025_575_MOESM7_ESM.zip › Figure 2/2C/2d-classes_ClpC-DWB/clsum_100-022.tif]

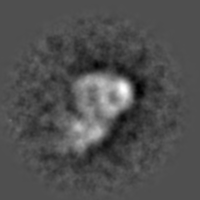

Supplement: Supplementary file 7 — Source data Fig. 2 [file 44318_2025_575_MOESM7_ESM.zip › Figure 2/2C/2d-classes_ClpC-DWB/clsum_100-008.tif]

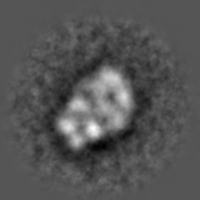

Supplement: Supplementary file 7 — Source data Fig. 2 [file 44318_2025_575_MOESM7_ESM.zip › Figure 2/2C/2d-classes_ClpC-DWB/clsum_100-034.tif]

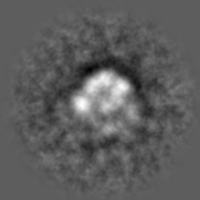

Supplement: Supplementary file 7 — Source data Fig. 2 [file 44318_2025_575_MOESM7_ESM.zip › Figure 2/2C/2d-classes_ClpC-DWB/clsum_100-020.tif]

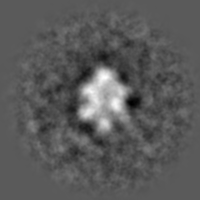

Supplement: Supplementary file 7 — Source data Fig. 2 [file 44318_2025_575_MOESM7_ESM.zip › Figure 2/2C/2d-classes_ClpC-DWB/clsum_100-021.tif]

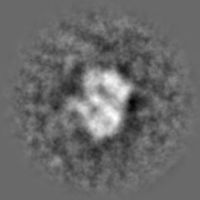

Supplement: Supplementary file 7 — Source data Fig. 2 [file 44318_2025_575_MOESM7_ESM.zip › Figure 2/2C/2d-classes_ClpC-DWB/clsum_100-035.tif]

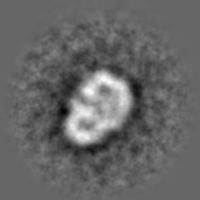

Supplement: Supplementary file 7 — Source data Fig. 2 [file 44318_2025_575_MOESM7_ESM.zip › Figure 2/2C/2d-classes_ClpC-DWB/clsum_100-009.tif]

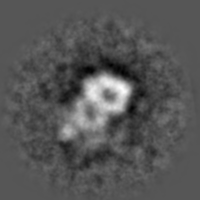

Supplement: Supplementary file 7 — Source data Fig. 2 [file 44318_2025_575_MOESM7_ESM.zip › Figure 2/2C/2d-classes_ClpC-DWB/clsum_100-010.tif]

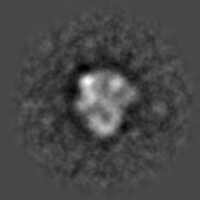

Supplement: Supplementary file 7 — Source data Fig. 2 [file 44318_2025_575_MOESM7_ESM.zip › Figure 2/2C/2d-classes_ClpC-DWB/clsum_100-004.tif]

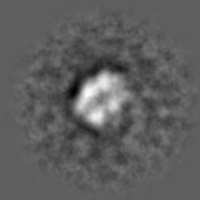

Supplement: Supplementary file 7 — Source data Fig. 2 [file 44318_2025_575_MOESM7_ESM.zip › Figure 2/2C/2d-classes_ClpC-DWB/clsum_100-038.tif]

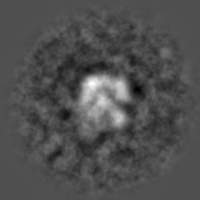

Supplement: Supplementary file 7 — Source data Fig. 2 [file 44318_2025_575_MOESM7_ESM.zip › Figure 2/2C/2d-classes_ClpC-DWB/clsum_100-039.tif]

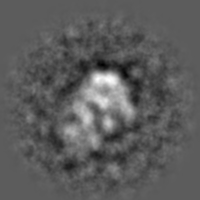

Supplement: Supplementary file 7 — Source data Fig. 2 [file 44318_2025_575_MOESM7_ESM.zip › Figure 2/2C/2d-classes_ClpC-DWB/clsum_100-005.tif]

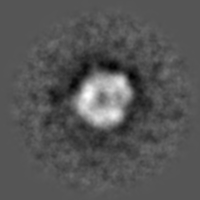

Supplement: Supplementary file 7 — Source data Fig. 2 [file 44318_2025_575_MOESM7_ESM.zip › Figure 2/2C/2d-classes_ClpC-DWB/clsum_100-011.tif]

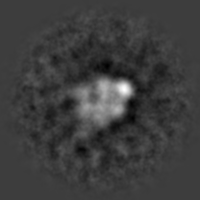

Supplement: Supplementary file 7 — Source data Fig. 2 [file 44318_2025_575_MOESM7_ESM.zip › Figure 2/2C/2d-classes_ClpC-DWB/clsum_100-007.tif]

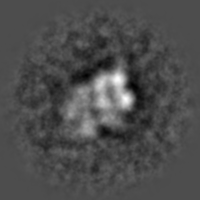

Supplement: Supplementary file 7 — Source data Fig. 2 [file 44318_2025_575_MOESM7_ESM.zip › Figure 2/2C/2d-classes_ClpC-DWB/clsum_100-013.tif]

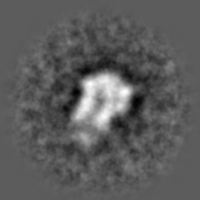

Supplement: Supplementary file 7 — Source data Fig. 2 [file 44318_2025_575_MOESM7_ESM.zip › Figure 2/2C/2d-classes_ClpC-DWB/clsum_100-012.tif]

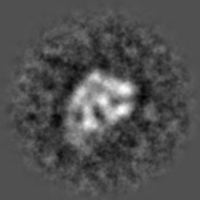

Supplement: Supplementary file 7 — Source data Fig. 2 [file 44318_2025_575_MOESM7_ESM.zip › Figure 2/2C/2d-classes_ClpC-DWB/clsum_100-006.tif]

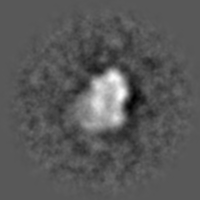

Supplement: Supplementary file 7 — Source data Fig. 2 [file 44318_2025_575_MOESM7_ESM.zip › Figure 2/2C/2d-classes_ClpC-DWB/clsum_100-002.tif]

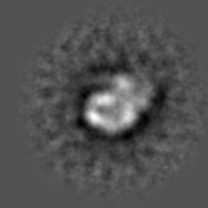

Supplement: Supplementary file 9 — Source data Fig. 4 [file 44318_2025_575_MOESM9_ESM.zip › Figure 4/4C/2d-classes_ClpC-WT/clsum_50-049.tif]

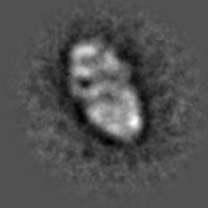

Supplement: Supplementary file 9 — Source data Fig. 4 [file 44318_2025_575_MOESM9_ESM.zip › Figure 4/4C/2d-classes_ClpC-WT/clsum_50-048.tif]

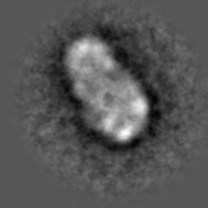

Supplement: Supplementary file 9 — Source data Fig. 4 [file 44318_2025_575_MOESM9_ESM.zip › Figure 4/4C/2d-classes_ClpC-WT/clsum_50-002.tif]
