# Supplementary figures and images for: Allosteric control of the bacterial ClpC/ClpP protease and its hijacking by antibacterial peptides (part 1 of 5)
Source: EMBO J. 2025 Sep 29;44(21):6273–96. doi: 10.1038/s44318-025-00575-1 (PMC12583610; doi:10.1038/s44318-025-00575-1)

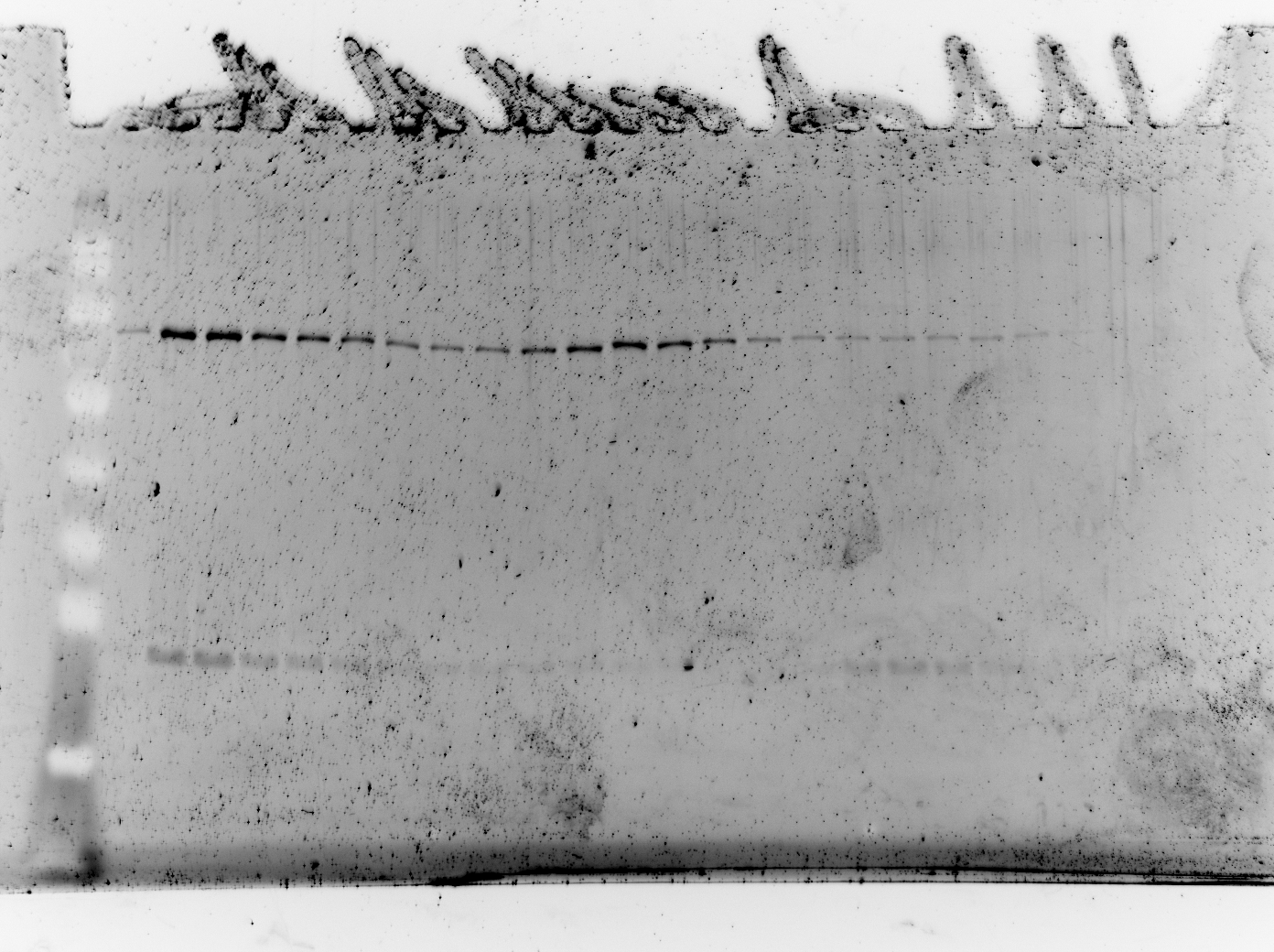

Supplement: Supplementary file 7 — Source data Fig. 2 [file 44318_2025_575_MOESM7_ESM.zip › Figure 2/2B/dN-ClpC-DWB-ClpP.tif]

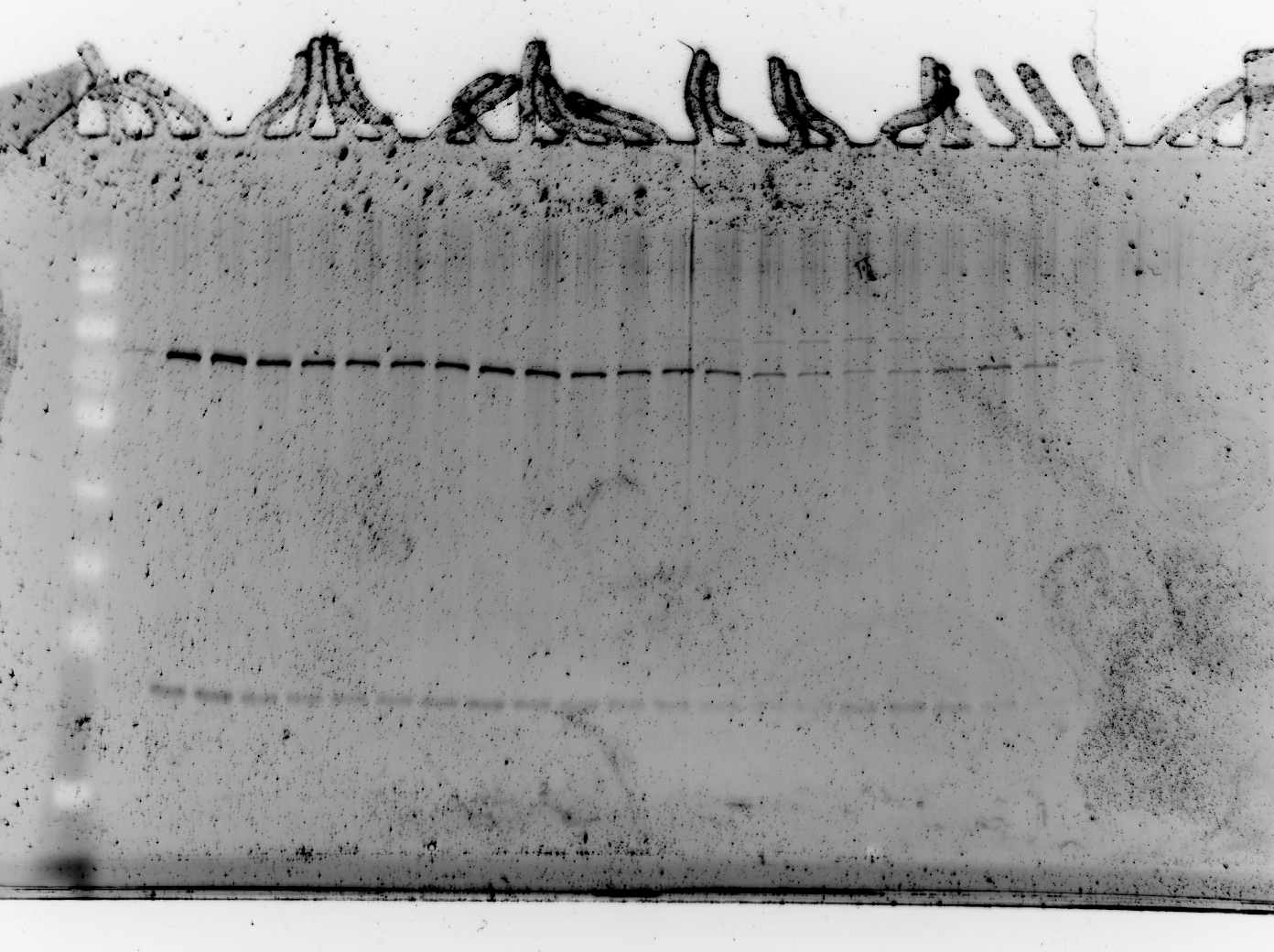

Supplement: Supplementary file 7 — Source data Fig. 2 [file 44318_2025_575_MOESM7_ESM.zip › Figure 2/2B/dN-ClpC-DWB-F436A-ClpP.tif]

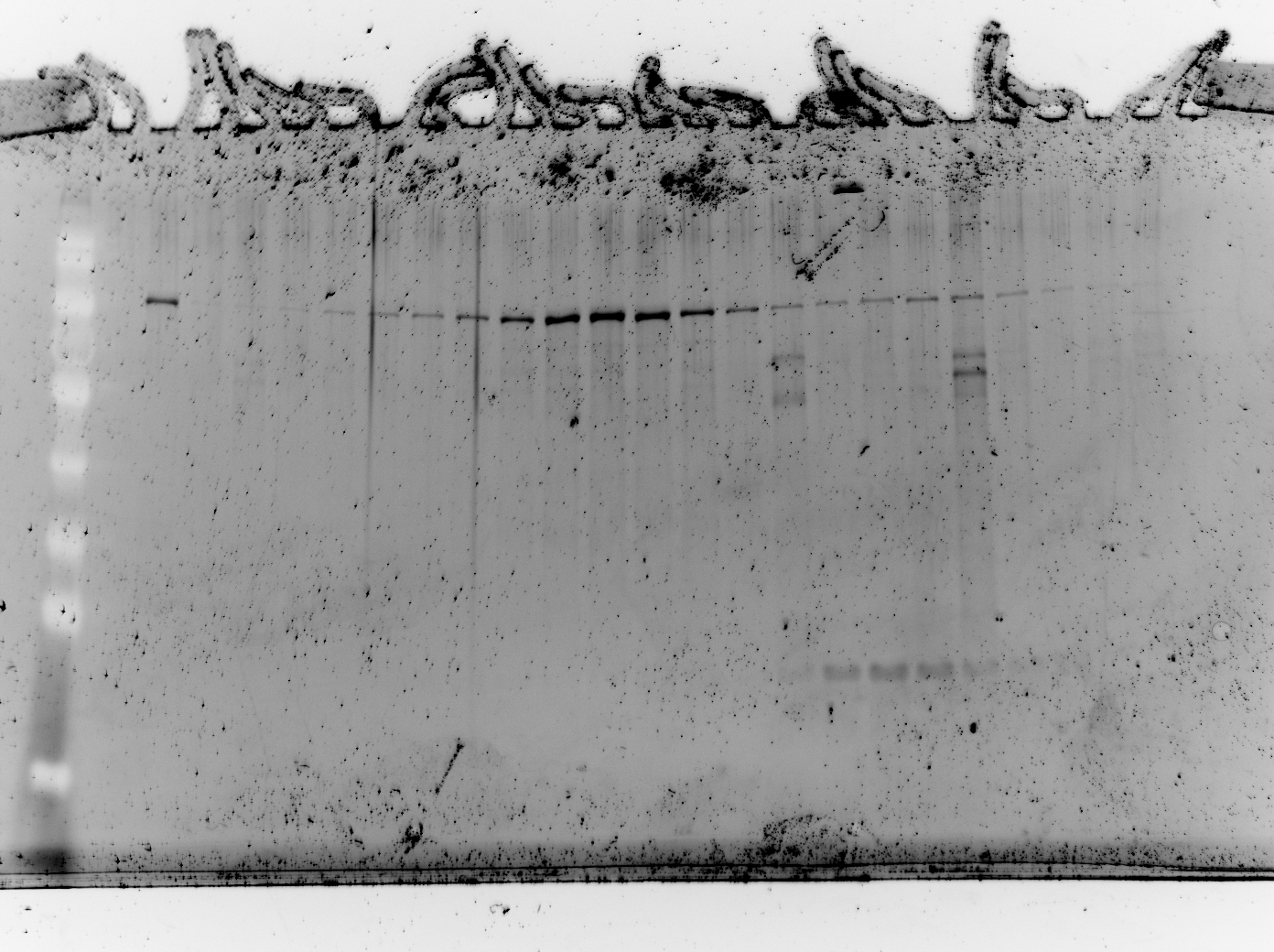

Supplement: Supplementary file 7 — Source data Fig. 2 [file 44318_2025_575_MOESM7_ESM.zip › Figure 2/2B/ClpC-DWB+ClpP.tif]

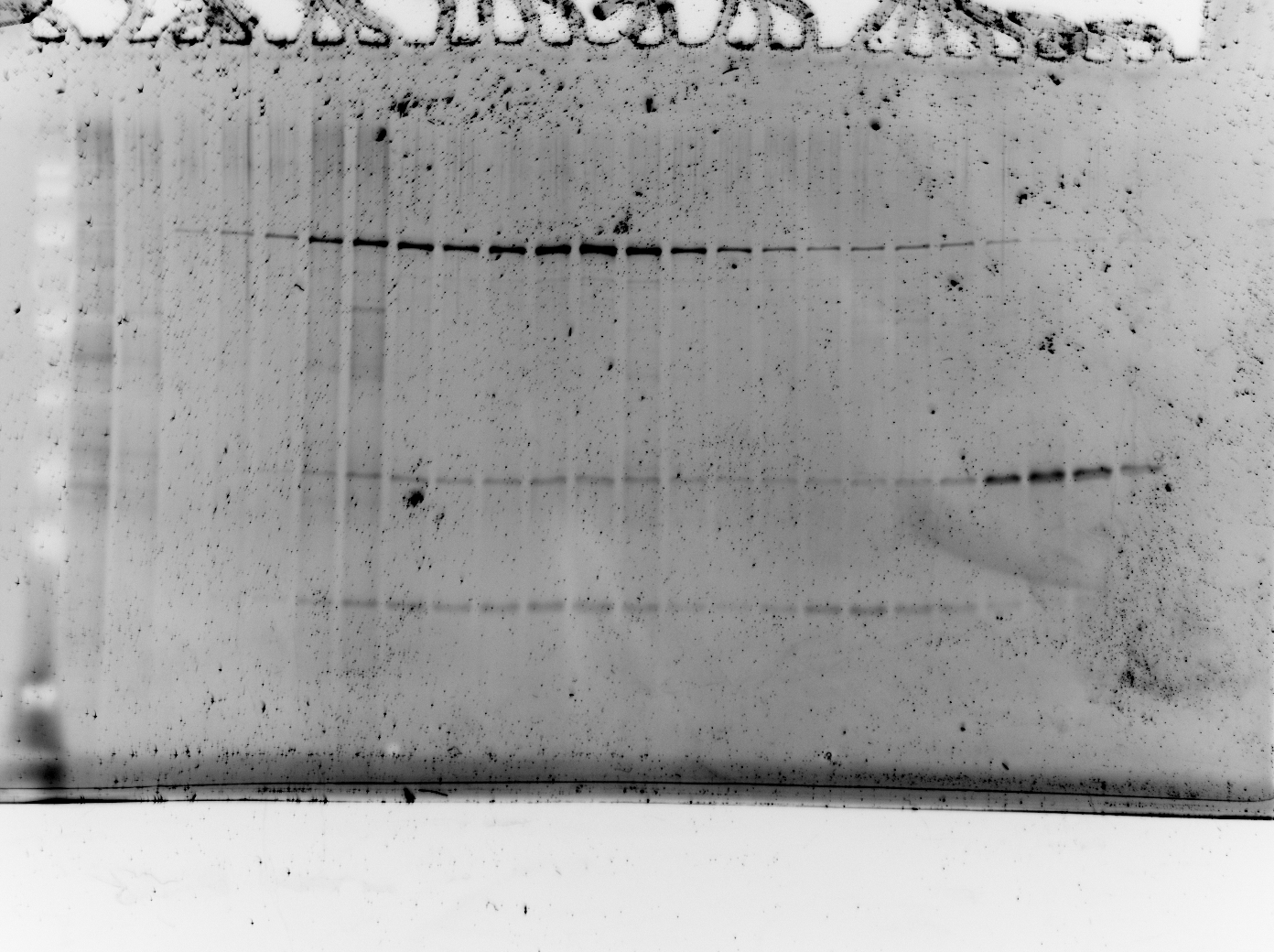

Supplement: Supplementary file 7 — Source data Fig. 2 [file 44318_2025_575_MOESM7_ESM.zip › Figure 2/2B/ClpC-DWB+MecA+ClpP.tif]

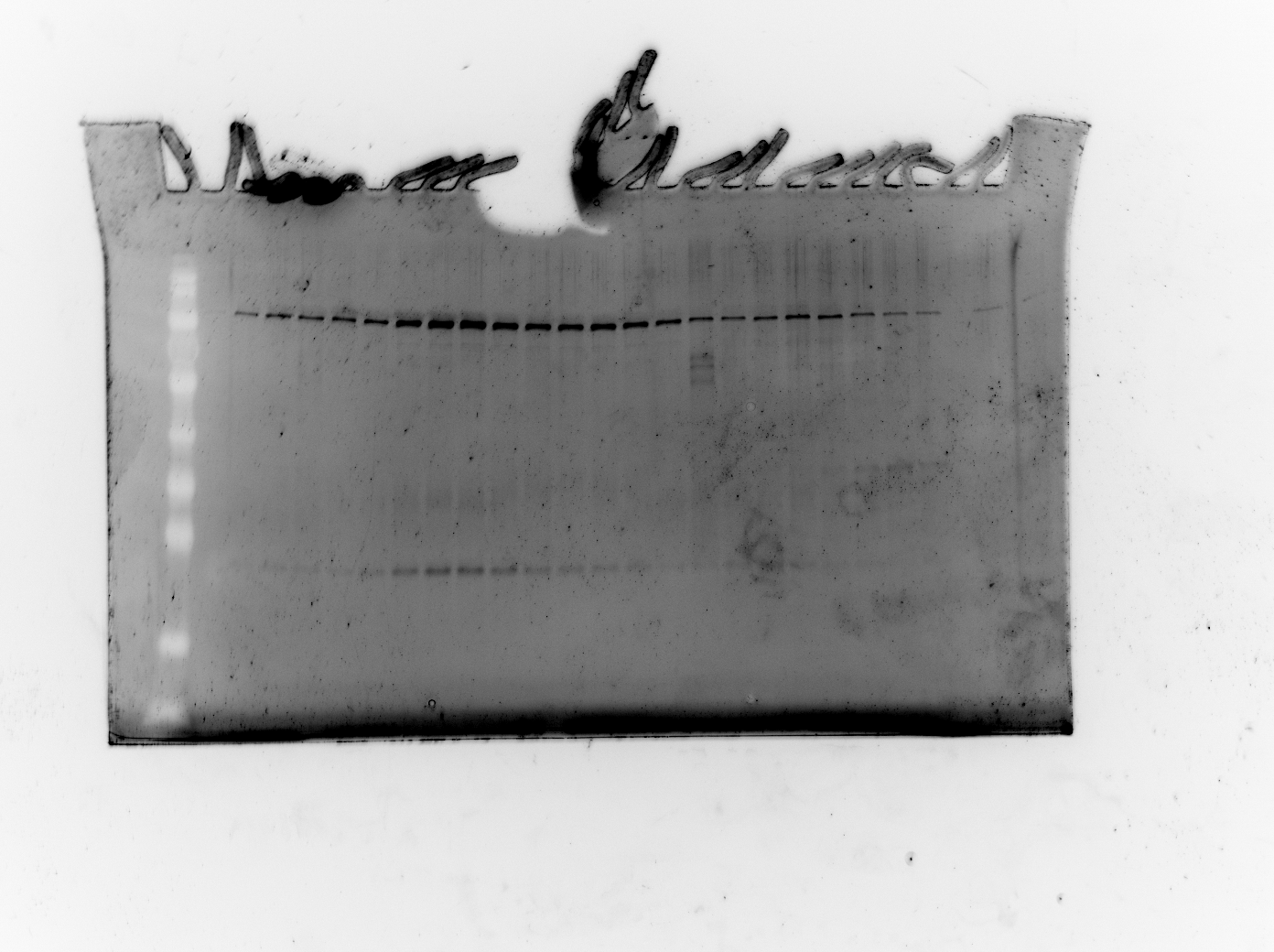

Supplement: Supplementary file 7 — Source data Fig. 2 [file 44318_2025_575_MOESM7_ESM.zip › Figure 2/2B/ClpC-DWB-F436A-ClpP.tif]

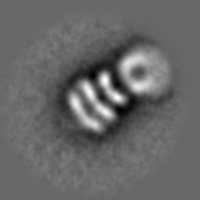

Supplement: Supplementary file 7 — Source data Fig. 2 [file 44318_2025_575_MOESM7_ESM.zip › Figure 2/2C/2d-classes_dN-ClpC-DWB-F436A+ClpP/clsum_10060.tif]

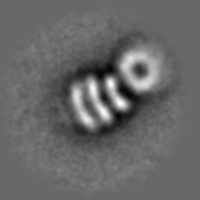

Supplement: Supplementary file 7 — Source data Fig. 2 [file 44318_2025_575_MOESM7_ESM.zip › Figure 2/2C/2d-classes_dN-ClpC-DWB-F436A+ClpP/clsum_10074.tif]

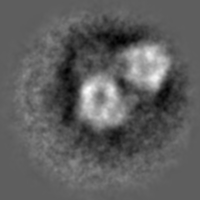

Supplement: Supplementary file 7 — Source data Fig. 2 [file 44318_2025_575_MOESM7_ESM.zip › Figure 2/2C/2d-classes_dN-ClpC-DWB-F436A+ClpP/clsum_10048.tif]

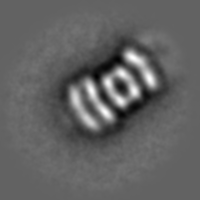

Supplement: Supplementary file 7 — Source data Fig. 2 [file 44318_2025_575_MOESM7_ESM.zip › Figure 2/2C/2d-classes_dN-ClpC-DWB-F436A+ClpP/clsum_10049.tif]

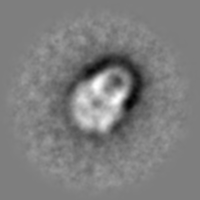

Supplement: Supplementary file 7 — Source data Fig. 2 [file 44318_2025_575_MOESM7_ESM.zip › Figure 2/2C/2d-classes_dN-ClpC-DWB-F436A+ClpP/clsum_10075.tif]

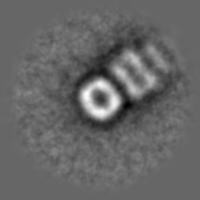

Supplement: Supplementary file 7 — Source data Fig. 2 [file 44318_2025_575_MOESM7_ESM.zip › Figure 2/2C/2d-classes_dN-ClpC-DWB-F436A+ClpP/clsum_10061.tif]

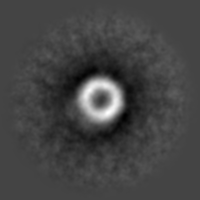

Supplement: Supplementary file 7 — Source data Fig. 2 [file 44318_2025_575_MOESM7_ESM.zip › Figure 2/2C/2d-classes_dN-ClpC-DWB-F436A+ClpP/clsum_10088.tif]

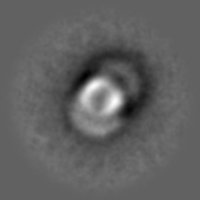

Supplement: Supplementary file 7 — Source data Fig. 2 [file 44318_2025_575_MOESM7_ESM.zip › Figure 2/2C/2d-classes_dN-ClpC-DWB-F436A+ClpP/clsum_10077.tif]

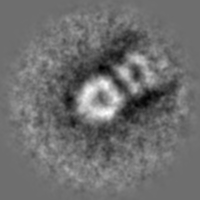

Supplement: Supplementary file 7 — Source data Fig. 2 [file 44318_2025_575_MOESM7_ESM.zip › Figure 2/2C/2d-classes_dN-ClpC-DWB-F436A+ClpP/clsum_10063.tif]

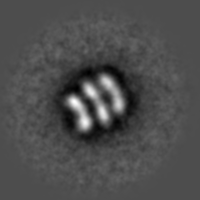

Supplement: Supplementary file 7 — Source data Fig. 2 [file 44318_2025_575_MOESM7_ESM.zip › Figure 2/2C/2d-classes_dN-ClpC-DWB-F436A+ClpP/clsum_10062.tif]

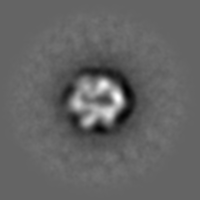

Supplement: Supplementary file 7 — Source data Fig. 2 [file 44318_2025_575_MOESM7_ESM.zip › Figure 2/2C/2d-classes_dN-ClpC-DWB-F436A+ClpP/clsum_10076.tif]

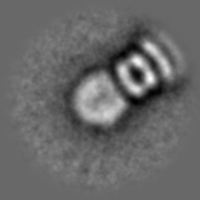

Supplement: Supplementary file 7 — Source data Fig. 2 [file 44318_2025_575_MOESM7_ESM.zip › Figure 2/2C/2d-classes_dN-ClpC-DWB-F436A+ClpP/clsum_10089.tif]

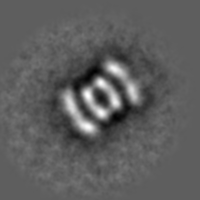

Supplement: Supplementary file 7 — Source data Fig. 2 [file 44318_2025_575_MOESM7_ESM.zip › Figure 2/2C/2d-classes_dN-ClpC-DWB-F436A+ClpP/clsum_100100.tif]

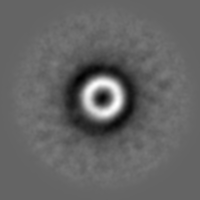

Supplement: Supplementary file 7 — Source data Fig. 2 [file 44318_2025_575_MOESM7_ESM.zip › Figure 2/2C/2d-classes_dN-ClpC-DWB-F436A+ClpP/clsum_10099.tif]

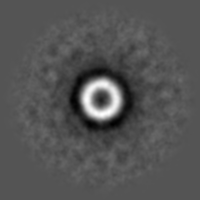

Supplement: Supplementary file 7 — Source data Fig. 2 [file 44318_2025_575_MOESM7_ESM.zip › Figure 2/2C/2d-classes_dN-ClpC-DWB-F436A+ClpP/clsum_10072.tif]

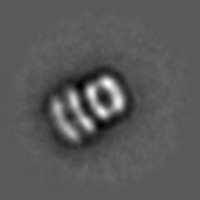

Supplement: Supplementary file 7 — Source data Fig. 2 [file 44318_2025_575_MOESM7_ESM.zip › Figure 2/2C/2d-classes_dN-ClpC-DWB-F436A+ClpP/clsum_10066.tif]

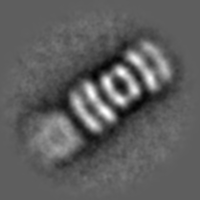

Supplement: Supplementary file 7 — Source data Fig. 2 [file 44318_2025_575_MOESM7_ESM.zip › Figure 2/2C/2d-classes_dN-ClpC-DWB-F436A+ClpP/clsum_10067.tif]

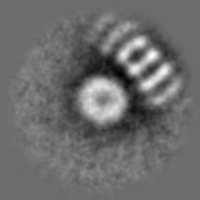

Supplement: Supplementary file 7 — Source data Fig. 2 [file 44318_2025_575_MOESM7_ESM.zip › Figure 2/2C/2d-classes_dN-ClpC-DWB-F436A+ClpP/clsum_10073.tif]

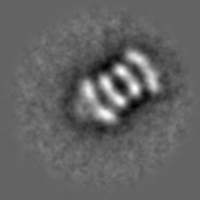

Supplement: Supplementary file 7 — Source data Fig. 2 [file 44318_2025_575_MOESM7_ESM.zip › Figure 2/2C/2d-classes_dN-ClpC-DWB-F436A+ClpP/clsum_10098.tif]

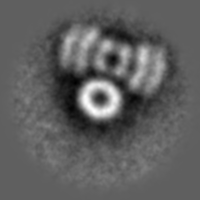

Supplement: Supplementary file 7 — Source data Fig. 2 [file 44318_2025_575_MOESM7_ESM.zip › Figure 2/2C/2d-classes_dN-ClpC-DWB-F436A+ClpP/clsum_10059.tif]

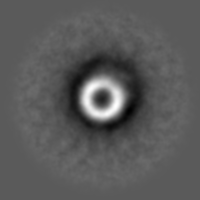

Supplement: Supplementary file 7 — Source data Fig. 2 [file 44318_2025_575_MOESM7_ESM.zip › Figure 2/2C/2d-classes_dN-ClpC-DWB-F436A+ClpP/clsum_10065.tif]

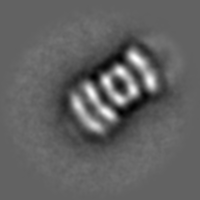

Supplement: Supplementary file 7 — Source data Fig. 2 [file 44318_2025_575_MOESM7_ESM.zip › Figure 2/2C/2d-classes_dN-ClpC-DWB-F436A+ClpP/clsum_10071.tif]

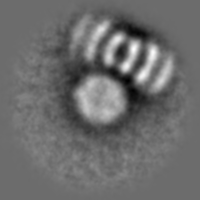

Supplement: Supplementary file 7 — Source data Fig. 2 [file 44318_2025_575_MOESM7_ESM.zip › Figure 2/2C/2d-classes_dN-ClpC-DWB-F436A+ClpP/clsum_10070.tif]

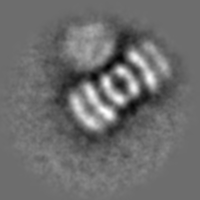

Supplement: Supplementary file 7 — Source data Fig. 2 [file 44318_2025_575_MOESM7_ESM.zip › Figure 2/2C/2d-classes_dN-ClpC-DWB-F436A+ClpP/clsum_10064.tif]

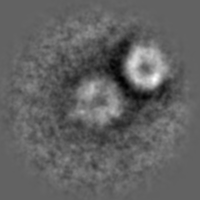

Supplement: Supplementary file 7 — Source data Fig. 2 [file 44318_2025_575_MOESM7_ESM.zip › Figure 2/2C/2d-classes_dN-ClpC-DWB-F436A+ClpP/clsum_10058.tif]

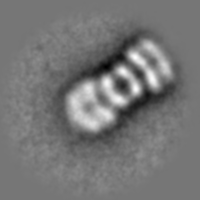

Supplement: Supplementary file 7 — Source data Fig. 2 [file 44318_2025_575_MOESM7_ESM.zip › Figure 2/2C/2d-classes_dN-ClpC-DWB-F436A+ClpP/clsum_10017.tif]

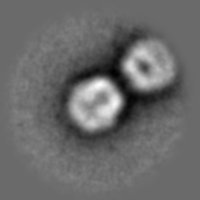

Supplement: Supplementary file 7 — Source data Fig. 2 [file 44318_2025_575_MOESM7_ESM.zip › Figure 2/2C/2d-classes_dN-ClpC-DWB-F436A+ClpP/clsum_10016.tif]

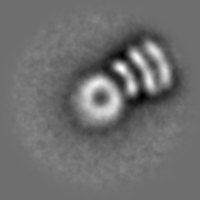

Supplement: Supplementary file 7 — Source data Fig. 2 [file 44318_2025_575_MOESM7_ESM.zip › Figure 2/2C/2d-classes_dN-ClpC-DWB-F436A+ClpP/clsum_10014.tif]

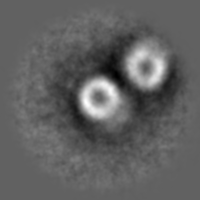

Supplement: Supplementary file 7 — Source data Fig. 2 [file 44318_2025_575_MOESM7_ESM.zip › Figure 2/2C/2d-classes_dN-ClpC-DWB-F436A+ClpP/clsum_10028.tif]

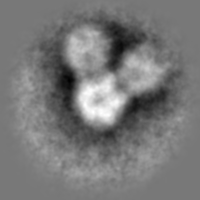

Supplement: Supplementary file 7 — Source data Fig. 2 [file 44318_2025_575_MOESM7_ESM.zip › Figure 2/2C/2d-classes_dN-ClpC-DWB-F436A+ClpP/clsum_10029.tif]

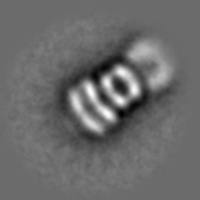

Supplement: Supplementary file 7 — Source data Fig. 2 [file 44318_2025_575_MOESM7_ESM.zip › Figure 2/2C/2d-classes_dN-ClpC-DWB-F436A+ClpP/clsum_10015.tif]

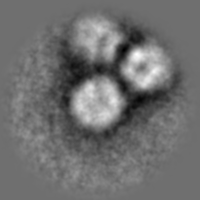

Supplement: Supplementary file 7 — Source data Fig. 2 [file 44318_2025_575_MOESM7_ESM.zip › Figure 2/2C/2d-classes_dN-ClpC-DWB-F436A+ClpP/clsum_10039.tif]

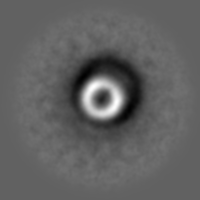

Supplement: Supplementary file 7 — Source data Fig. 2 [file 44318_2025_575_MOESM7_ESM.zip › Figure 2/2C/2d-classes_dN-ClpC-DWB-F436A+ClpP/clsum_10011.tif]

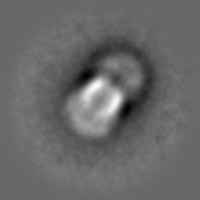

Supplement: Supplementary file 7 — Source data Fig. 2 [file 44318_2025_575_MOESM7_ESM.zip › Figure 2/2C/2d-classes_dN-ClpC-DWB-F436A+ClpP/clsum_1009.tif]

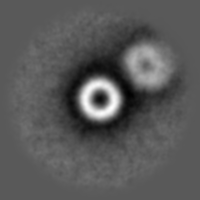

Supplement: Supplementary file 7 — Source data Fig. 2 [file 44318_2025_575_MOESM7_ESM.zip › Figure 2/2C/2d-classes_dN-ClpC-DWB-F436A+ClpP/clsum_1008.tif]

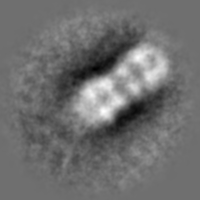

Supplement: Supplementary file 7 — Source data Fig. 2 [file 44318_2025_575_MOESM7_ESM.zip › Figure 2/2C/2d-classes_dN-ClpC-DWB-F436A+ClpP/clsum_10010.tif]

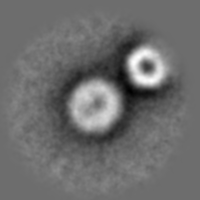

Supplement: Supplementary file 7 — Source data Fig. 2 [file 44318_2025_575_MOESM7_ESM.zip › Figure 2/2C/2d-classes_dN-ClpC-DWB-F436A+ClpP/clsum_10038.tif]

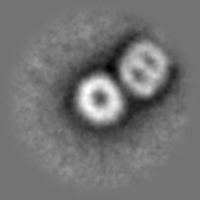

Supplement: Supplementary file 7 — Source data Fig. 2 [file 44318_2025_575_MOESM7_ESM.zip › Figure 2/2C/2d-classes_dN-ClpC-DWB-F436A+ClpP/clsum_10012.tif]

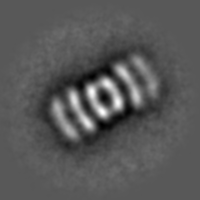

Supplement: Supplementary file 7 — Source data Fig. 2 [file 44318_2025_575_MOESM7_ESM.zip › Figure 2/2C/2d-classes_dN-ClpC-DWB-F436A+ClpP/clsum_10013.tif]

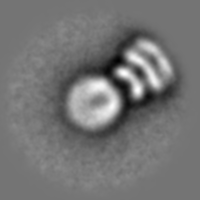

Supplement: Supplementary file 7 — Source data Fig. 2 [file 44318_2025_575_MOESM7_ESM.zip › Figure 2/2C/2d-classes_dN-ClpC-DWB-F436A+ClpP/clsum_10022.tif]

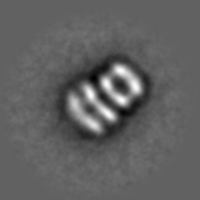

Supplement: Supplementary file 7 — Source data Fig. 2 [file 44318_2025_575_MOESM7_ESM.zip › Figure 2/2C/2d-classes_dN-ClpC-DWB-F436A+ClpP/clsum_10036.tif]

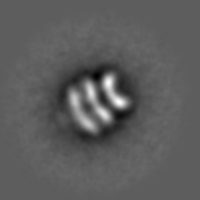

Supplement: Supplementary file 7 — Source data Fig. 2 [file 44318_2025_575_MOESM7_ESM.zip › Figure 2/2C/2d-classes_dN-ClpC-DWB-F436A+ClpP/clsum_1006.tif]

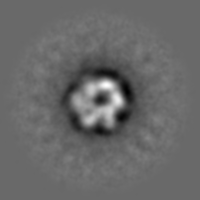

Supplement: Supplementary file 7 — Source data Fig. 2 [file 44318_2025_575_MOESM7_ESM.zip › Figure 2/2C/2d-classes_dN-ClpC-DWB-F436A+ClpP/clsum_1007.tif]

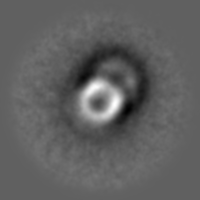

Supplement: Supplementary file 7 — Source data Fig. 2 [file 44318_2025_575_MOESM7_ESM.zip › Figure 2/2C/2d-classes_dN-ClpC-DWB-F436A+ClpP/clsum_10037.tif]

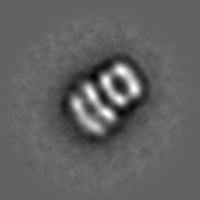

Supplement: Supplementary file 7 — Source data Fig. 2 [file 44318_2025_575_MOESM7_ESM.zip › Figure 2/2C/2d-classes_dN-ClpC-DWB-F436A+ClpP/clsum_10023.tif]

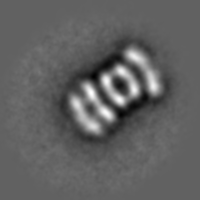

Supplement: Supplementary file 7 — Source data Fig. 2 [file 44318_2025_575_MOESM7_ESM.zip › Figure 2/2C/2d-classes_dN-ClpC-DWB-F436A+ClpP/clsum_10035.tif]

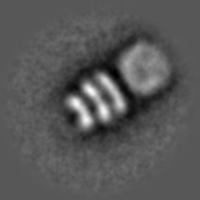

Supplement: Supplementary file 7 — Source data Fig. 2 [file 44318_2025_575_MOESM7_ESM.zip › Figure 2/2C/2d-classes_dN-ClpC-DWB-F436A+ClpP/clsum_10021.tif]

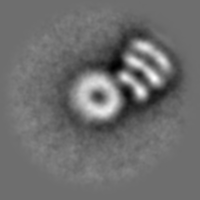

Supplement: Supplementary file 7 — Source data Fig. 2 [file 44318_2025_575_MOESM7_ESM.zip › Figure 2/2C/2d-classes_dN-ClpC-DWB-F436A+ClpP/clsum_1005.tif]

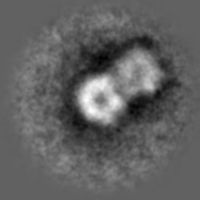

Supplement: Supplementary file 7 — Source data Fig. 2 [file 44318_2025_575_MOESM7_ESM.zip › Figure 2/2C/2d-classes_dN-ClpC-DWB-F436A+ClpP/clsum_1004.tif]

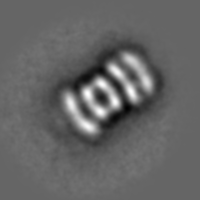

Supplement: Supplementary file 7 — Source data Fig. 2 [file 44318_2025_575_MOESM7_ESM.zip › Figure 2/2C/2d-classes_dN-ClpC-DWB-F436A+ClpP/clsum_10020.tif]

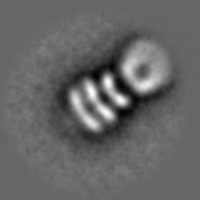

Supplement: Supplementary file 7 — Source data Fig. 2 [file 44318_2025_575_MOESM7_ESM.zip › Figure 2/2C/2d-classes_dN-ClpC-DWB-F436A+ClpP/clsum_10034.tif]

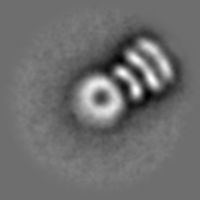

Supplement: Supplementary file 7 — Source data Fig. 2 [file 44318_2025_575_MOESM7_ESM.zip › Figure 2/2C/2d-classes_dN-ClpC-DWB-F436A+ClpP/clsum_10018.tif]

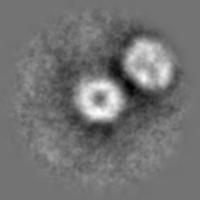

Supplement: Supplementary file 7 — Source data Fig. 2 [file 44318_2025_575_MOESM7_ESM.zip › Figure 2/2C/2d-classes_dN-ClpC-DWB-F436A+ClpP/clsum_10030.tif]

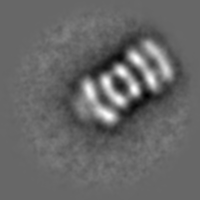

Supplement: Supplementary file 7 — Source data Fig. 2 [file 44318_2025_575_MOESM7_ESM.zip › Figure 2/2C/2d-classes_dN-ClpC-DWB-F436A+ClpP/clsum_10024.tif]

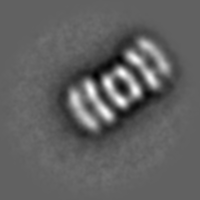

Supplement: Supplementary file 7 — Source data Fig. 2 [file 44318_2025_575_MOESM7_ESM.zip › Figure 2/2C/2d-classes_dN-ClpC-DWB-F436A+ClpP/clsum_1001.tif]

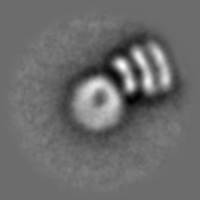

Supplement: Supplementary file 7 — Source data Fig. 2 [file 44318_2025_575_MOESM7_ESM.zip › Figure 2/2C/2d-classes_dN-ClpC-DWB-F436A+ClpP/clsum_10025.tif]

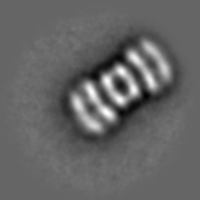

Supplement: Supplementary file 7 — Source data Fig. 2 [file 44318_2025_575_MOESM7_ESM.zip › Figure 2/2C/2d-classes_dN-ClpC-DWB-F436A+ClpP/clsum_10031.tif]

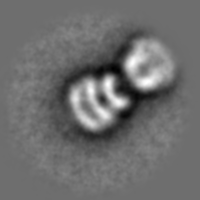

Supplement: Supplementary file 7 — Source data Fig. 2 [file 44318_2025_575_MOESM7_ESM.zip › Figure 2/2C/2d-classes_dN-ClpC-DWB-F436A+ClpP/clsum_10019.tif]

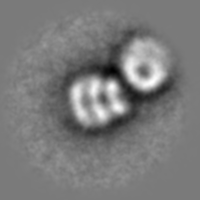

Supplement: Supplementary file 7 — Source data Fig. 2 [file 44318_2025_575_MOESM7_ESM.zip › Figure 2/2C/2d-classes_dN-ClpC-DWB-F436A+ClpP/clsum_10027.tif]

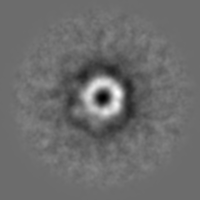

Supplement: Supplementary file 7 — Source data Fig. 2 [file 44318_2025_575_MOESM7_ESM.zip › Figure 2/2C/2d-classes_dN-ClpC-DWB-F436A+ClpP/clsum_10033.tif]

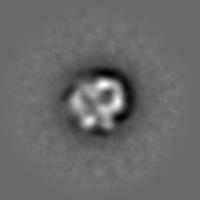

Supplement: Supplementary file 7 — Source data Fig. 2 [file 44318_2025_575_MOESM7_ESM.zip › Figure 2/2C/2d-classes_dN-ClpC-DWB-F436A+ClpP/clsum_1003.tif]

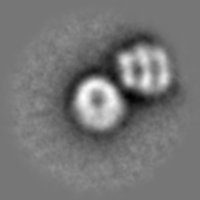

Supplement: Supplementary file 7 — Source data Fig. 2 [file 44318_2025_575_MOESM7_ESM.zip › Figure 2/2C/2d-classes_dN-ClpC-DWB-F436A+ClpP/clsum_1002.tif]

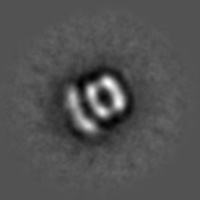

Supplement: Supplementary file 7 — Source data Fig. 2 [file 44318_2025_575_MOESM7_ESM.zip › Figure 2/2C/2d-classes_dN-ClpC-DWB-F436A+ClpP/clsum_10032.tif]

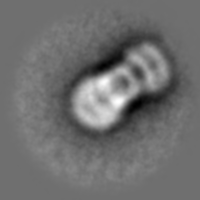

Supplement: Supplementary file 7 — Source data Fig. 2 [file 44318_2025_575_MOESM7_ESM.zip › Figure 2/2C/2d-classes_dN-ClpC-DWB-F436A+ClpP/clsum_10026.tif]

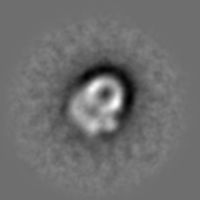

Supplement: Supplementary file 7 — Source data Fig. 2 [file 44318_2025_575_MOESM7_ESM.zip › Figure 2/2C/2d-classes_dN-ClpC-DWB-F436A+ClpP/clsum_10082.tif]

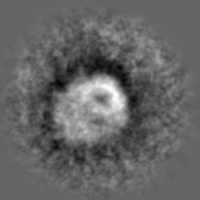

Supplement: Supplementary file 7 — Source data Fig. 2 [file 44318_2025_575_MOESM7_ESM.zip › Figure 2/2C/2d-classes_dN-ClpC-DWB-F436A+ClpP/clsum_10096.tif]

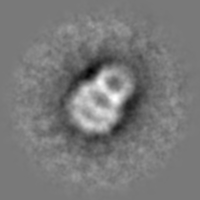

Supplement: Supplementary file 7 — Source data Fig. 2 [file 44318_2025_575_MOESM7_ESM.zip › Figure 2/2C/2d-classes_dN-ClpC-DWB-F436A+ClpP/clsum_10041.tif]

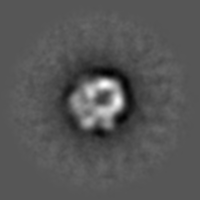

Supplement: Supplementary file 7 — Source data Fig. 2 [file 44318_2025_575_MOESM7_ESM.zip › Figure 2/2C/2d-classes_dN-ClpC-DWB-F436A+ClpP/clsum_10055.tif]

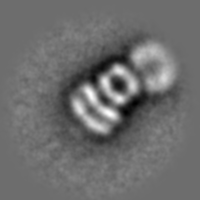

Supplement: Supplementary file 7 — Source data Fig. 2 [file 44318_2025_575_MOESM7_ESM.zip › Figure 2/2C/2d-classes_dN-ClpC-DWB-F436A+ClpP/clsum_10069.tif]

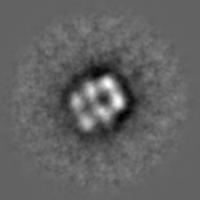

Supplement: Supplementary file 7 — Source data Fig. 2 [file 44318_2025_575_MOESM7_ESM.zip › Figure 2/2C/2d-classes_dN-ClpC-DWB-F436A+ClpP/clsum_10068.tif]

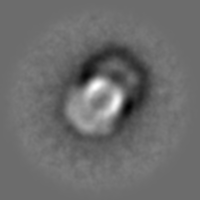

Supplement: Supplementary file 7 — Source data Fig. 2 [file 44318_2025_575_MOESM7_ESM.zip › Figure 2/2C/2d-classes_dN-ClpC-DWB-F436A+ClpP/clsum_10054.tif]

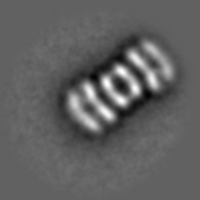

Supplement: Supplementary file 7 — Source data Fig. 2 [file 44318_2025_575_MOESM7_ESM.zip › Figure 2/2C/2d-classes_dN-ClpC-DWB-F436A+ClpP/clsum_10040.tif]

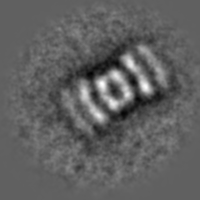

Supplement: Supplementary file 7 — Source data Fig. 2 [file 44318_2025_575_MOESM7_ESM.zip › Figure 2/2C/2d-classes_dN-ClpC-DWB-F436A+ClpP/clsum_10097.tif]

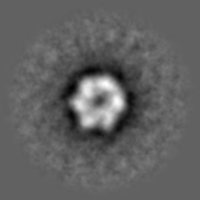

Supplement: Supplementary file 7 — Source data Fig. 2 [file 44318_2025_575_MOESM7_ESM.zip › Figure 2/2C/2d-classes_dN-ClpC-DWB-F436A+ClpP/clsum_10083.tif]

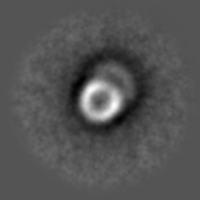

Supplement: Supplementary file 7 — Source data Fig. 2 [file 44318_2025_575_MOESM7_ESM.zip › Figure 2/2C/2d-classes_dN-ClpC-DWB-F436A+ClpP/clsum_10095.tif]

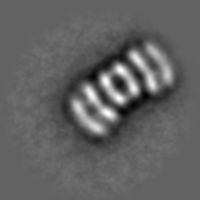

Supplement: Supplementary file 7 — Source data Fig. 2 [file 44318_2025_575_MOESM7_ESM.zip › Figure 2/2C/2d-classes_dN-ClpC-DWB-F436A+ClpP/clsum_10081.tif]

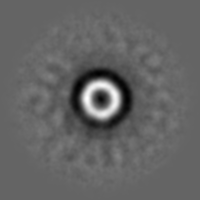

Supplement: Supplementary file 7 — Source data Fig. 2 [file 44318_2025_575_MOESM7_ESM.zip › Figure 2/2C/2d-classes_dN-ClpC-DWB-F436A+ClpP/clsum_10056.tif]

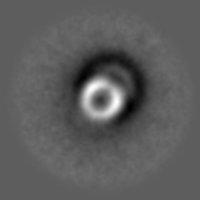

Supplement: Supplementary file 7 — Source data Fig. 2 [file 44318_2025_575_MOESM7_ESM.zip › Figure 2/2C/2d-classes_dN-ClpC-DWB-F436A+ClpP/clsum_10042.tif]

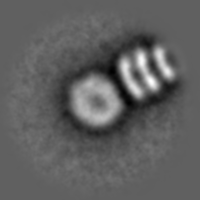

Supplement: Supplementary file 7 — Source data Fig. 2 [file 44318_2025_575_MOESM7_ESM.zip › Figure 2/2C/2d-classes_dN-ClpC-DWB-F436A+ClpP/clsum_10043.tif]

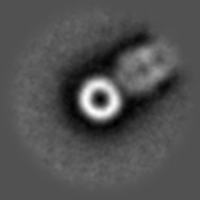

Supplement: Supplementary file 7 — Source data Fig. 2 [file 44318_2025_575_MOESM7_ESM.zip › Figure 2/2C/2d-classes_dN-ClpC-DWB-F436A+ClpP/clsum_10057.tif]

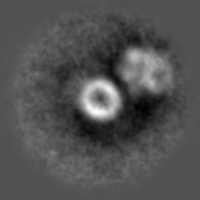

Supplement: Supplementary file 7 — Source data Fig. 2 [file 44318_2025_575_MOESM7_ESM.zip › Figure 2/2C/2d-classes_dN-ClpC-DWB-F436A+ClpP/clsum_10080.tif]

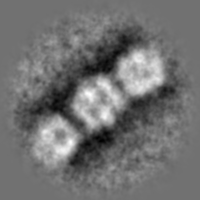

Supplement: Supplementary file 7 — Source data Fig. 2 [file 44318_2025_575_MOESM7_ESM.zip › Figure 2/2C/2d-classes_dN-ClpC-DWB-F436A+ClpP/clsum_10094.tif]

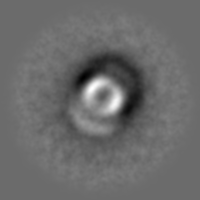

Supplement: Supplementary file 7 — Source data Fig. 2 [file 44318_2025_575_MOESM7_ESM.zip › Figure 2/2C/2d-classes_dN-ClpC-DWB-F436A+ClpP/clsum_10090.tif]

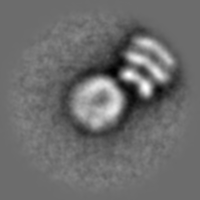

Supplement: Supplementary file 7 — Source data Fig. 2 [file 44318_2025_575_MOESM7_ESM.zip › Figure 2/2C/2d-classes_dN-ClpC-DWB-F436A+ClpP/clsum_10084.tif]

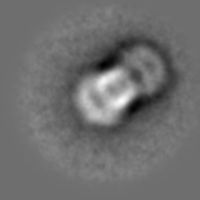

Supplement: Supplementary file 7 — Source data Fig. 2 [file 44318_2025_575_MOESM7_ESM.zip › Figure 2/2C/2d-classes_dN-ClpC-DWB-F436A+ClpP/clsum_10053.tif]

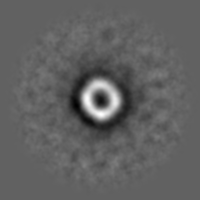

Supplement: Supplementary file 7 — Source data Fig. 2 [file 44318_2025_575_MOESM7_ESM.zip › Figure 2/2C/2d-classes_dN-ClpC-DWB-F436A+ClpP/clsum_10047.tif]

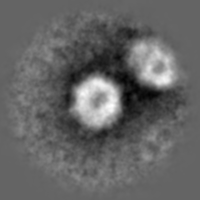

Supplement: Supplementary file 7 — Source data Fig. 2 [file 44318_2025_575_MOESM7_ESM.zip › Figure 2/2C/2d-classes_dN-ClpC-DWB-F436A+ClpP/clsum_10046.tif]

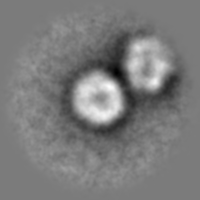

Supplement: Supplementary file 7 — Source data Fig. 2 [file 44318_2025_575_MOESM7_ESM.zip › Figure 2/2C/2d-classes_dN-ClpC-DWB-F436A+ClpP/clsum_10052.tif]

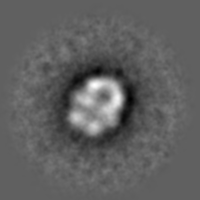

Supplement: Supplementary file 7 — Source data Fig. 2 [file 44318_2025_575_MOESM7_ESM.zip › Figure 2/2C/2d-classes_dN-ClpC-DWB-F436A+ClpP/clsum_10085.tif]

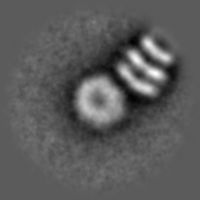

Supplement: Supplementary file 7 — Source data Fig. 2 [file 44318_2025_575_MOESM7_ESM.zip › Figure 2/2C/2d-classes_dN-ClpC-DWB-F436A+ClpP/clsum_10091.tif]

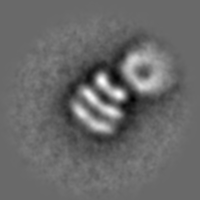

Supplement: Supplementary file 7 — Source data Fig. 2 [file 44318_2025_575_MOESM7_ESM.zip › Figure 2/2C/2d-classes_dN-ClpC-DWB-F436A+ClpP/clsum_10087.tif]

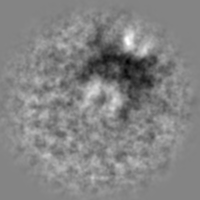

Supplement: Supplementary file 7 — Source data Fig. 2 [file 44318_2025_575_MOESM7_ESM.zip › Figure 2/2C/2d-classes_dN-ClpC-DWB-F436A+ClpP/clsum_10093.tif]

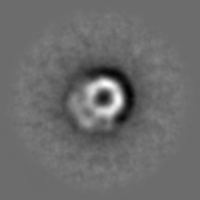

Supplement: Supplementary file 7 — Source data Fig. 2 [file 44318_2025_575_MOESM7_ESM.zip › Figure 2/2C/2d-classes_dN-ClpC-DWB-F436A+ClpP/clsum_10078.tif]

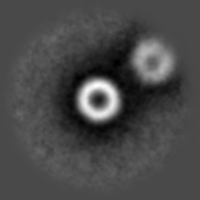

Supplement: Supplementary file 7 — Source data Fig. 2 [file 44318_2025_575_MOESM7_ESM.zip › Figure 2/2C/2d-classes_dN-ClpC-DWB-F436A+ClpP/clsum_10044.tif]

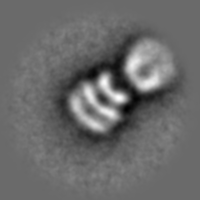

Supplement: Supplementary file 7 — Source data Fig. 2 [file 44318_2025_575_MOESM7_ESM.zip › Figure 2/2C/2d-classes_dN-ClpC-DWB-F436A+ClpP/clsum_10050.tif]
