# Supplementary figures and images for: Allosteric control of the bacterial ClpC/ClpP protease and its hijacking by antibacterial peptides (part 5 of 5)
Source: EMBO J. 2025 Sep 29;44(21):6273–96. doi: 10.1038/s44318-025-00575-1 (PMC12583610; doi:10.1038/s44318-025-00575-1)

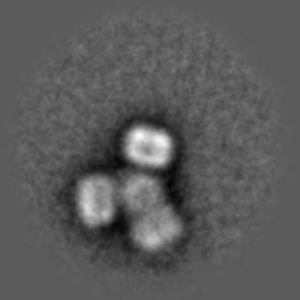

Supplement: Supplementary file 10 — Source data Fig. 6 [file 44318_2025_575_MOESM10_ESM.zip › Figure 6/6E/2d-classes_ClpC-D356A-DWB+FITC-cas/clsum_50-015.tif]

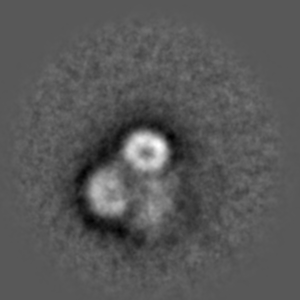

Supplement: Supplementary file 10 — Source data Fig. 6 [file 44318_2025_575_MOESM10_ESM.zip › Figure 6/6E/2d-classes_ClpC-D356A-DWB+FITC-cas/clsum_50-001.tif]

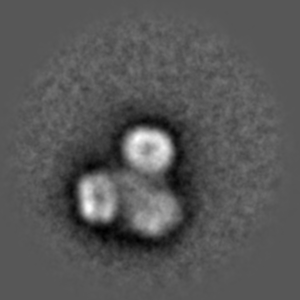

Supplement: Supplementary file 10 — Source data Fig. 6 [file 44318_2025_575_MOESM10_ESM.zip › Figure 6/6E/2d-classes_ClpC-D356A-DWB+FITC-cas/clsum_50-029.tif]

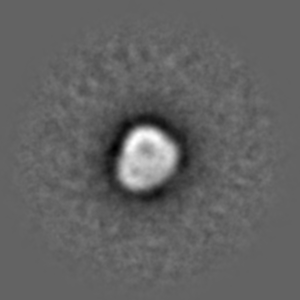

Supplement: Supplementary file 10 — Source data Fig. 6 [file 44318_2025_575_MOESM10_ESM.zip › Figure 6/6E/2d-classes_ClpC-D356A-DWB+FITC-cas/clsum_50-028.tif]

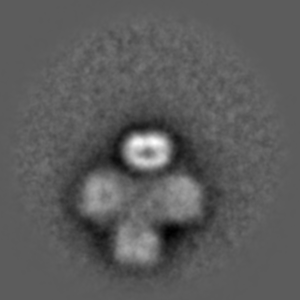

Supplement: Supplementary file 10 — Source data Fig. 6 [file 44318_2025_575_MOESM10_ESM.zip › Figure 6/6E/2d-classes_ClpC-D356A-DWB+FITC-cas/clsum_50-014.tif]

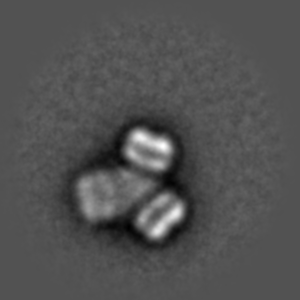

Supplement: Supplementary file 10 — Source data Fig. 6 [file 44318_2025_575_MOESM10_ESM.zip › Figure 6/6E/2d-classes_ClpC-D356A-DWB+FITC-cas/clsum_50-038.tif]

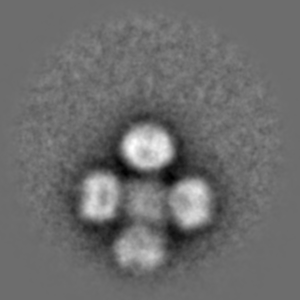

Supplement: Supplementary file 10 — Source data Fig. 6 [file 44318_2025_575_MOESM10_ESM.zip › Figure 6/6E/2d-classes_ClpC-D356A-DWB+FITC-cas/clsum_50-010.tif]

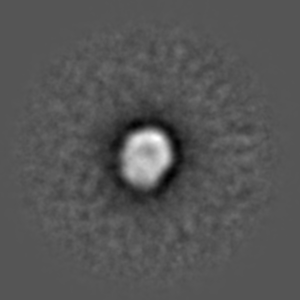

Supplement: Supplementary file 10 — Source data Fig. 6 [file 44318_2025_575_MOESM10_ESM.zip › Figure 6/6E/2d-classes_ClpC-D356A-DWB+FITC-cas/clsum_50-004.tif]

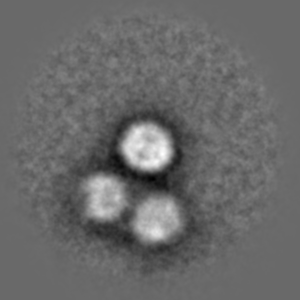

Supplement: Supplementary file 10 — Source data Fig. 6 [file 44318_2025_575_MOESM10_ESM.zip › Figure 6/6E/2d-classes_ClpC-D356A-DWB+FITC-cas/clsum_50-005.tif]

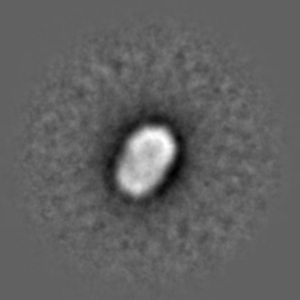

Supplement: Supplementary file 10 — Source data Fig. 6 [file 44318_2025_575_MOESM10_ESM.zip › Figure 6/6E/2d-classes_ClpC-D356A-DWB+FITC-cas/clsum_50-011.tif]

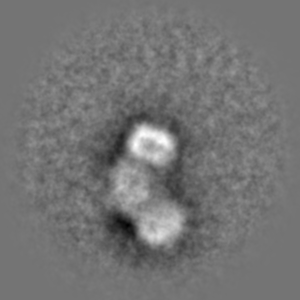

Supplement: Supplementary file 10 — Source data Fig. 6 [file 44318_2025_575_MOESM10_ESM.zip › Figure 6/6E/2d-classes_ClpC-D356A-DWB+FITC-cas/clsum_50-039.tif]

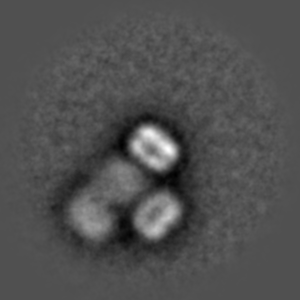

Supplement: Supplementary file 10 — Source data Fig. 6 [file 44318_2025_575_MOESM10_ESM.zip › Figure 6/6E/2d-classes_ClpC-D356A-DWB+FITC-cas/clsum_50-007.tif]

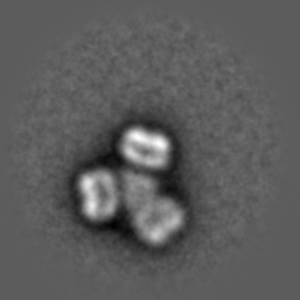

Supplement: Supplementary file 10 — Source data Fig. 6 [file 44318_2025_575_MOESM10_ESM.zip › Figure 6/6E/2d-classes_ClpC-D356A-DWB+FITC-cas/clsum_50-013.tif]

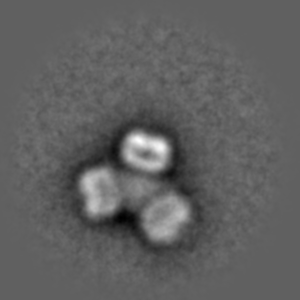

Supplement: Supplementary file 10 — Source data Fig. 6 [file 44318_2025_575_MOESM10_ESM.zip › Figure 6/6E/2d-classes_ClpC-D356A-DWB+FITC-cas/clsum_50-012.tif]

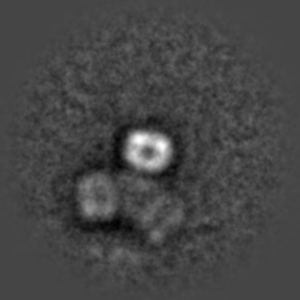

Supplement: Supplementary file 10 — Source data Fig. 6 [file 44318_2025_575_MOESM10_ESM.zip › Figure 6/6E/2d-classes_ClpC-D356A-DWB+FITC-cas/clsum_50-006.tif]

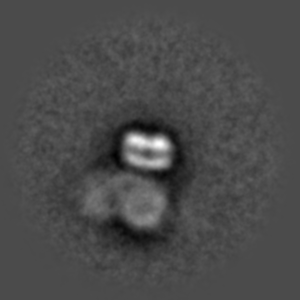

Supplement: Supplementary file 10 — Source data Fig. 6 [file 44318_2025_575_MOESM10_ESM.zip › Figure 6/6E/2d-classes_ClpC-D356A-DWB+FITC-cas/clsum_50-023.tif]

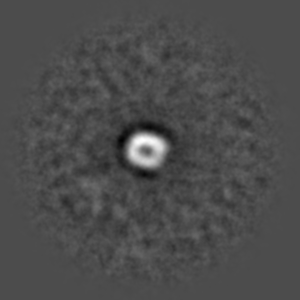

Supplement: Supplementary file 10 — Source data Fig. 6 [file 44318_2025_575_MOESM10_ESM.zip › Figure 6/6E/2d-classes_ClpC-D356A-DWB+FITC-cas/clsum_50-037.tif]

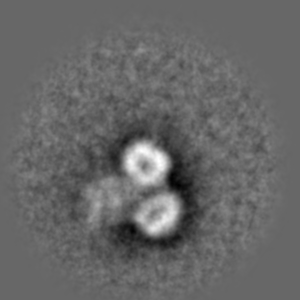

Supplement: Supplementary file 10 — Source data Fig. 6 [file 44318_2025_575_MOESM10_ESM.zip › Figure 6/6E/2d-classes_ClpC-D356A-DWB+FITC-cas/clsum_50-036.tif]

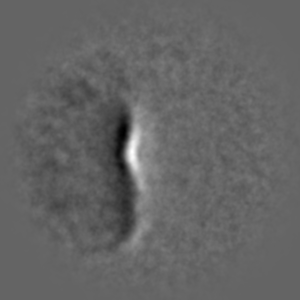

Supplement: Supplementary file 10 — Source data Fig. 6 [file 44318_2025_575_MOESM10_ESM.zip › Figure 6/6E/2d-classes_ClpC-D356A-DWB+FITC-cas/clsum_50-022.tif]

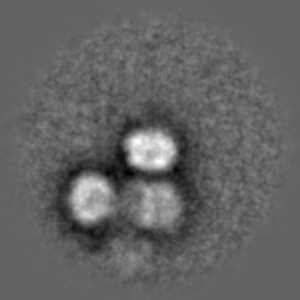

Supplement: Supplementary file 10 — Source data Fig. 6 [file 44318_2025_575_MOESM10_ESM.zip › Figure 6/6E/2d-classes_ClpC-D356A-DWB+FITC-cas/clsum_50-034.tif]

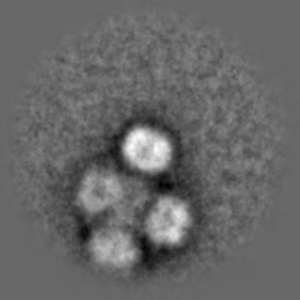

Supplement: Supplementary file 10 — Source data Fig. 6 [file 44318_2025_575_MOESM10_ESM.zip › Figure 6/6E/2d-classes_ClpC-D356A-DWB+FITC-cas/clsum_50-020.tif]

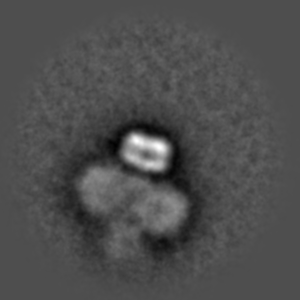

Supplement: Supplementary file 10 — Source data Fig. 6 [file 44318_2025_575_MOESM10_ESM.zip › Figure 6/6E/2d-classes_ClpC-D356A-DWB+FITC-cas/clsum_50-008.tif]

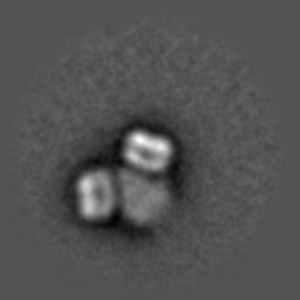

Supplement: Supplementary file 10 — Source data Fig. 6 [file 44318_2025_575_MOESM10_ESM.zip › Figure 6/6E/2d-classes_ClpC-D356A-DWB+FITC-cas/clsum_50-009.tif]

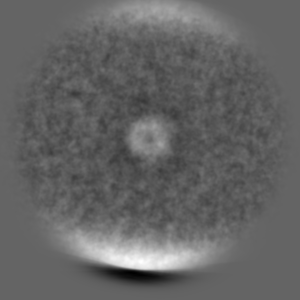

Supplement: Supplementary file 10 — Source data Fig. 6 [file 44318_2025_575_MOESM10_ESM.zip › Figure 6/6E/2d-classes_ClpC-D356A-DWB+FITC-cas/clsum_50-021.tif]

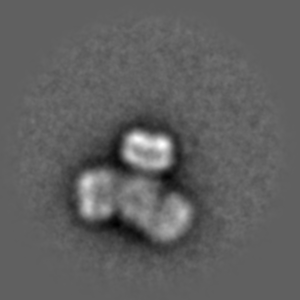

Supplement: Supplementary file 10 — Source data Fig. 6 [file 44318_2025_575_MOESM10_ESM.zip › Figure 6/6E/2d-classes_ClpC-D356A-DWB+FITC-cas/clsum_50-035.tif]

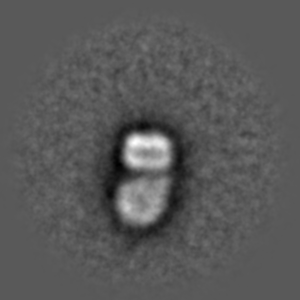

Supplement: Supplementary file 10 — Source data Fig. 6 [file 44318_2025_575_MOESM10_ESM.zip › Figure 6/6E/2d-classes_ClpC-D356A-DWB+FITC-cas/clsum_50-019.tif]

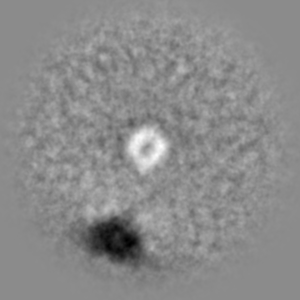

Supplement: Supplementary file 10 — Source data Fig. 6 [file 44318_2025_575_MOESM10_ESM.zip › Figure 6/6E/2d-classes_ClpC-D356A-DWB+FITC-cas/clsum_50-031.tif]

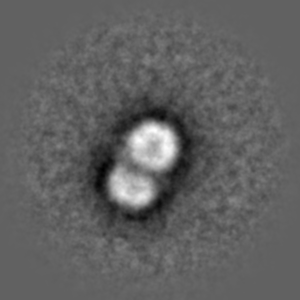

Supplement: Supplementary file 10 — Source data Fig. 6 [file 44318_2025_575_MOESM10_ESM.zip › Figure 6/6E/2d-classes_ClpC-D356A-DWB+FITC-cas/clsum_50-025.tif]

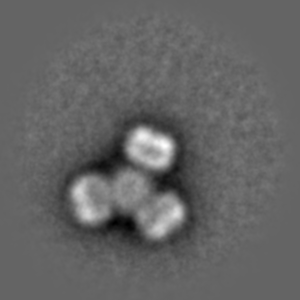

Supplement: Supplementary file 10 — Source data Fig. 6 [file 44318_2025_575_MOESM10_ESM.zip › Figure 6/6E/2d-classes_ClpC-D356A-DWB+FITC-cas/clsum_50-024.tif]

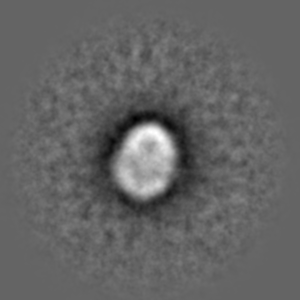

Supplement: Supplementary file 10 — Source data Fig. 6 [file 44318_2025_575_MOESM10_ESM.zip › Figure 6/6E/2d-classes_ClpC-D356A-DWB+FITC-cas/clsum_50-030.tif]

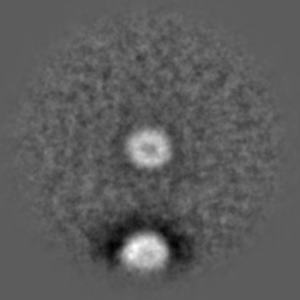

Supplement: Supplementary file 10 — Source data Fig. 6 [file 44318_2025_575_MOESM10_ESM.zip › Figure 6/6E/2d-classes_ClpC-D356A-DWB+FITC-cas/clsum_50-018.tif]

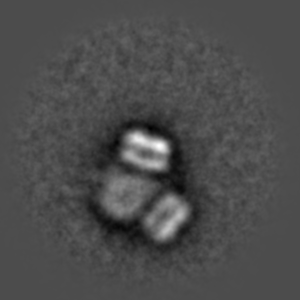

Supplement: Supplementary file 10 — Source data Fig. 6 [file 44318_2025_575_MOESM10_ESM.zip › Figure 6/6E/2d-classes_ClpC-D356A-DWB+FITC-cas/clsum_50-026.tif]

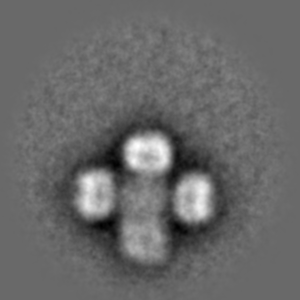

Supplement: Supplementary file 10 — Source data Fig. 6 [file 44318_2025_575_MOESM10_ESM.zip › Figure 6/6E/2d-classes_ClpC-D356A-DWB+FITC-cas/clsum_50-032.tif]

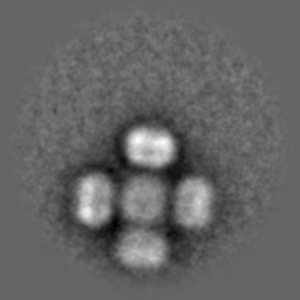

Supplement: Supplementary file 10 — Source data Fig. 6 [file 44318_2025_575_MOESM10_ESM.zip › Figure 6/6E/2d-classes_ClpC-D356A-DWB+FITC-cas/clsum_50-033.tif]

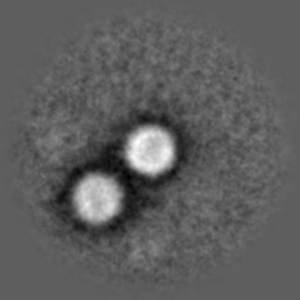

Supplement: Supplementary file 10 — Source data Fig. 6 [file 44318_2025_575_MOESM10_ESM.zip › Figure 6/6E/2d-classes_ClpC-D356A-DWB+FITC-cas/clsum_50-027.tif]

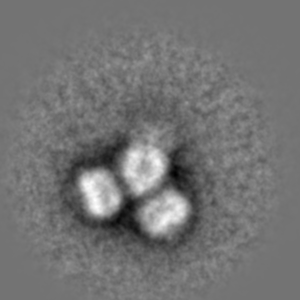

Supplement: Supplementary file 10 — Source data Fig. 6 [file 44318_2025_575_MOESM10_ESM.zip › Figure 6/6E/2d-classes_ClpC-D356A-DWB+FITC-cas/clsum_50-040.tif]

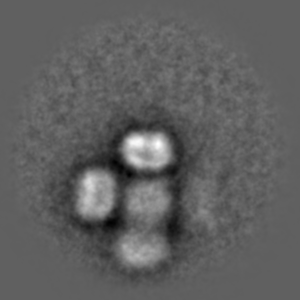

Supplement: Supplementary file 10 — Source data Fig. 6 [file 44318_2025_575_MOESM10_ESM.zip › Figure 6/6E/2d-classes_ClpC-D356A-DWB+FITC-cas/clsum_50-041.tif]

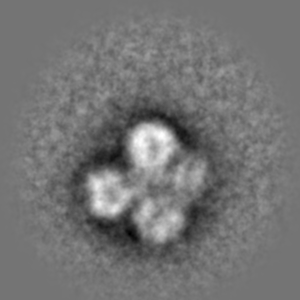

Supplement: Supplementary file 10 — Source data Fig. 6 [file 44318_2025_575_MOESM10_ESM.zip › Figure 6/6E/2d-classes_ClpC-D356A-DWB+FITC-cas/clsum_50-043.tif]

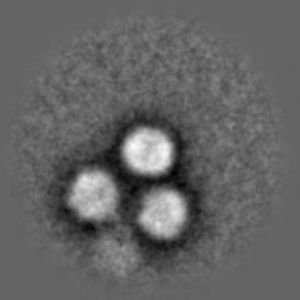

Supplement: Supplementary file 10 — Source data Fig. 6 [file 44318_2025_575_MOESM10_ESM.zip › Figure 6/6E/2d-classes_ClpC-D356A-DWB+FITC-cas/clsum_50-042.tif]

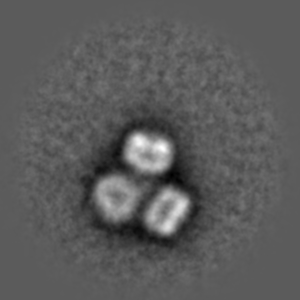

Supplement: Supplementary file 10 — Source data Fig. 6 [file 44318_2025_575_MOESM10_ESM.zip › Figure 6/6E/2d-classes_ClpC-D356A-DWB+FITC-cas/clsum_50-046.tif]

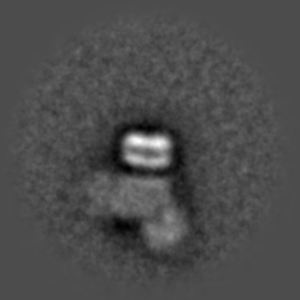

Supplement: Supplementary file 10 — Source data Fig. 6 [file 44318_2025_575_MOESM10_ESM.zip › Figure 6/6E/2d-classes_ClpC-D356A-DWB+FITC-cas/clsum_50-047.tif]

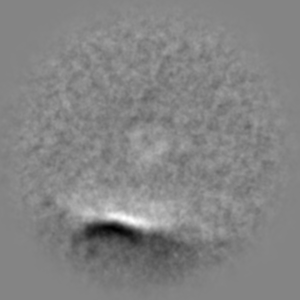

Supplement: Supplementary file 10 — Source data Fig. 6 [file 44318_2025_575_MOESM10_ESM.zip › Figure 6/6E/2d-classes_ClpC-D356A-DWB+FITC-cas/clsum_50-045.tif]

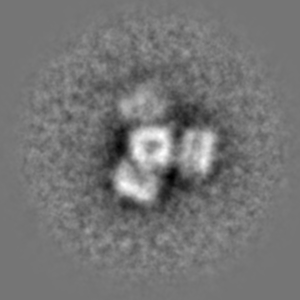

Supplement: Supplementary file 10 — Source data Fig. 6 [file 44318_2025_575_MOESM10_ESM.zip › Figure 6/6E/2d-classes_ClpC-D356A-DWB+FITC-cas/clsum_50-050.tif]

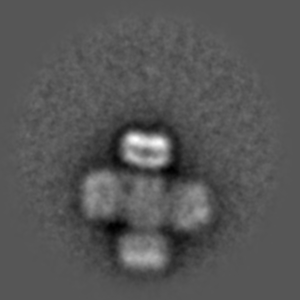

Supplement: Supplementary file 10 — Source data Fig. 6 [file 44318_2025_575_MOESM10_ESM.zip › Figure 6/6E/2d-classes_ClpC-D356A-DWB+FITC-cas/clsum_50-044.tif]

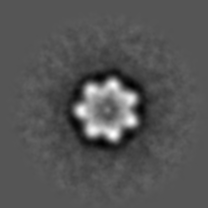

Supplement: Supplementary file 10 — Source data Fig. 6 [file 44318_2025_575_MOESM10_ESM.zip › Figure 6/6E/2d-classes_ClpC-D356A/clsum_50-049.tif]

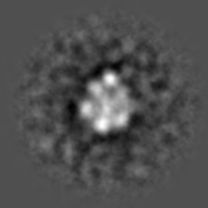

Supplement: Supplementary file 10 — Source data Fig. 6 [file 44318_2025_575_MOESM10_ESM.zip › Figure 6/6E/2d-classes_ClpC-D356A/clsum_50-048.tif]

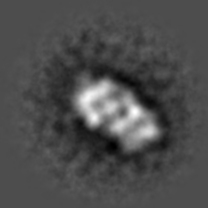

Supplement: Supplementary file 10 — Source data Fig. 6 [file 44318_2025_575_MOESM10_ESM.zip › Figure 6/6E/2d-classes_ClpC-D356A/clsum_50-002.tif]

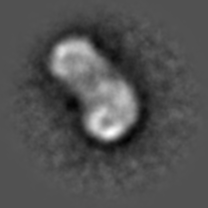

Supplement: Supplementary file 10 — Source data Fig. 6 [file 44318_2025_575_MOESM10_ESM.zip › Figure 6/6E/2d-classes_ClpC-D356A/clsum_50-016.tif]

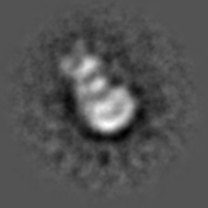

Supplement: Supplementary file 10 — Source data Fig. 6 [file 44318_2025_575_MOESM10_ESM.zip › Figure 6/6E/2d-classes_ClpC-D356A/clsum_50-017.tif]

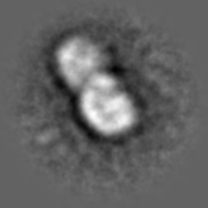

Supplement: Supplementary file 10 — Source data Fig. 6 [file 44318_2025_575_MOESM10_ESM.zip › Figure 6/6E/2d-classes_ClpC-D356A/clsum_50-003.tif]

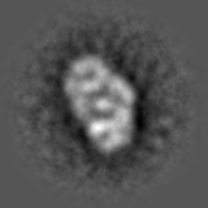

Supplement: Supplementary file 10 — Source data Fig. 6 [file 44318_2025_575_MOESM10_ESM.zip › Figure 6/6E/2d-classes_ClpC-D356A/clsum_50-015.tif]

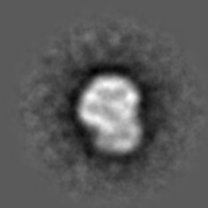

Supplement: Supplementary file 10 — Source data Fig. 6 [file 44318_2025_575_MOESM10_ESM.zip › Figure 6/6E/2d-classes_ClpC-D356A/clsum_50-001.tif]

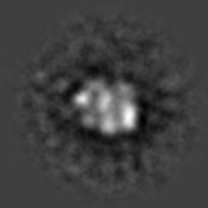

Supplement: Supplementary file 10 — Source data Fig. 6 [file 44318_2025_575_MOESM10_ESM.zip › Figure 6/6E/2d-classes_ClpC-D356A/clsum_50-029.tif]

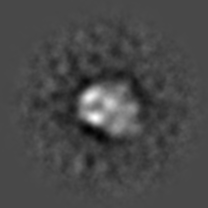

Supplement: Supplementary file 10 — Source data Fig. 6 [file 44318_2025_575_MOESM10_ESM.zip › Figure 6/6E/2d-classes_ClpC-D356A/clsum_50-028.tif]

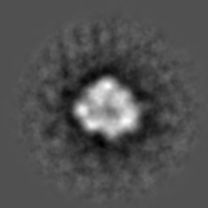

Supplement: Supplementary file 10 — Source data Fig. 6 [file 44318_2025_575_MOESM10_ESM.zip › Figure 6/6E/2d-classes_ClpC-D356A/clsum_50-014.tif]

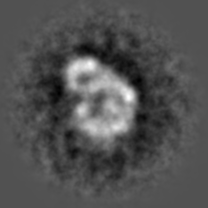

Supplement: Supplementary file 10 — Source data Fig. 6 [file 44318_2025_575_MOESM10_ESM.zip › Figure 6/6E/2d-classes_ClpC-D356A/clsum_50-038.tif]

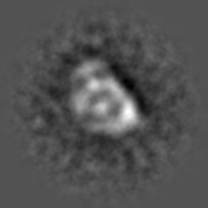

Supplement: Supplementary file 10 — Source data Fig. 6 [file 44318_2025_575_MOESM10_ESM.zip › Figure 6/6E/2d-classes_ClpC-D356A/clsum_50-010.tif]

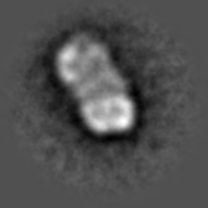

Supplement: Supplementary file 10 — Source data Fig. 6 [file 44318_2025_575_MOESM10_ESM.zip › Figure 6/6E/2d-classes_ClpC-D356A/clsum_50-004.tif]

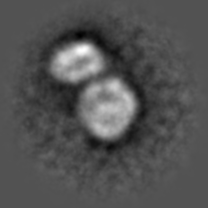

Supplement: Supplementary file 10 — Source data Fig. 6 [file 44318_2025_575_MOESM10_ESM.zip › Figure 6/6E/2d-classes_ClpC-D356A/clsum_50-005.tif]

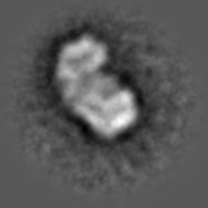

Supplement: Supplementary file 10 — Source data Fig. 6 [file 44318_2025_575_MOESM10_ESM.zip › Figure 6/6E/2d-classes_ClpC-D356A/clsum_50-011.tif]

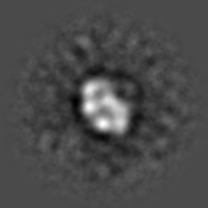

Supplement: Supplementary file 10 — Source data Fig. 6 [file 44318_2025_575_MOESM10_ESM.zip › Figure 6/6E/2d-classes_ClpC-D356A/clsum_50-039.tif]

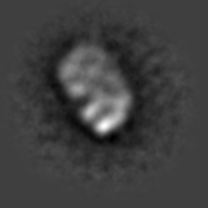

Supplement: Supplementary file 10 — Source data Fig. 6 [file 44318_2025_575_MOESM10_ESM.zip › Figure 6/6E/2d-classes_ClpC-D356A/clsum_50-007.tif]

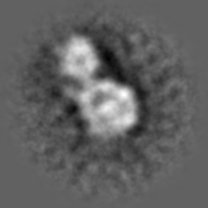

Supplement: Supplementary file 10 — Source data Fig. 6 [file 44318_2025_575_MOESM10_ESM.zip › Figure 6/6E/2d-classes_ClpC-D356A/clsum_50-013.tif]

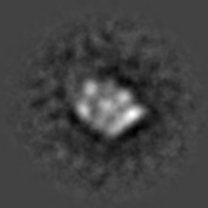

Supplement: Supplementary file 10 — Source data Fig. 6 [file 44318_2025_575_MOESM10_ESM.zip › Figure 6/6E/2d-classes_ClpC-D356A/clsum_50-012.tif]

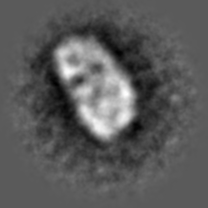

Supplement: Supplementary file 10 — Source data Fig. 6 [file 44318_2025_575_MOESM10_ESM.zip › Figure 6/6E/2d-classes_ClpC-D356A/clsum_50-006.tif]

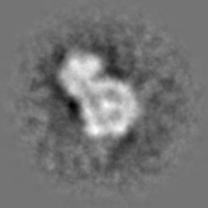

Supplement: Supplementary file 10 — Source data Fig. 6 [file 44318_2025_575_MOESM10_ESM.zip › Figure 6/6E/2d-classes_ClpC-D356A/clsum_50-023.tif]

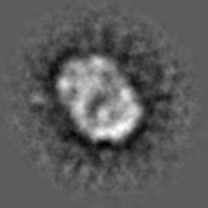

Supplement: Supplementary file 10 — Source data Fig. 6 [file 44318_2025_575_MOESM10_ESM.zip › Figure 6/6E/2d-classes_ClpC-D356A/clsum_50-037.tif]

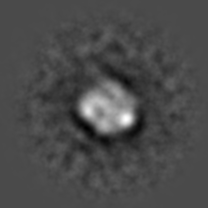

Supplement: Supplementary file 10 — Source data Fig. 6 [file 44318_2025_575_MOESM10_ESM.zip › Figure 6/6E/2d-classes_ClpC-D356A/clsum_50-036.tif]

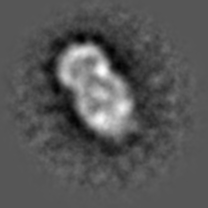

Supplement: Supplementary file 10 — Source data Fig. 6 [file 44318_2025_575_MOESM10_ESM.zip › Figure 6/6E/2d-classes_ClpC-D356A/clsum_50-022.tif]

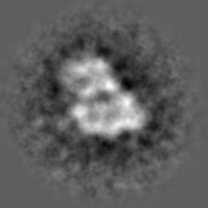

Supplement: Supplementary file 10 — Source data Fig. 6 [file 44318_2025_575_MOESM10_ESM.zip › Figure 6/6E/2d-classes_ClpC-D356A/clsum_50-034.tif]

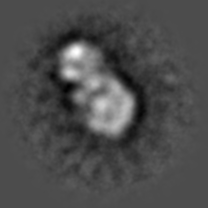

Supplement: Supplementary file 10 — Source data Fig. 6 [file 44318_2025_575_MOESM10_ESM.zip › Figure 6/6E/2d-classes_ClpC-D356A/clsum_50-020.tif]

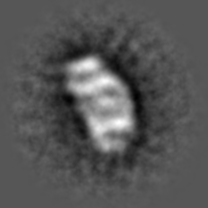

Supplement: Supplementary file 10 — Source data Fig. 6 [file 44318_2025_575_MOESM10_ESM.zip › Figure 6/6E/2d-classes_ClpC-D356A/clsum_50-008.tif]

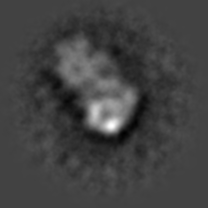

Supplement: Supplementary file 10 — Source data Fig. 6 [file 44318_2025_575_MOESM10_ESM.zip › Figure 6/6E/2d-classes_ClpC-D356A/clsum_50-009.tif]

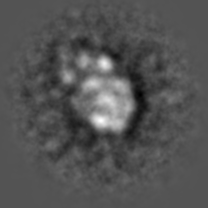

Supplement: Supplementary file 10 — Source data Fig. 6 [file 44318_2025_575_MOESM10_ESM.zip › Figure 6/6E/2d-classes_ClpC-D356A/clsum_50-021.tif]

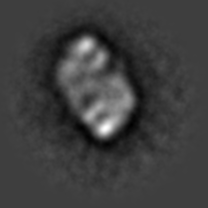

Supplement: Supplementary file 10 — Source data Fig. 6 [file 44318_2025_575_MOESM10_ESM.zip › Figure 6/6E/2d-classes_ClpC-D356A/clsum_50-035.tif]

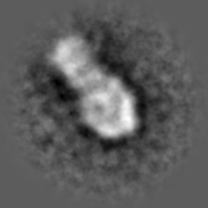

Supplement: Supplementary file 10 — Source data Fig. 6 [file 44318_2025_575_MOESM10_ESM.zip › Figure 6/6E/2d-classes_ClpC-D356A/clsum_50-019.tif]

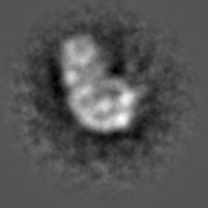

Supplement: Supplementary file 10 — Source data Fig. 6 [file 44318_2025_575_MOESM10_ESM.zip › Figure 6/6E/2d-classes_ClpC-D356A/clsum_50-031.tif]

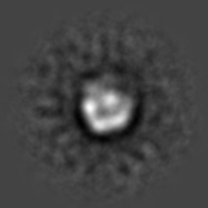

Supplement: Supplementary file 10 — Source data Fig. 6 [file 44318_2025_575_MOESM10_ESM.zip › Figure 6/6E/2d-classes_ClpC-D356A/clsum_50-025.tif]

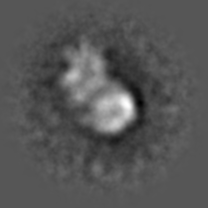

Supplement: Supplementary file 10 — Source data Fig. 6 [file 44318_2025_575_MOESM10_ESM.zip › Figure 6/6E/2d-classes_ClpC-D356A/clsum_50-024.tif]

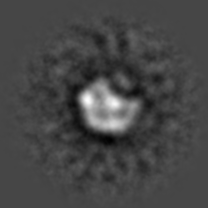

Supplement: Supplementary file 10 — Source data Fig. 6 [file 44318_2025_575_MOESM10_ESM.zip › Figure 6/6E/2d-classes_ClpC-D356A/clsum_50-030.tif]

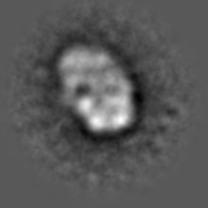

Supplement: Supplementary file 10 — Source data Fig. 6 [file 44318_2025_575_MOESM10_ESM.zip › Figure 6/6E/2d-classes_ClpC-D356A/clsum_50-018.tif]

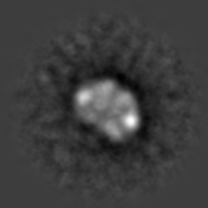

Supplement: Supplementary file 10 — Source data Fig. 6 [file 44318_2025_575_MOESM10_ESM.zip › Figure 6/6E/2d-classes_ClpC-D356A/clsum_50-026.tif]

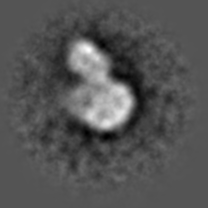

Supplement: Supplementary file 10 — Source data Fig. 6 [file 44318_2025_575_MOESM10_ESM.zip › Figure 6/6E/2d-classes_ClpC-D356A/clsum_50-032.tif]

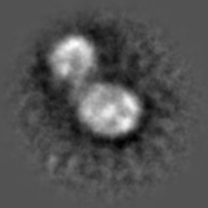

Supplement: Supplementary file 10 — Source data Fig. 6 [file 44318_2025_575_MOESM10_ESM.zip › Figure 6/6E/2d-classes_ClpC-D356A/clsum_50-033.tif]

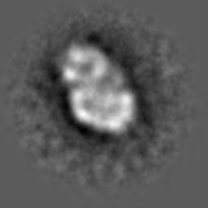

Supplement: Supplementary file 10 — Source data Fig. 6 [file 44318_2025_575_MOESM10_ESM.zip › Figure 6/6E/2d-classes_ClpC-D356A/clsum_50-027.tif]

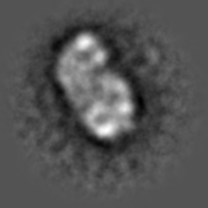

Supplement: Supplementary file 10 — Source data Fig. 6 [file 44318_2025_575_MOESM10_ESM.zip › Figure 6/6E/2d-classes_ClpC-D356A/clsum_50-040.tif]

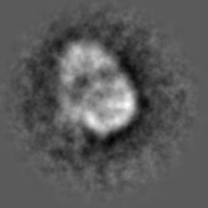

Supplement: Supplementary file 10 — Source data Fig. 6 [file 44318_2025_575_MOESM10_ESM.zip › Figure 6/6E/2d-classes_ClpC-D356A/clsum_50-041.tif]

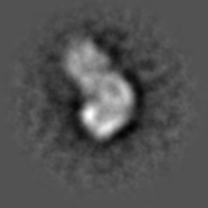

Supplement: Supplementary file 10 — Source data Fig. 6 [file 44318_2025_575_MOESM10_ESM.zip › Figure 6/6E/2d-classes_ClpC-D356A/clsum_50-043.tif]

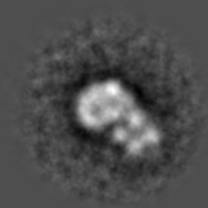

Supplement: Supplementary file 10 — Source data Fig. 6 [file 44318_2025_575_MOESM10_ESM.zip › Figure 6/6E/2d-classes_ClpC-D356A/clsum_50-042.tif]

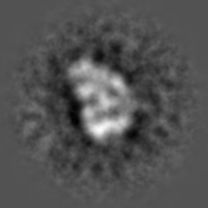

Supplement: Supplementary file 10 — Source data Fig. 6 [file 44318_2025_575_MOESM10_ESM.zip › Figure 6/6E/2d-classes_ClpC-D356A/clsum_50-046.tif]

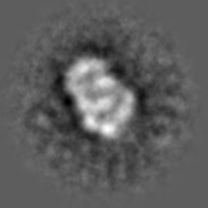

Supplement: Supplementary file 10 — Source data Fig. 6 [file 44318_2025_575_MOESM10_ESM.zip › Figure 6/6E/2d-classes_ClpC-D356A/clsum_50-047.tif]

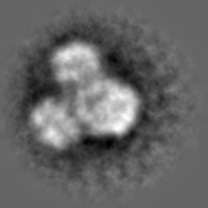

Supplement: Supplementary file 10 — Source data Fig. 6 [file 44318_2025_575_MOESM10_ESM.zip › Figure 6/6E/2d-classes_ClpC-D356A/clsum_50-045.tif]

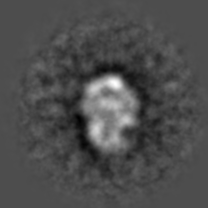

Supplement: Supplementary file 10 — Source data Fig. 6 [file 44318_2025_575_MOESM10_ESM.zip › Figure 6/6E/2d-classes_ClpC-D356A/clsum_50-050.tif]

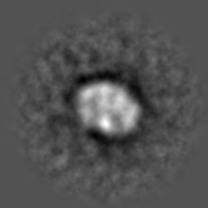

Supplement: Supplementary file 10 — Source data Fig. 6 [file 44318_2025_575_MOESM10_ESM.zip › Figure 6/6E/2d-classes_ClpC-D356A/clsum_50-044.tif]

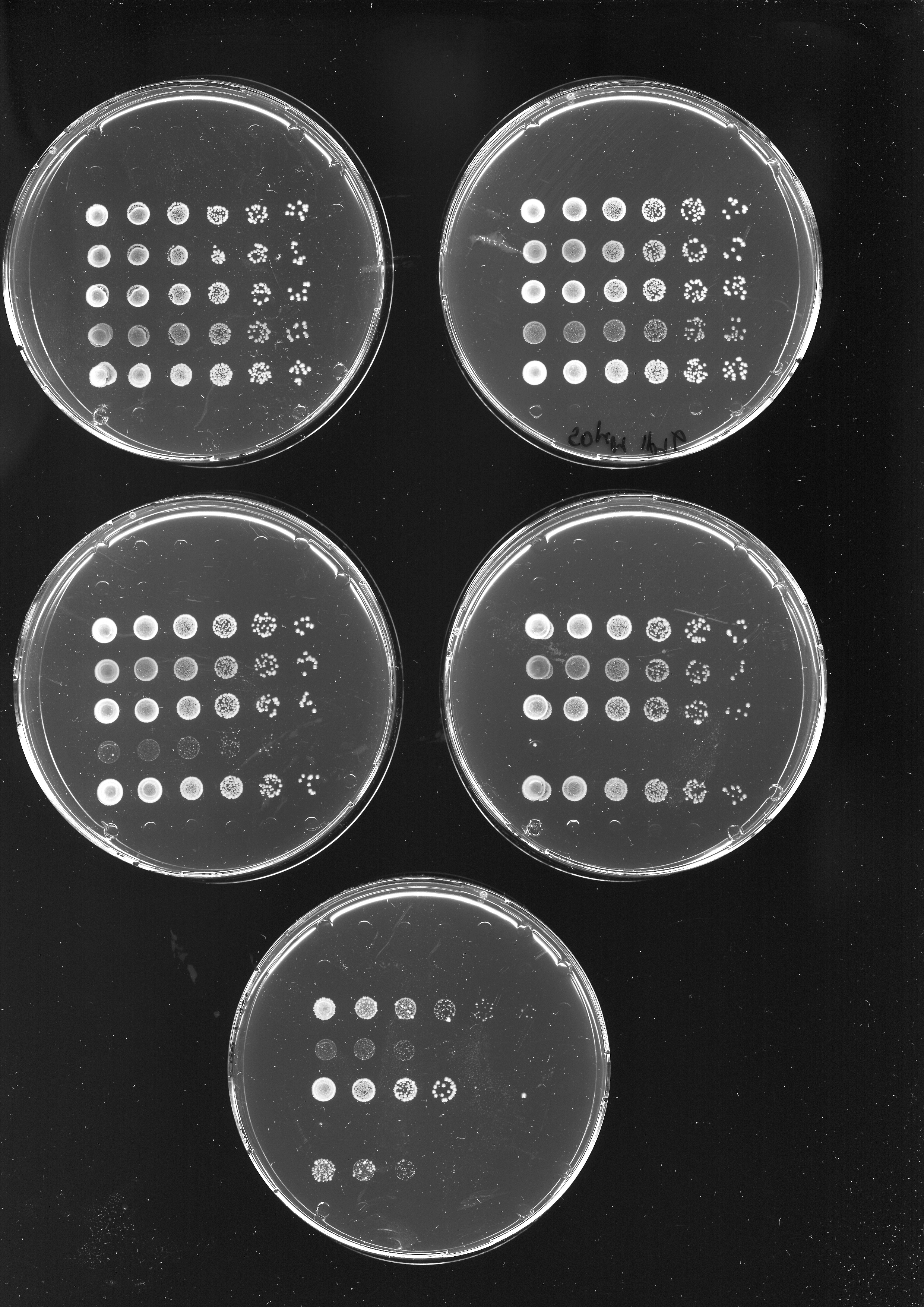

Supplement: Supplementary file 12 — Source data Fig. 8 [file 44318_2025_575_MOESM12_ESM.zip › Figure 8/exp1-30-0725.tif]

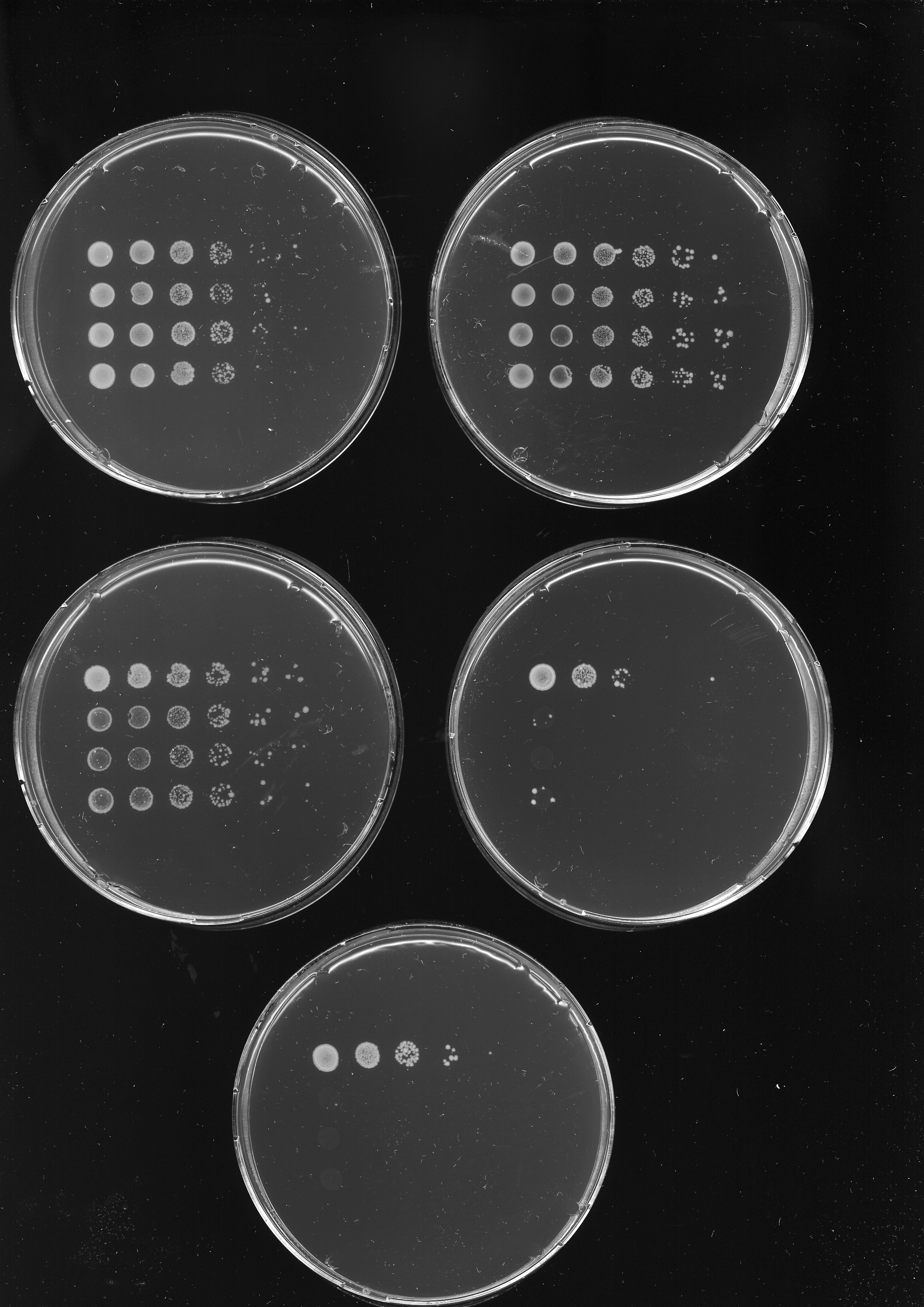

Supplement: Supplementary file 12 — Source data Fig. 8 [file 44318_2025_575_MOESM12_ESM.zip › Figure 8/exp2-40-0725.tif]

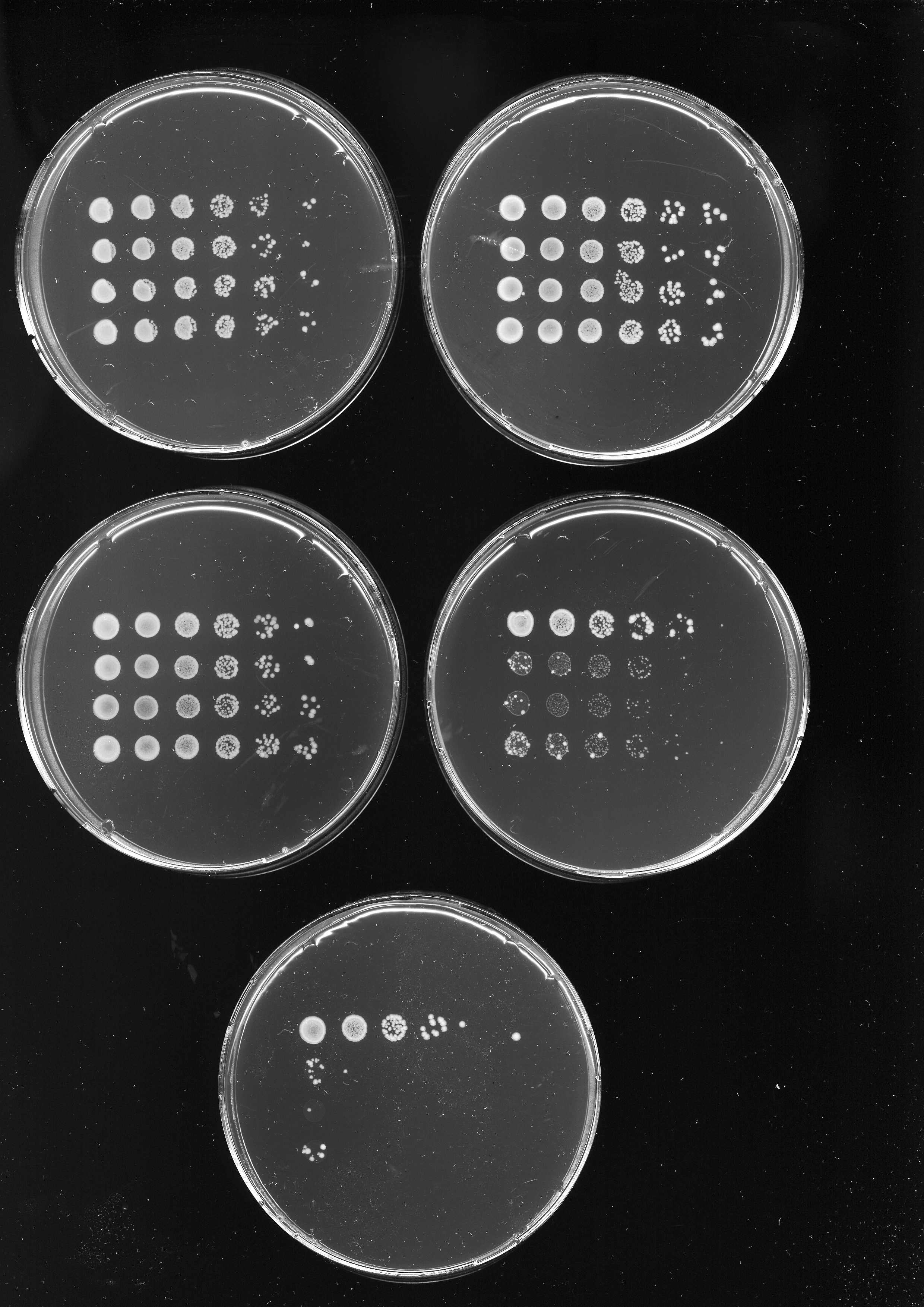

Supplement: Supplementary file 12 — Source data Fig. 8 [file 44318_2025_575_MOESM12_ESM.zip › Figure 8/exp2-37-0725.tif]

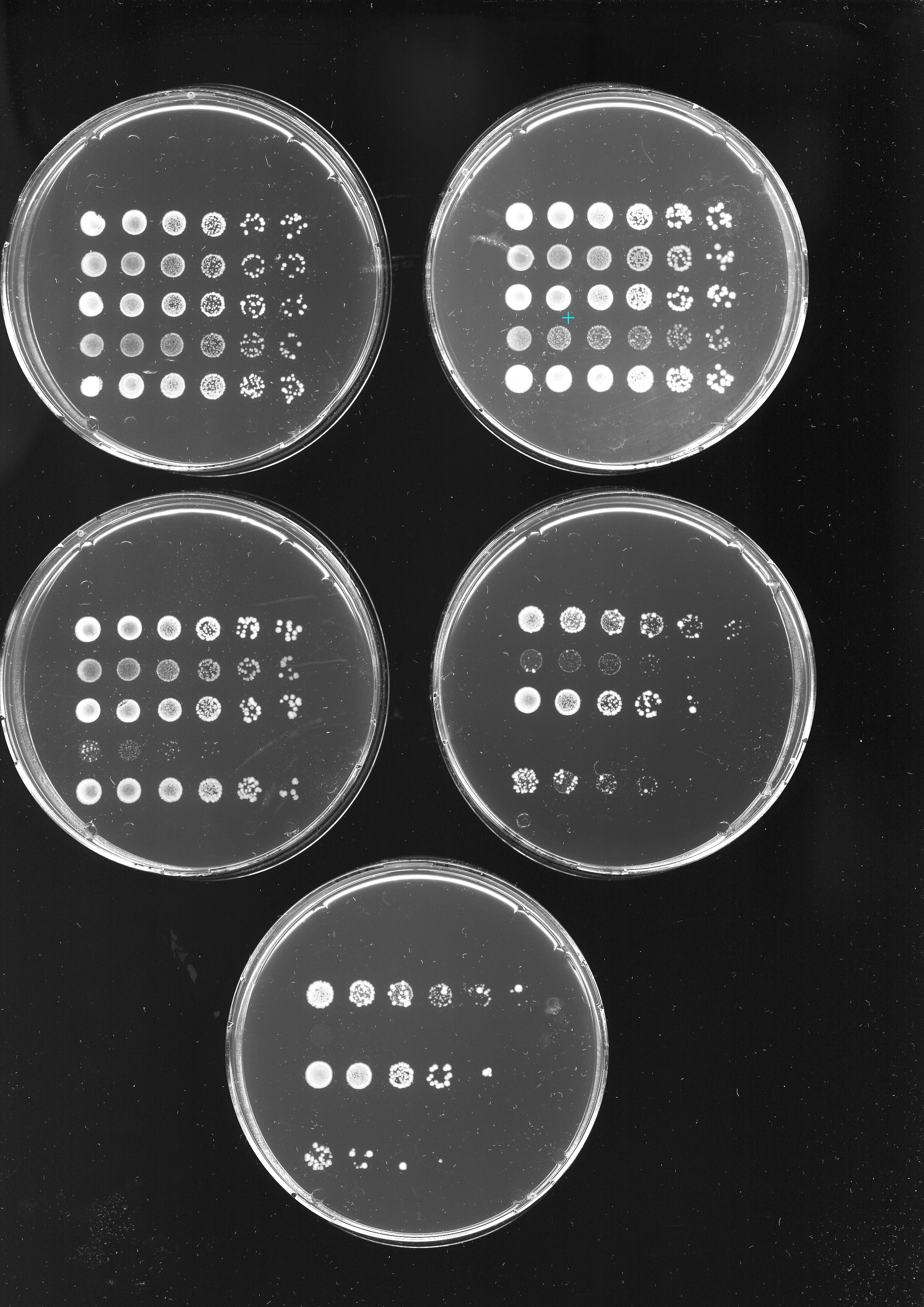

Supplement: Supplementary file 12 — Source data Fig. 8 [file 44318_2025_575_MOESM12_ESM.zip › Figure 8/exp1-37-0725.tif]

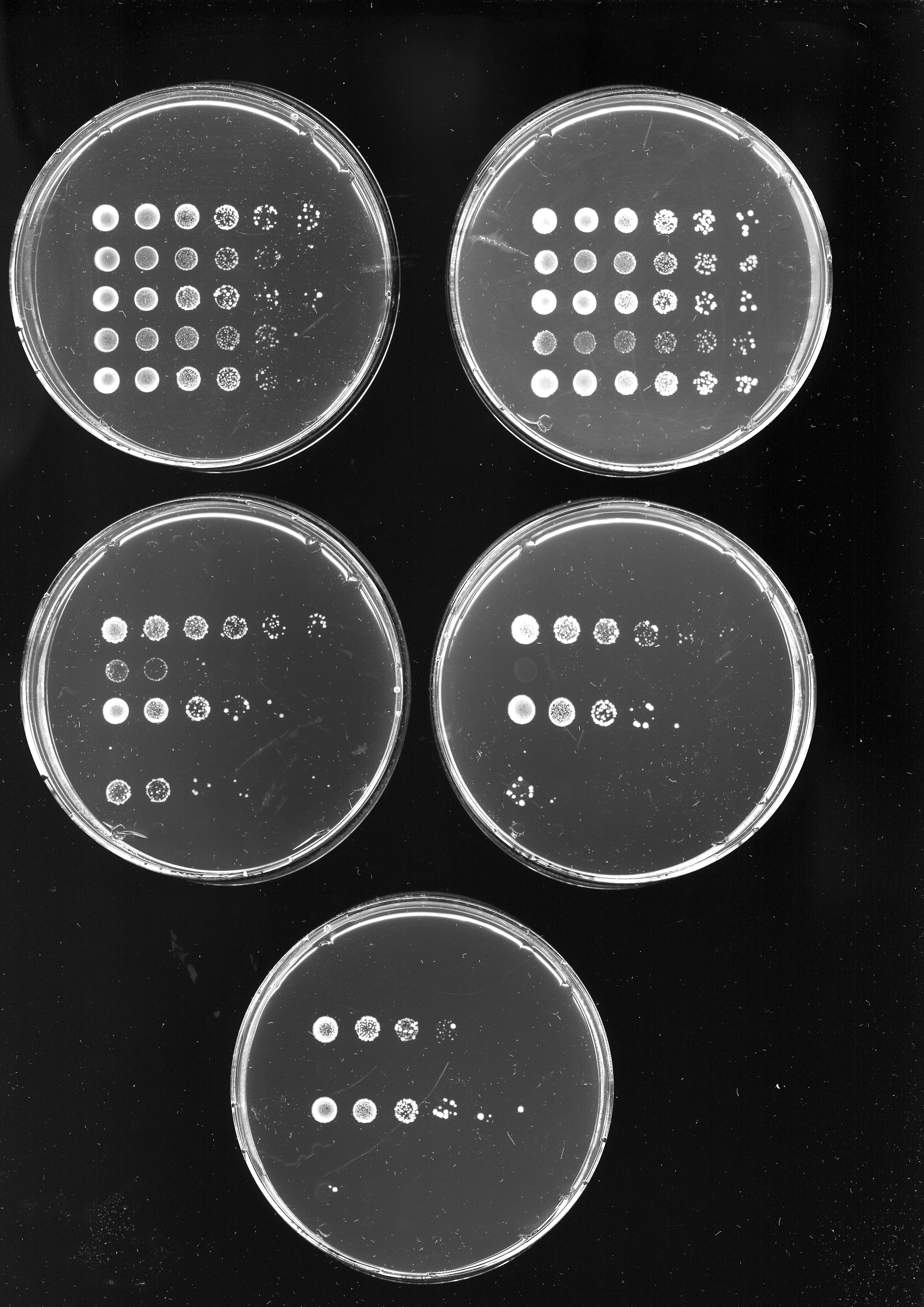

Supplement: Supplementary file 12 — Source data Fig. 8 [file 44318_2025_575_MOESM12_ESM.zip › Figure 8/exp1-40-0725.tif]

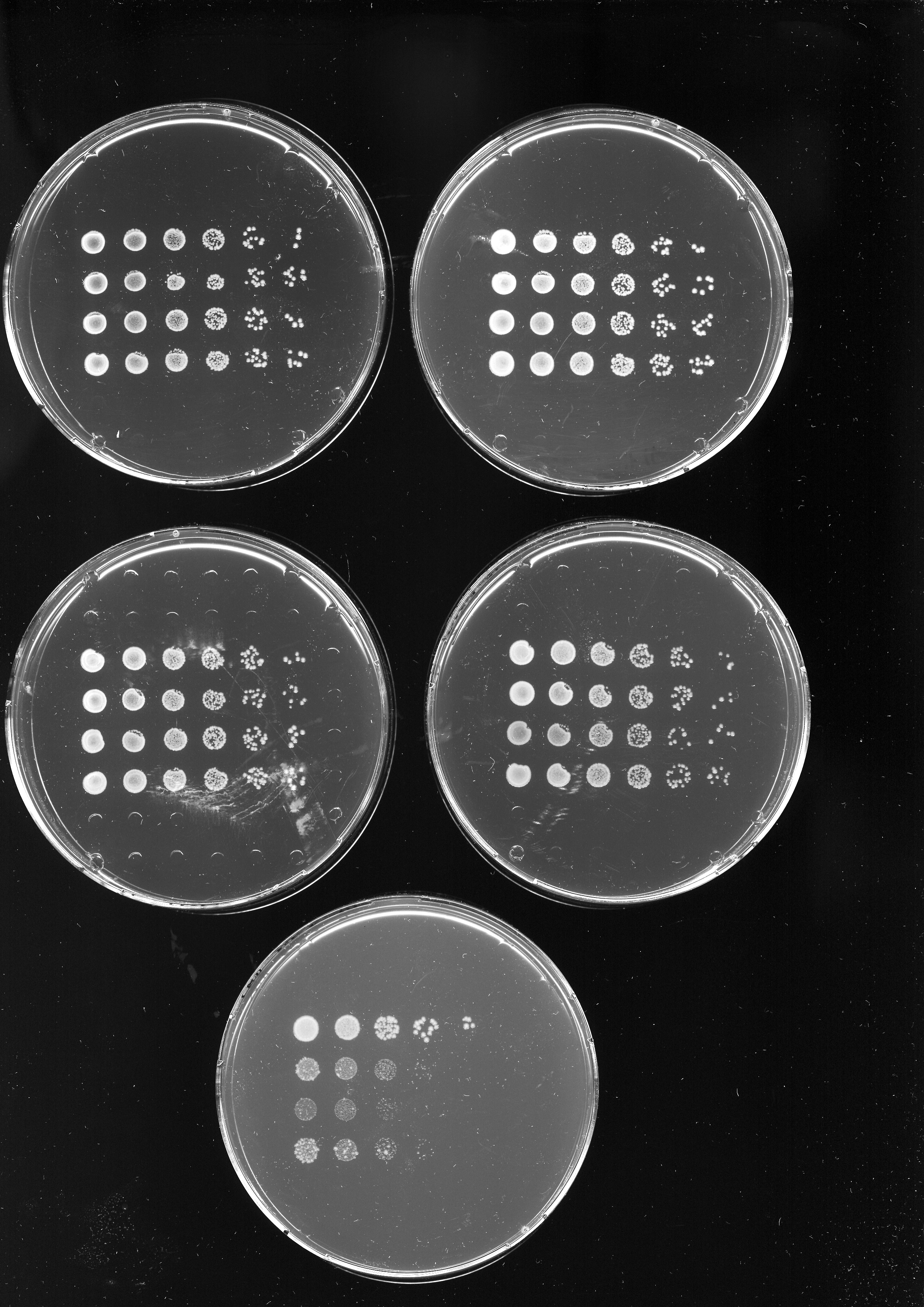

Supplement: Supplementary file 12 — Source data Fig. 8 [file 44318_2025_575_MOESM12_ESM.zip › Figure 8/exp2-30-0725.tif]
