# Supplementary material for: Allosteric control of the bacterial ClpC/ClpP protease and its hijacking by antibacterial peptides
Source: EMBO J. 2025 Sep 29;44(21):6273–96. doi: 10.1038/s44318-025-00575-1 (PMC12583610; doi:10.1038/s44318-025-00575-1)
Supplement: Supplementary file 14 — Expanded View Figures [file 44318_2025_575_MOESM14_ESM.pdf]

Expanded View Figures

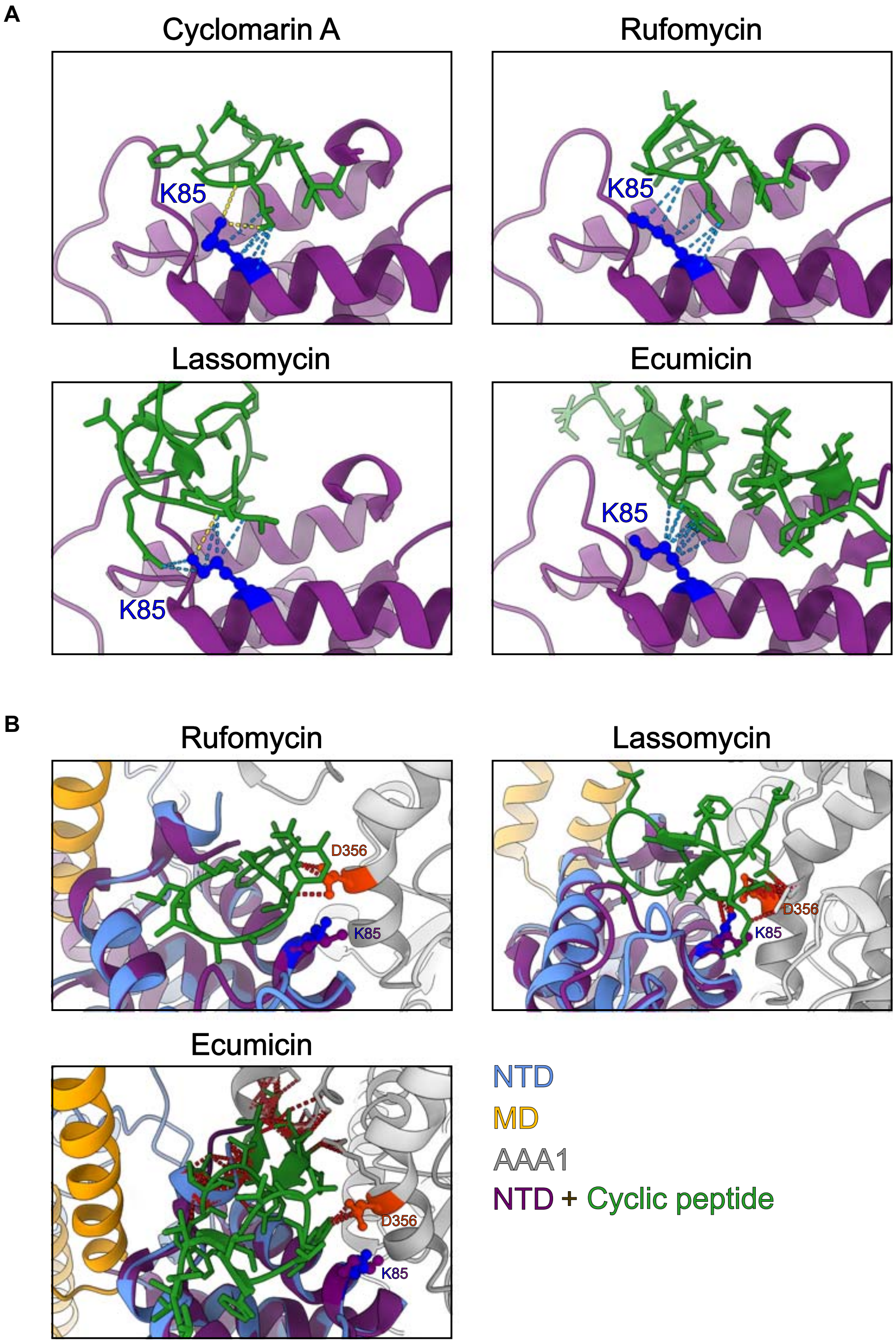

Figure EV1. Antibacterial peptides abrogate key NTD-AAA1 interaction.

(**A**) Co-crystal structures (pdbs: 3wdc, 6cn8, 8ibp, 6pbs) of indicated cyclic peptides (green) bound to the Mtb ClpC1 NTD (purple) are displayed. The NTD residue K85 is highlighted in blue and interactions with the respective cyclic peptides are indicated (H-bonds in yellow, contacts in light blue). (**B**) Co-crystal structures of Mtb ClpC1 and cyclic peptides were superimposed with an NTD of the *S. aureus* ClpC resting state. Cyclic peptides clash with the conserved AAA1 residue D356 (D364 in Mtb ClpC1), indicated with dark red dashed lines, abrogating the crucial NTD-AAA1 interaction.

© The Author(s)

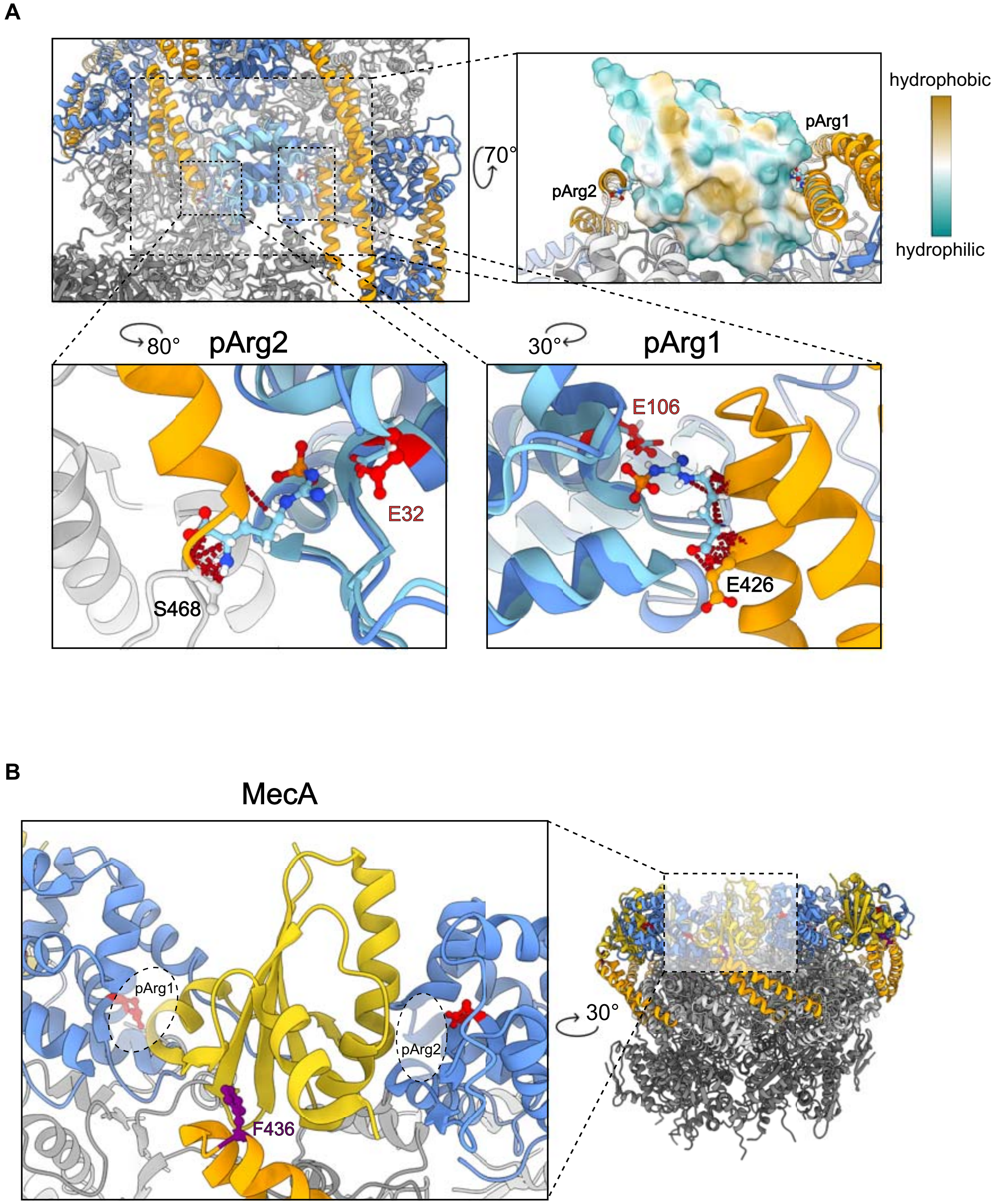

Figure EV2. Activation of ClpC by binding of pArg and MecA.

(**A**) The *B. subtilis* NTD (cyan) co-crystallized with pArg (pdb:5hbn) was superimposed with an NTD of the resting state. pArg1/2 binding sites are involved in resting state formation by contacting MDs of the same (pArg2) and a neighboring subunit (pArg1). pArg binding will abrogate these interactions leading to resting state destabilization (clashes of pArg with the MD are indicated with dark red dashed lines). Additional binding of a disordered segment of a pArg-substrate to the hydrophobic groove of the NTD is required for ClpC activation. (**B**) MecA binds to both pArg1/2 binding sites and the MD tip including the conserved residue F436, thereby shielding major sites involved in resting state formation (pdb:3j3s).

 © The Author(s)
